# Supplementary material for: Integrative differential expression and gene set enrichment analysis using summary statistics for scRNA-seq studies
Source: Nat Commun. 2020 Mar 27;11:1585. doi: 10.1038/s41467-020-15298-6 (PMC7101316; doi:10.1038/s41467-020-15298-6)
Supplement: Supplementary file 2 — Supplementary Information [file 41467_2020_15298_MOESM2_ESM.pdf]

Supplementary Information

Integrative Differential Expression and Gene Set Enrichment Analysis Using Summary Statistics for scRNA-seq Studies

Ma, Sun et al.

Supplementary Figures

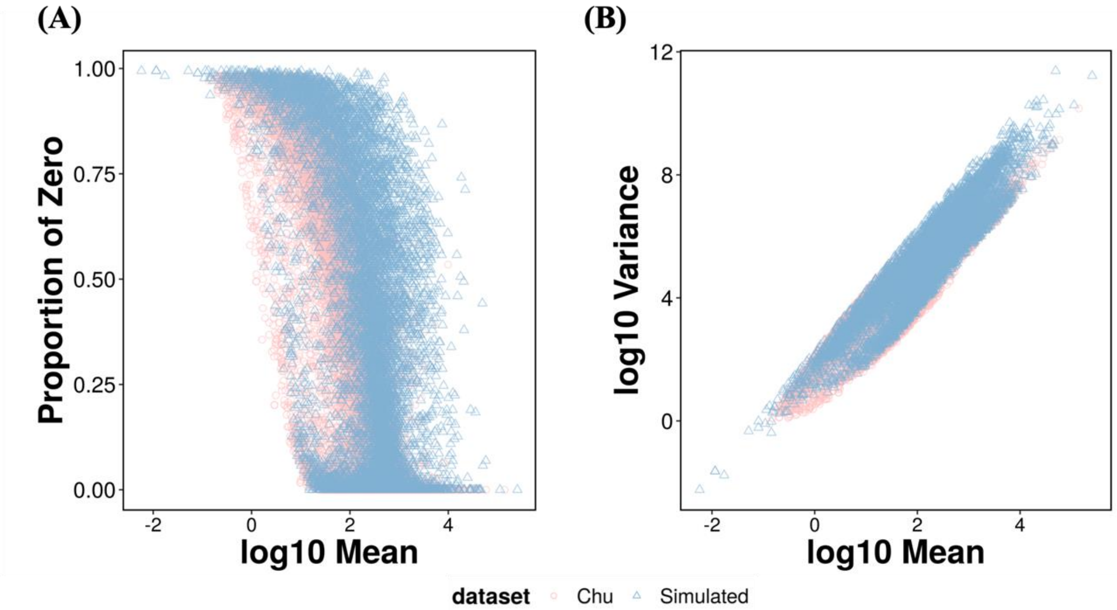

Supplementary Figure 1. Simulated data has very similar characteristics as compared to the real scRNA-seq dataset. The data was simulated under the following parameters setting: $\tau_0 = -2, \tau_1 = 0.5$ and $\mathrm{CR} = 10\%$. Proportion of zero versus mean under log10 scale for both simulated data (blue) and real data (pink) (**A**); Mean-variance plot under log10 scale for both simulated data (blue) and real data (pink) (**B**);

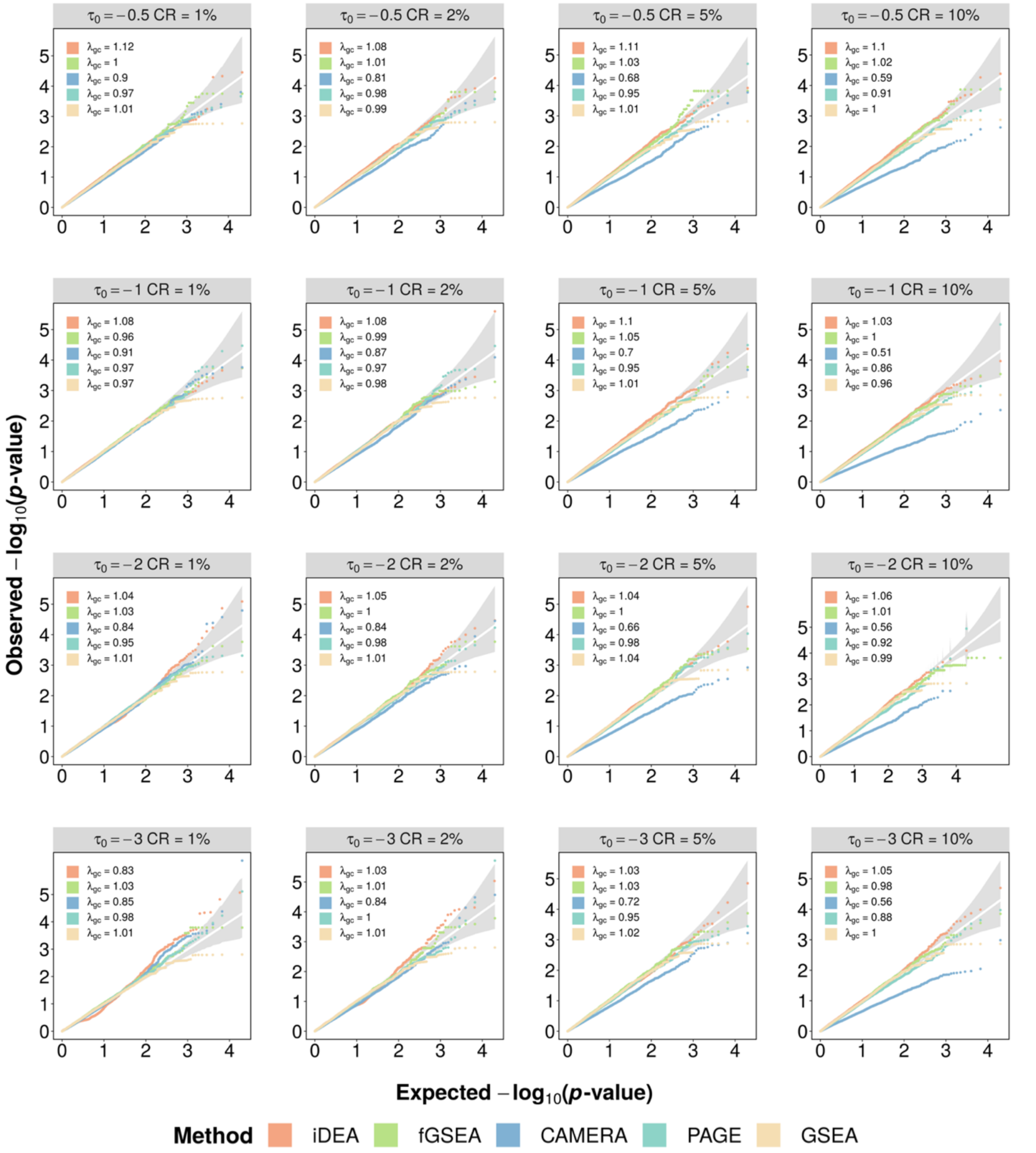

Supplementary Figure 2. iDEA produces well-calibrated *p*-values for gene set enrichment analysis under null simulation. Quantile-quantile plots of -log10(*p*-values) from iDEA (orange), fGSEA (green), CAMERA (navyblue), PAGE (skyblue) and GSEA (yellow) are shown under different null scenarios with varying number of DE genes (denoted by the odd parameter τ_0; -0.5, -1.0, -2.0, or -3.0) and gene set coverage rates (CR; 1%, 2%, 5% or 10%). CR represents the percentage of genes inside the gene set. λ_{gc} is genomic control factor.

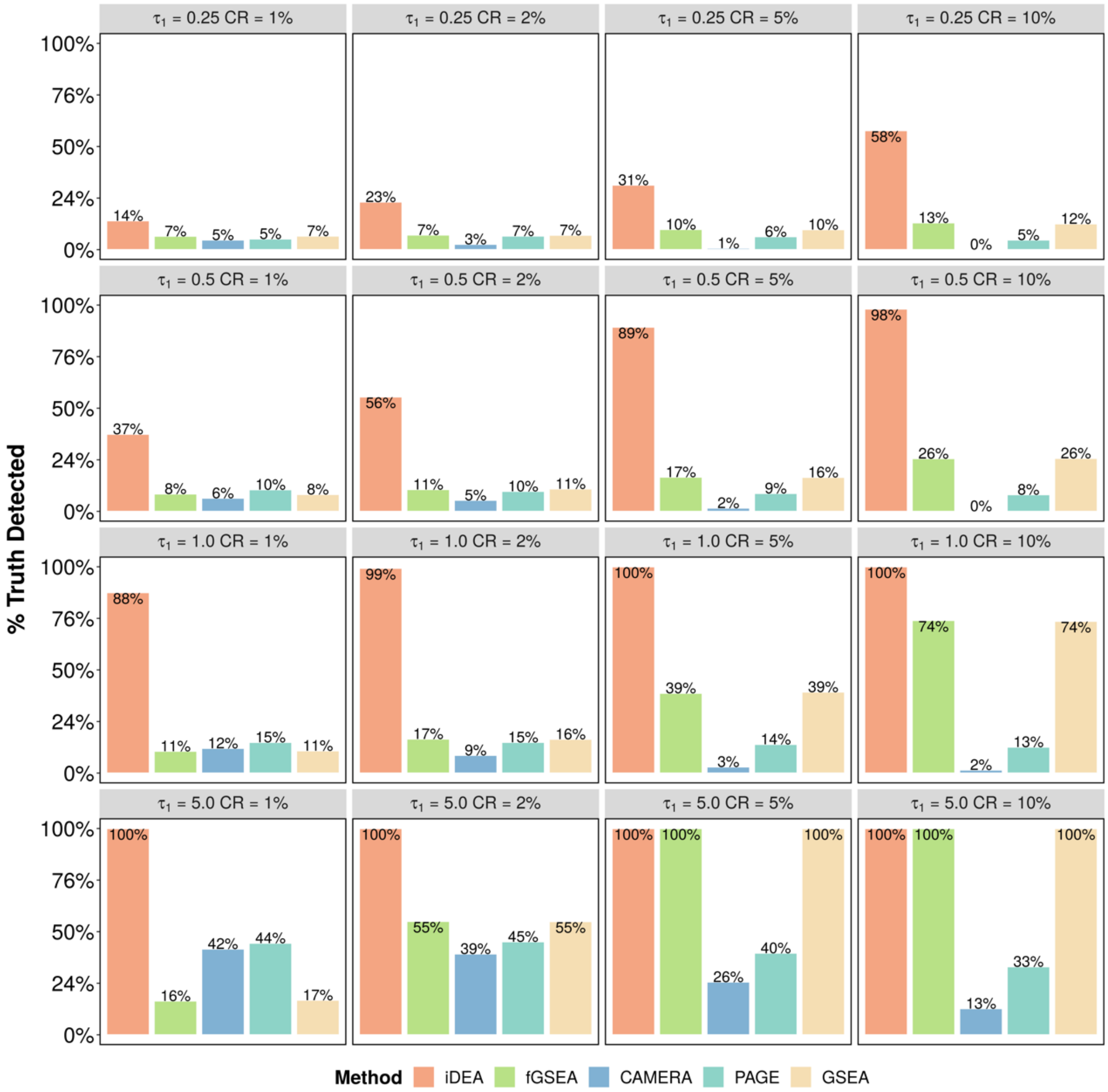

Supplementary Figure 3. iDEA is more powerful than GSE methods for identifying enriched gene sets under alternative simulations. The power plots from iDEA (orange), fGSEA (green), CAMERA (navyblue), PAGE (skyblue) and GSEA (yellow) are shown under different scenarios with varying gene set enrichment coefficient (denoted by the odd parameter τ_1; 0.25, 0.5, 1.0 or 5.0) and gene set coverage rates (CR; 1%, 2%, 5% or 10%). CR represents the percentage of genes inside the gene set. Here, power was calculated based on an FDR of 5%.

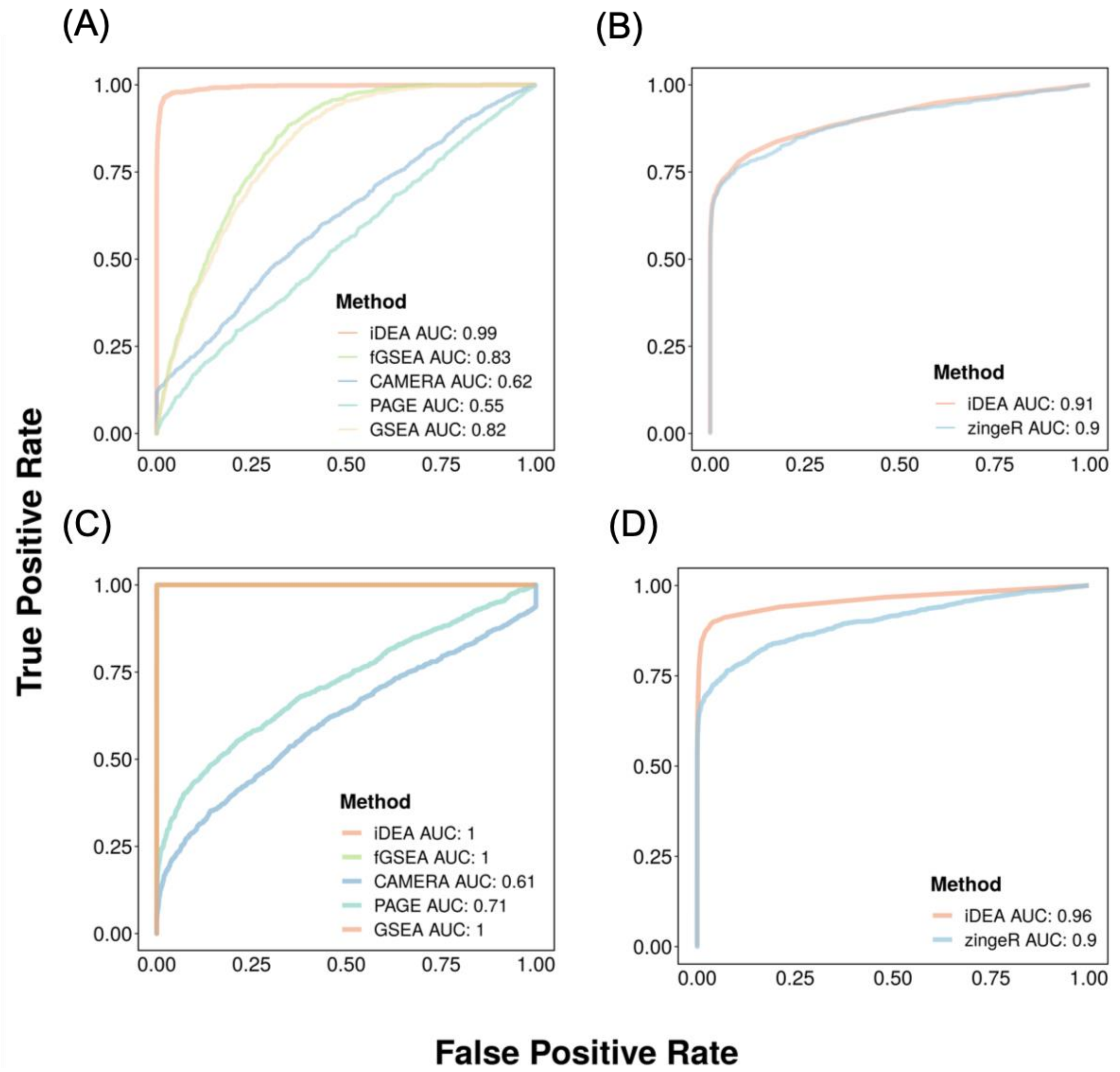

Supplementary Figure 4. iDEA is more powerful for both GSE and DE analyses than existing approaches in power simulations. The AUC of iDEA in identifying enriched pathways (**A** and **C**) and in identifying differentially expressed genes (**B** and **D**) are higher than that of the other methods. The compared GSE methods (**A** and **C**) include iDEA (orange), fGSEA (green), CAMERA (navyblue), PAGE (skyblue) and GSEA (yellow). The compared DE methods (**B** and **D**) include iDEA (orange) and zingeR (skyblue). Simulations are performed under two parameter settings: $\tau_0 = -2$, $\tau_1 = 0.5$, and CR = 10% (**A** and **B**); $\tau_0 = -2$, $\tau_1 = 5$, and CR = 10% (**C** and **D**).

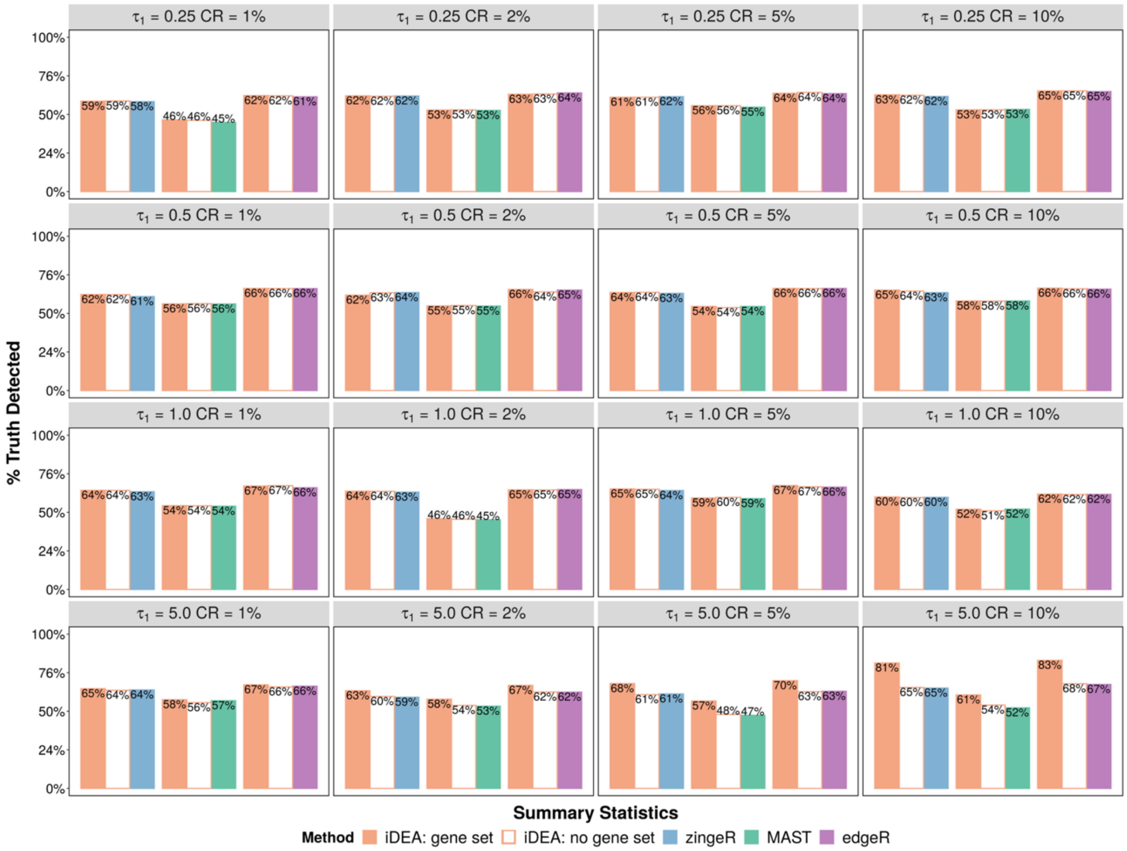

Supplementary Figure 5. iDEA is more powerful than DE methods for identifying DE genes under alternative simulations when gene set enrichment parameter is larger. Simulations were performed on one fixed scRNA-seq data set with $\tau_0 = -2$, varying τ_1 and CR. τ_1 is set to be 0.25, 0.5,1.0 or 5.0 and CR is set to be 1%, 2%, 5%, 10% respectively. In each simulation setting, power of DE results between common DE method (zingeR (blue), MAST (green), edgeR (purple)) and iDEA (orange) with summary statistics obtained from that corresponding DE method when adding simulated gene set (filling color) or not (not filling color) is plotted. Here, power was calculated based on an FDR of 5%.

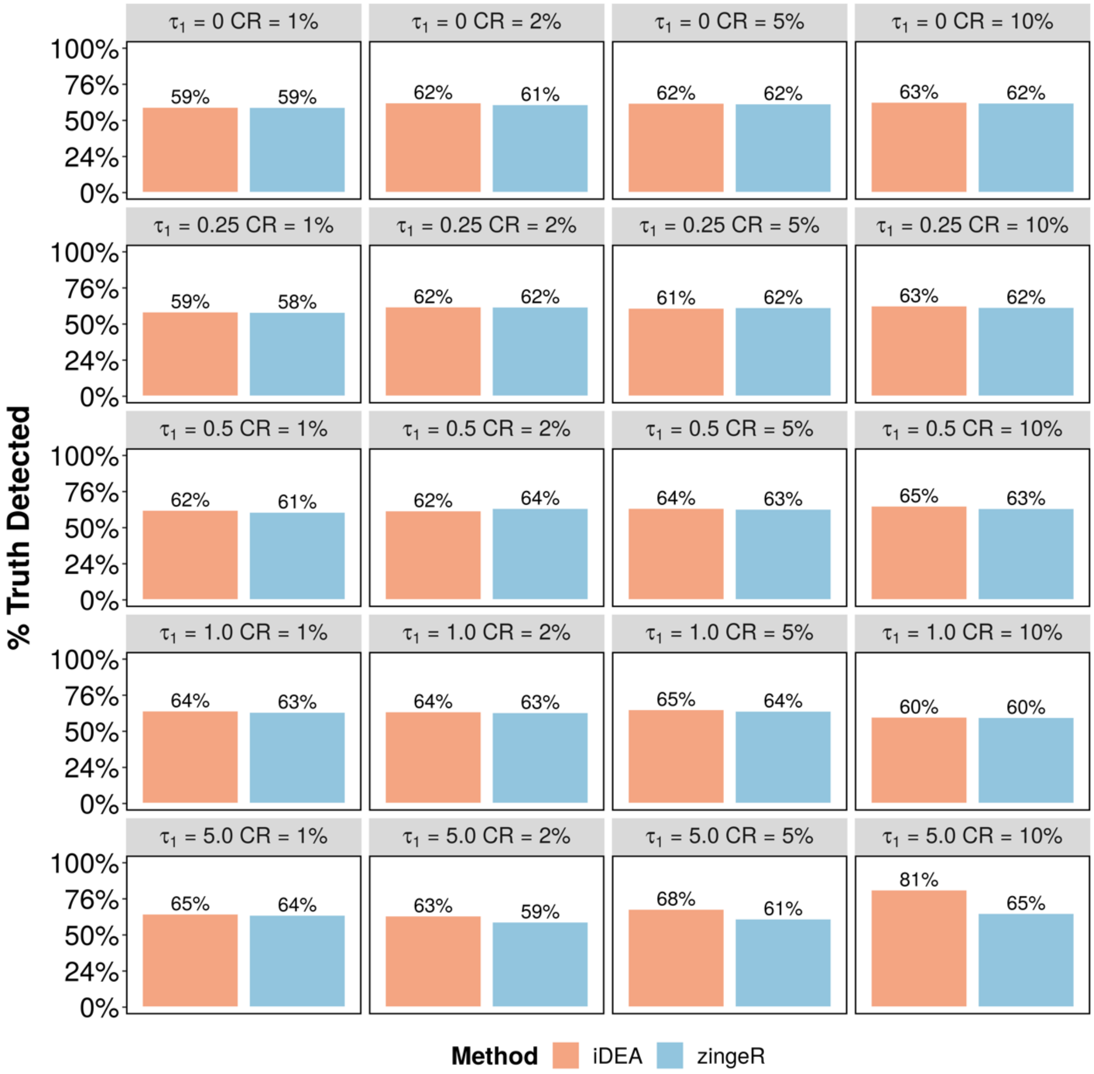

Supplementary Figure 6. iDEA provides the powerful performance on DE analysis when varying gene set enrichment coefficient τ_1 and coverage rate CR than zingeR especially when gene set enrichment parameter is higher. The data were simulated based on the parameter setting $\tau_0 = -2$, $\tau_1 = 0$, 0.25, 0.5, 1.0 or 5.0 and CR = 1%, 2%, 5% or 10%. iDEA identifies more significant gene sets on simulation studies when varying parameters. CR represents the percentage of genes inside the gene set. Here, power was calculated based on an FDR of 5%.

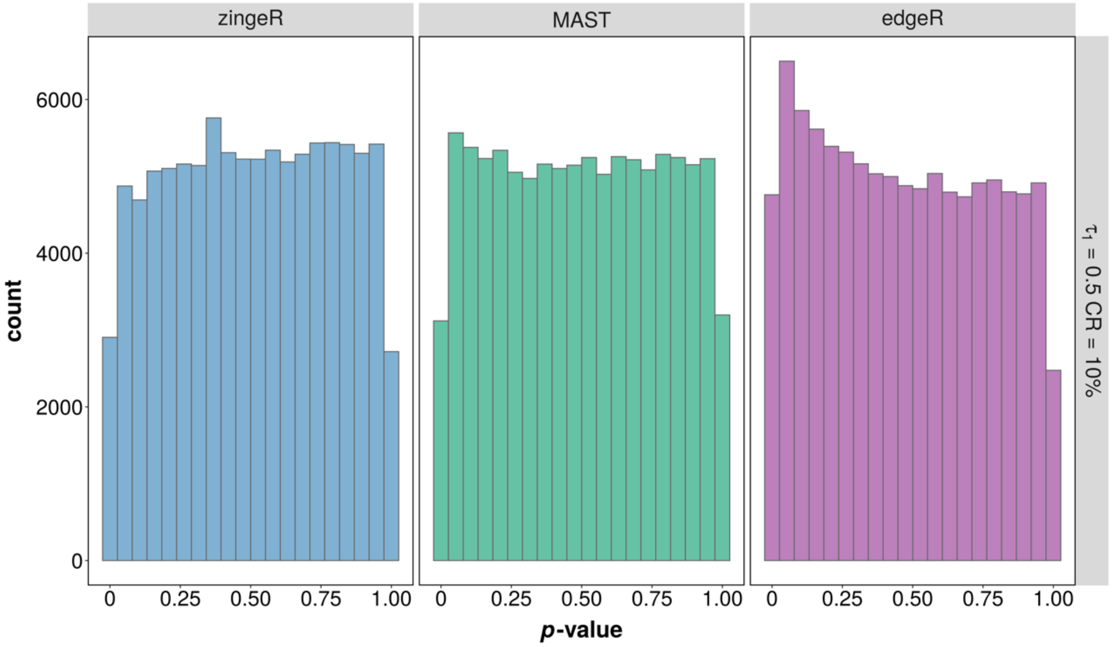

Supplementary Figure 7. Distribution of marginal DE *p*-values from common DE methods. The data were simulated based on the parameter setting $\tau_0 = -2$, $\tau_1 = 0.5$ and CR = 10%. *P*-values from zingeR (blue) and MAST (green) follow approximately a uniform distribution under the null while *P*-values from edgeR (purple) does not.

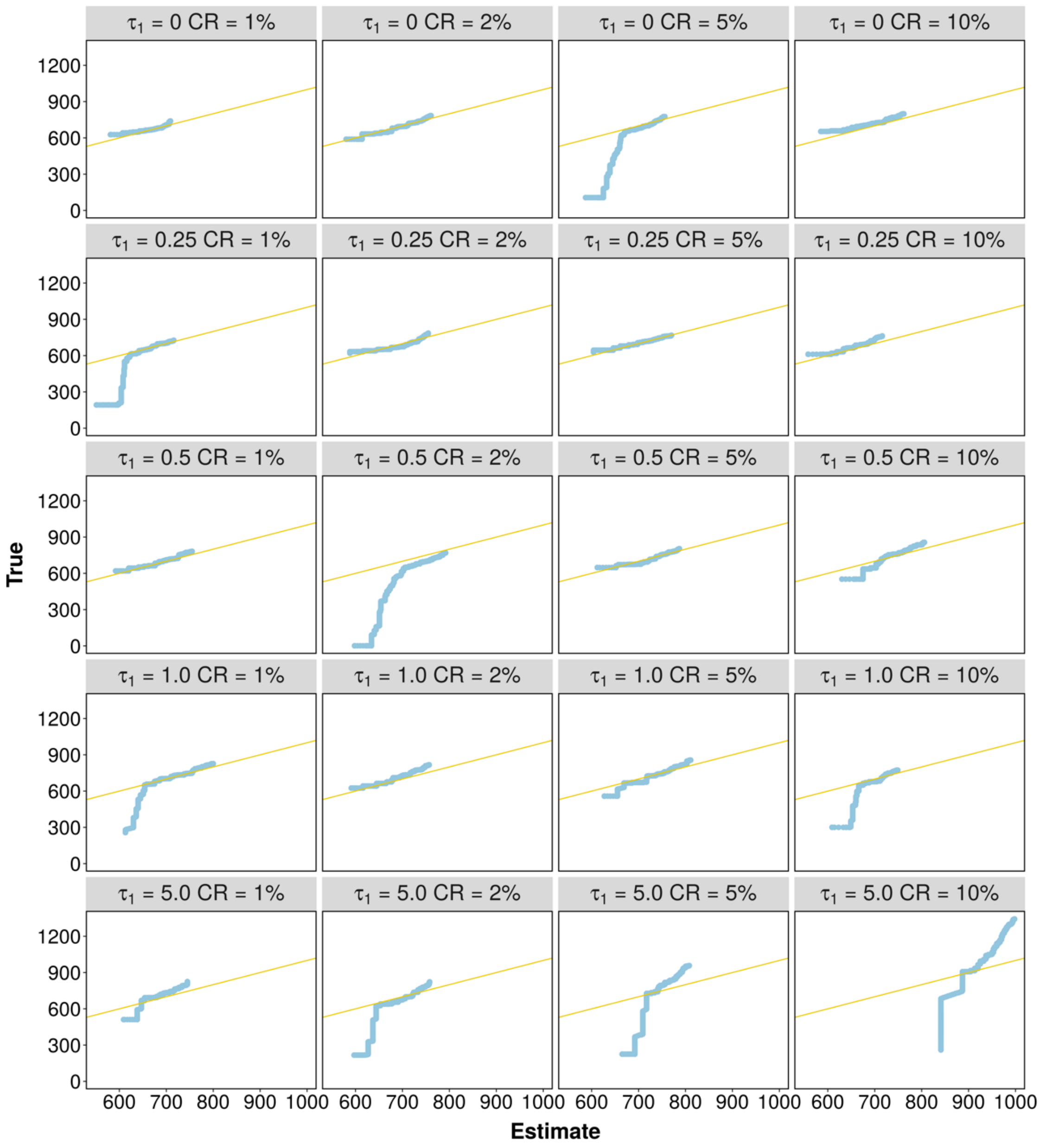

Supplementary Figure 8. iDEA produces calibrated (or slightly conservative) FDR estimates. Simulations were performed on one fixed scRNA-seq data set with $\tau_0 = -2$, varying τ_1 and CR. τ_1 is set to be 0, 0.25, 0.5,1.0 or 5.0 and CR is set to be 1%, 2%, 5%, 10% respectively. In each simulation setting, the scatterplot plot showed the number of detected signals based on true FDR (y-axis) versus the number of detected signals based on estimated FDR (x-axis). The yellow line is the reference line which represents y = x.

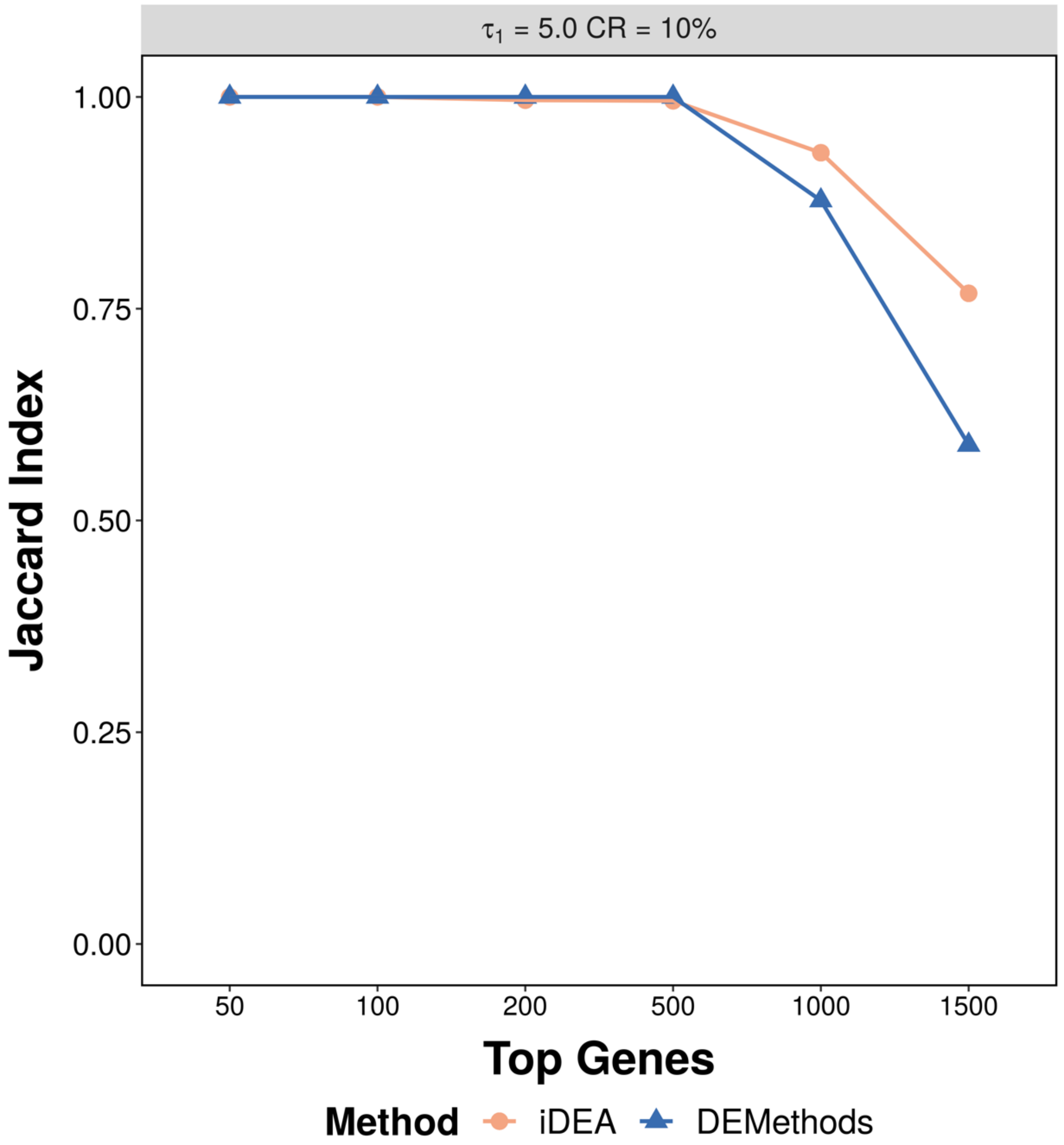

Supplementary Figure 9. iDEA displays high consistency in detecting DE genes in simulations. The data were simulated based on the parameter setting $\tau_0 = -2$, $\tau_1 = 5$, and CR = 10%. The plot shows the Jaccard index for top DE genes between zingeR, edgeR and MAST (blue) and the Jaccard index for top DE genes between iDEA when using summary statistics from zingeR, edgeR and MAST respectively(orange). CR represents the percentage of genes inside the gene set, τ_0 represents number of DE genes and τ_1 represents the gene set enrichment coefficient.

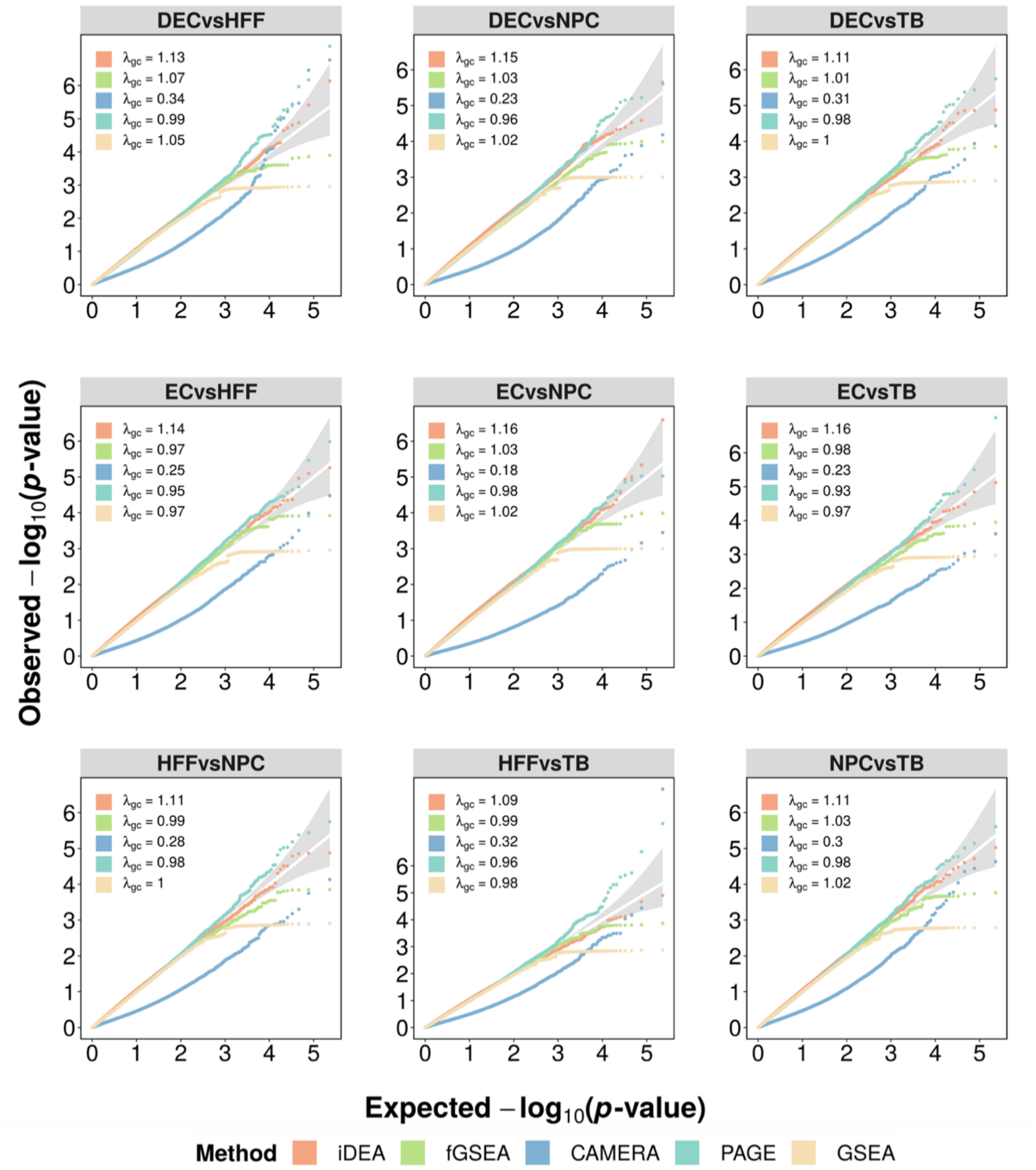

Supplementary Figure 10. iDEA produces well-calibrated *p*-values in pairwise cell type comparison on human embryonic stem cell scRNA-seq data. Quantile-quantile plots of -log10(*p*-values) from iDEA (orange), fGSEA (green), CAMERA (navyblue), PAGE (skyblue) and GSEA (yellow) are shown under the null that permuted each gene set 10 times correspondingly. λ_{gc} is genomic control factor.

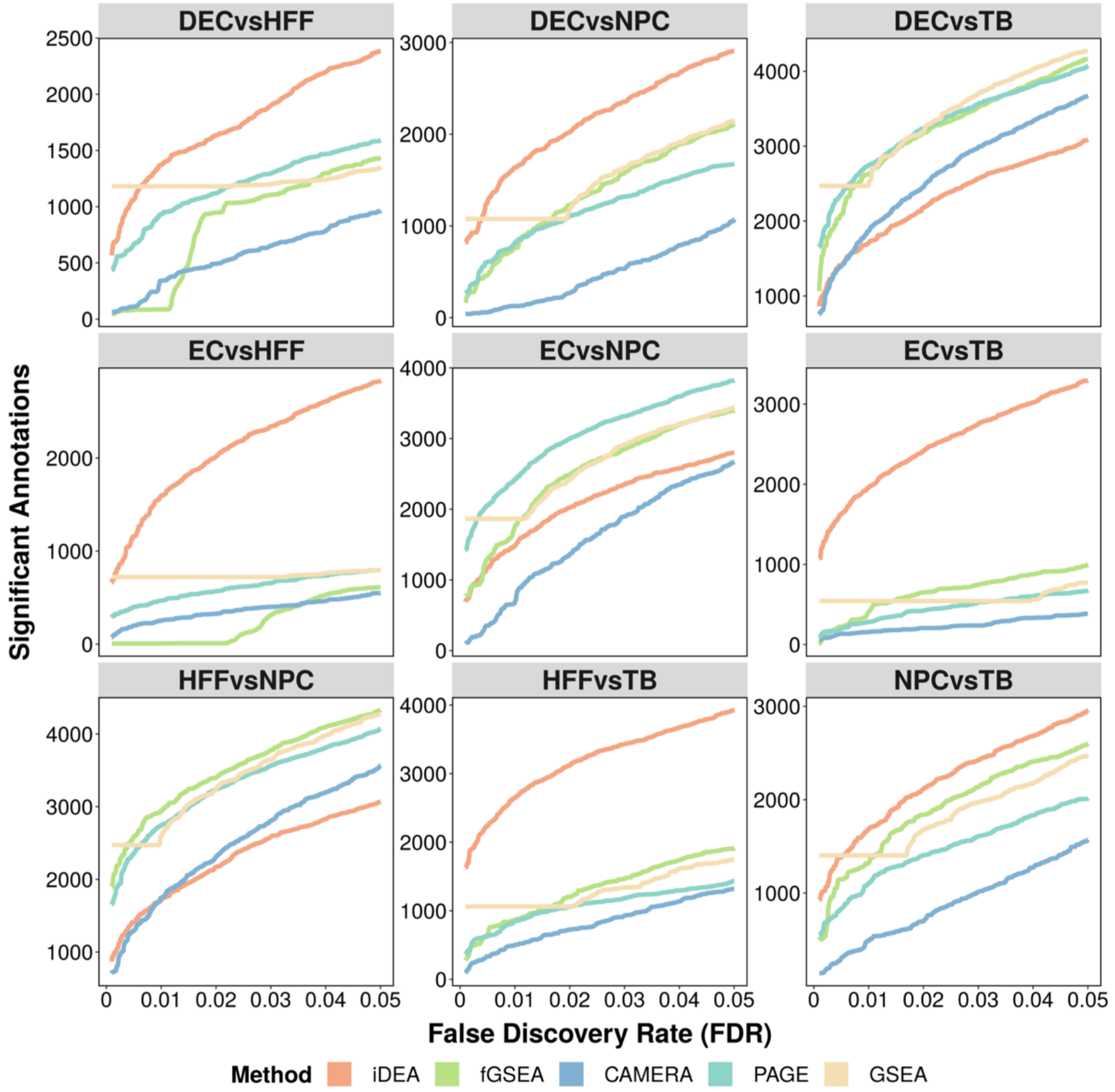

Supplementary Figure 11. iDEA is more powerful than GSE methods for identifying enriched gene sets in pairwise cell-type comparison on human embryonic stem cell scRNA-seq dataset. The power plots from iDEA (orange), fGSEA (green), CAMERA (navyblue), PAGE (skyblue) and GSEA (yellow) are shown under different cell type comparisons.

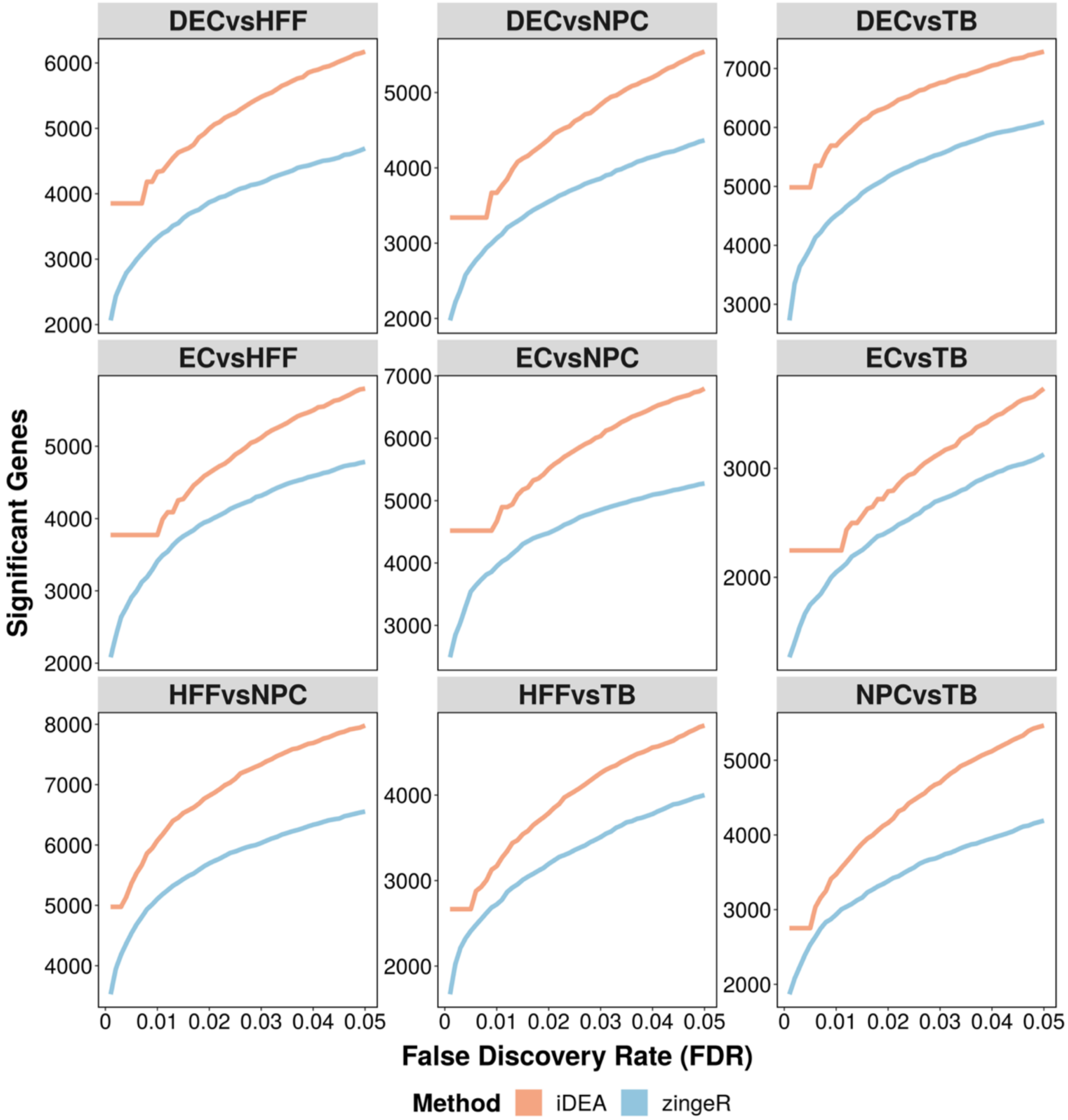

Supplementary Figure 12. iDEA provides the powerful performance on DE analysis when adding one biologically meaningful gene set in pairwise cell type comparison on human embryonic stem cell scRNA-seq dataset than zingeR. Number of identified DE genes by iDEA (orange) and zingeR (blue) are plotted against different empirical FDR values.

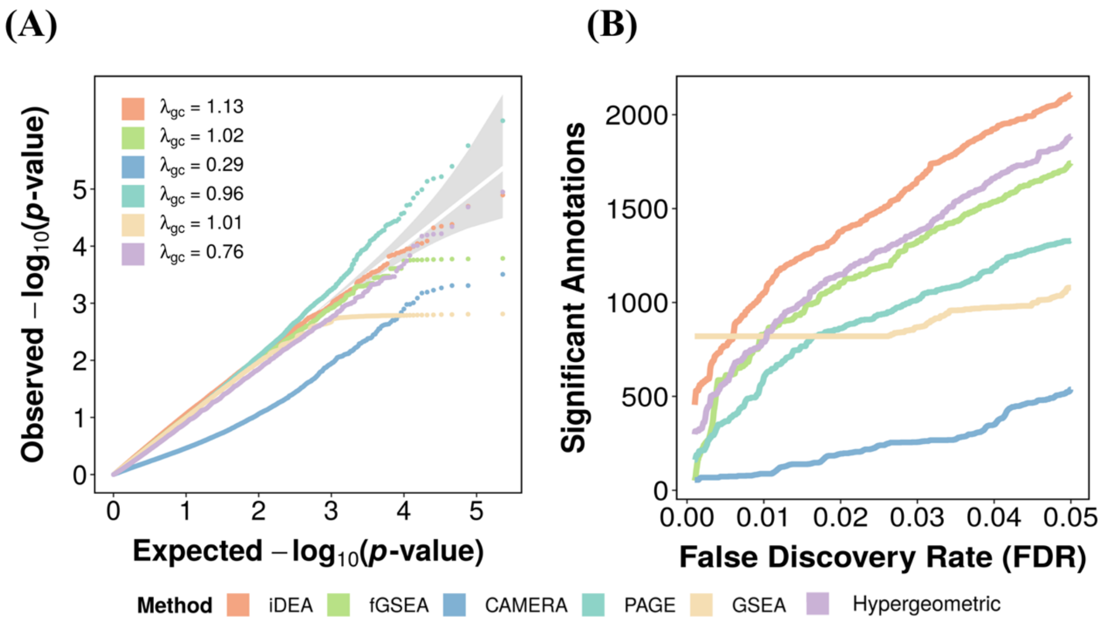

Supplementary Figure 13. GSE Analysis including hypergeometric test results in human embryonic stem cell scRNA-seq dataset. Results are shown for comparing definitive endoderm derivatives cell (DEC, 138 cells) and endothelial cell (EC, mesoderm derivatives, 105 cells). Quantile-quantile plots of -log10(*p*-values) from GSE methods including iDEA (orange), fGSEA (green), CAMERA (navyblue), PAGE (skyblue), GSEA (yellow) and Hypergeometric test (purple) are shown under permuted null (**A**); Number of identified enriched gene sets by iDEA (orange), fGSEA (green), CAMERA (navyblue), PAGE (skyblue) GSEA (yellow) and Hypergeometric test (purple) are plotted against different empirical false discovery rates (FDR) (**B**). Here λ_{gc} is the genomic control factor.

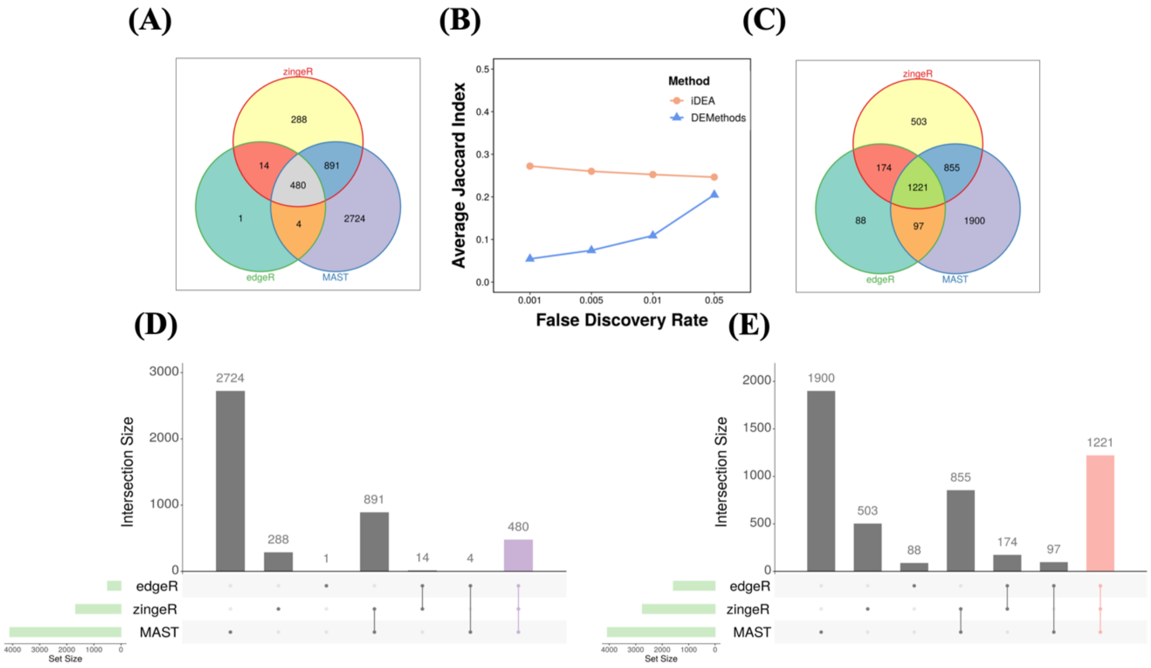

Supplementary Figure 14. iDEA displays high consistency in detecting DE genes in human embryonic stem cell scRNA-seq data. iDEA displays higher Jaccard index in the common DE genes. Jaccard index for top DE genes at an FDR of 1% between zingeR, MAST and edgeR, Jaccard index for top DE genes at an FDR of 1% between iDEA when using summary statistics from zingeR, MAST and edgeR, respectively. (**B**); Overlap in top DE genes at an FDR of 1% between zingeR, MAST and edgeR **(A)**; Overlap in top DE genes at an FDR of 1% between iDEA when using summary statistics from zingeR, MAST and edgeR (**C**); **(D)** and **(E)** are just another visualization of the overlap corresponding to **(A)** and **(C)** by UpSetR[1].

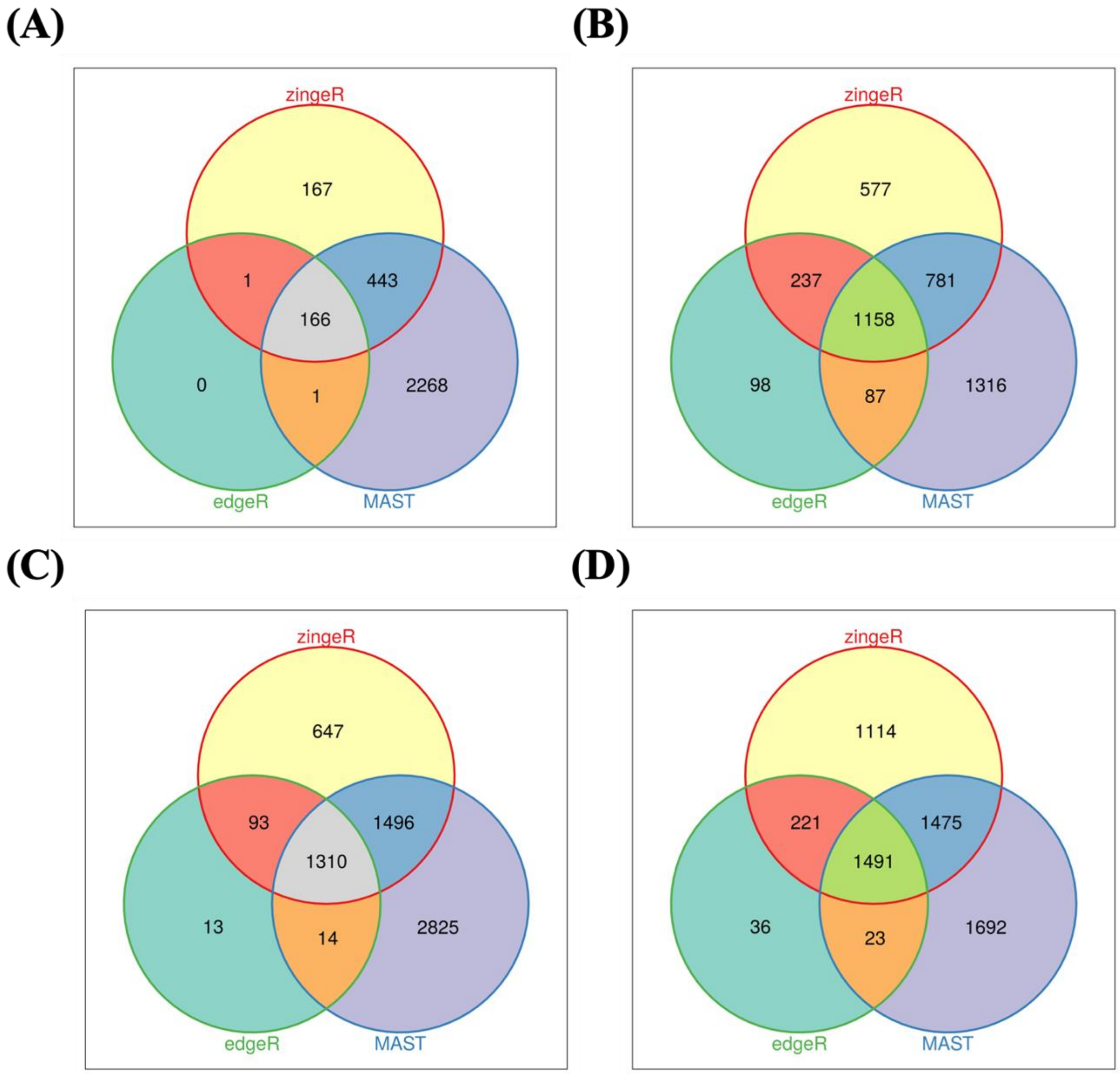

Supplementary Figure 15. iDEA displays high consistency in detecting DE genes in human embryonic stem cell scRNA-seq data. Overlap in top DE genes at an FDR of 0.1% between zingeR, MAST and edgeR (**A**); Overlap in top DE genes at an FDR of 0.1% between iDEA when using summary statistics from zingeR, MAST and edgeR respectively (**B**); Overlap in top DE genes at an FDR of 5% between zingeR, MAST and edgeR (**C**); Overlap in top DE genes at an FDR of 5% between iDEA when using summary statistics from zingeR, MAST and edgeR, respectively (**D**);

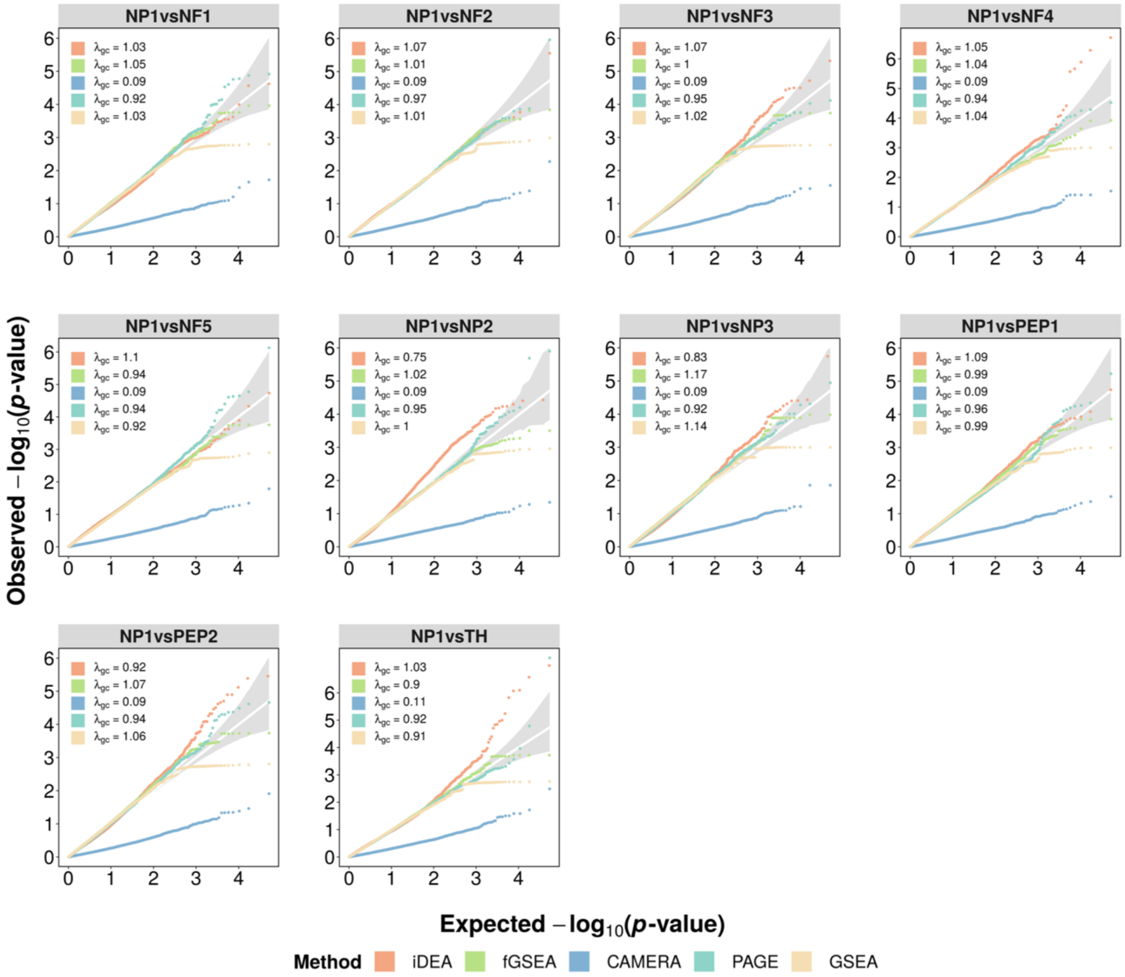

Supplementary Figure 16. iDEA produces well-calibrated *p*-values in pairwise cell type comparison on mouse neuronal cell scRNA-seq datasets. Quantile-quantile plots of -log10(*p*-values) from iDEA (orange), fGSEA (green), CAMERA (navyblue), PAGE (skyblue) and GSEA (yellow) are shown under the null that permuted each gene set 10 times correspondingly. λ_{gc} is genomic control factor.

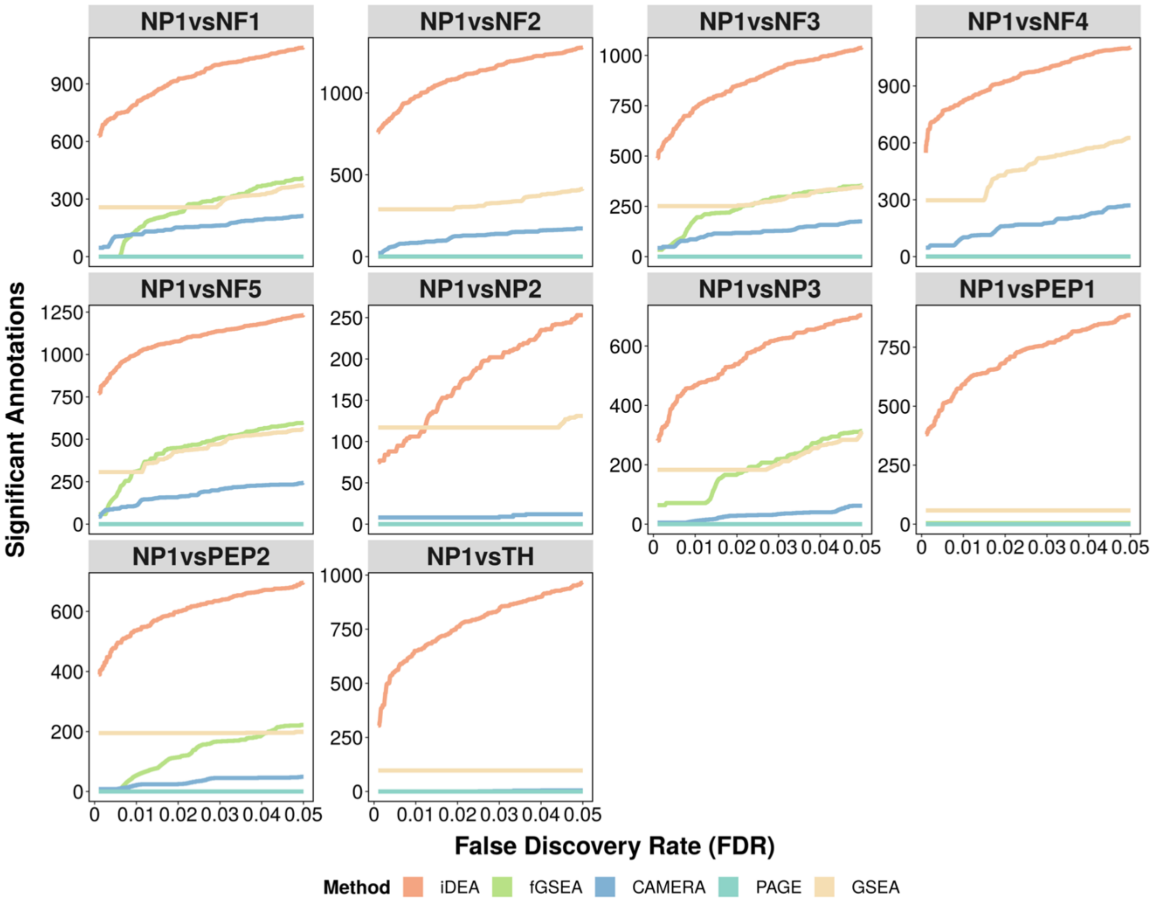

Supplementary Figure 17. iDEA is more powerful than GSE methods for identifying enriched gene sets in pairwise cell-type comparison on mouse neuronal cell scRNA-seq dataset. The power plots from iDEA (orange), fGSEA (green), CAMERA (navyblue), PAGE (skyblue) and GSEA (yellow) are shown under different cell type comparisons.

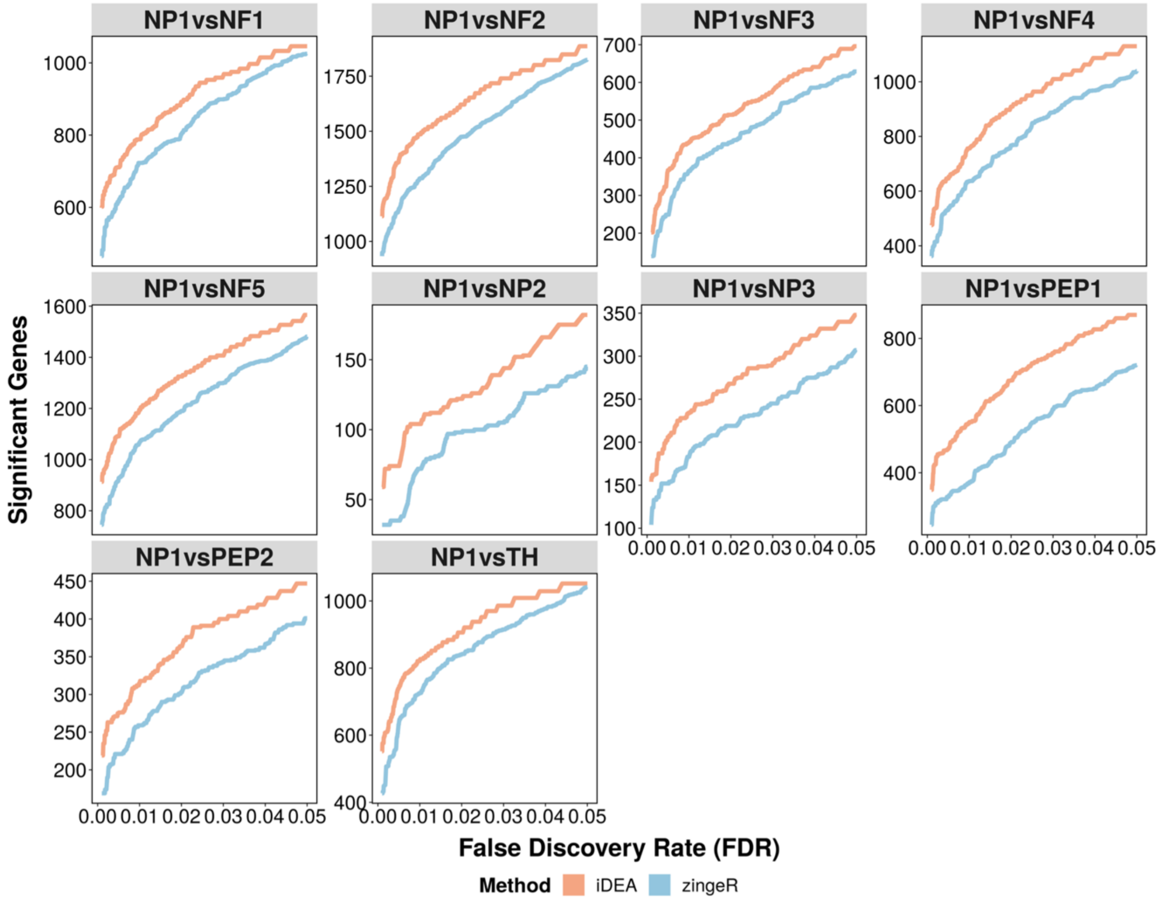

Supplementary Figure 18. iDEA provides the powerful performance on DE analysis when adding one biologically meaningful gene set in pairwise cell type comparison on mouse neuronal cell scRNA-seq dataset than zingeR. Number of identified DE genes by iDEA (orange) and zingeR (blue) are plotted against different empirical FDR values.

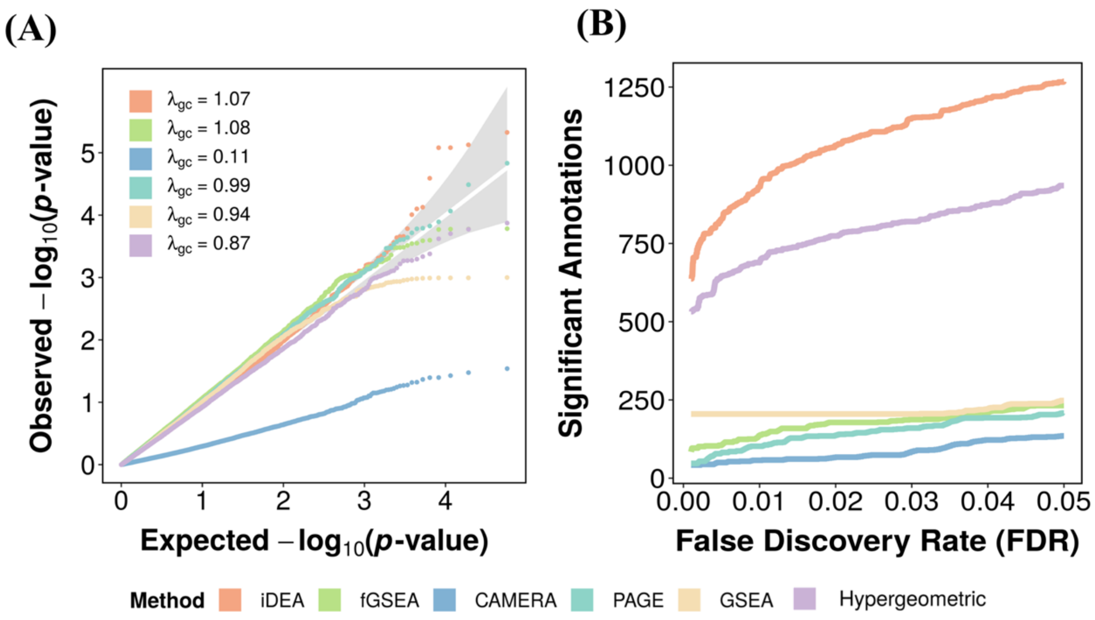

Supplementary Figure 19. GSE Analysis including hypergeometric test results in mouse neuronal cell scRNA-seq dataset. Results are shown for comparing nonpeptidergic nociceptors 1 (NP1) versus all the other cell types. Quantile-quantile plots of -log10(*p*-values) from GSE methods including iDEA (orange), fGSEA (green), CAMERA (navyblue), PAGE (skyblue), GSEA (yellow) and Hypergeometric test (purple) are shown under permuted null (**A**). Number of identified enriched gene sets by iDEA (orange), fGSEA (green), CAMERA (navyblue), PAGE (skyblue) GSEA (yellow) and Hypergeometric test (purple) are plotted against different empirical false discovery rates (FDR) (**B**). Here λ_{gc} is the genomic control factor.

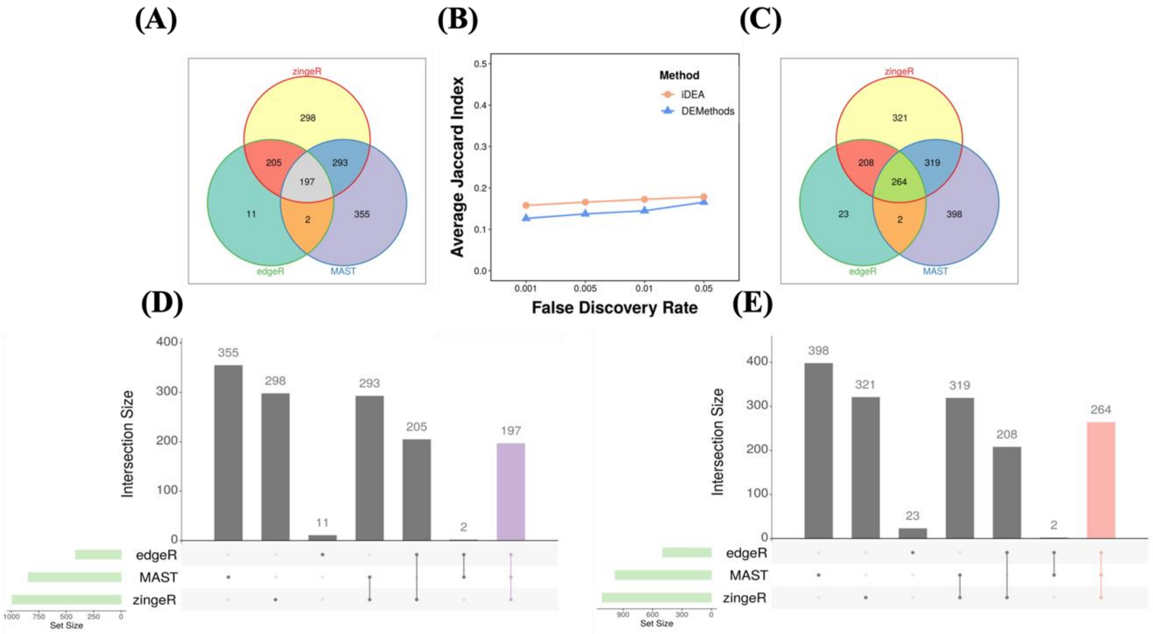

Supplementary Figure 20. iDEA displays high consistency in detecting DE genes in mouse neuronal cell scRNA-seq data. iDEA displays higher Jaccard index in the common DE genes. Jaccard index for top DE genes at an FDR of 1% between zingeR, MAST and edgeR, Jaccard index for top DE genes at an FDR of 1% between iDEA when using summary statistics from zingeR, MAST and edgeR respectively(red) (**B**); Overlap in top DE genes at an FDR of 1% between zingeR, MAST and edgeR **(A**); Overlap top DE genes at an FDR of 1% between iDEA when using summary statistics from zingeR, MAST and edgeR (**C**); **(D)** and **(E)** are just another visualization of the overlap corresponding to **(A)** and **(C)** by UpSetR[1].

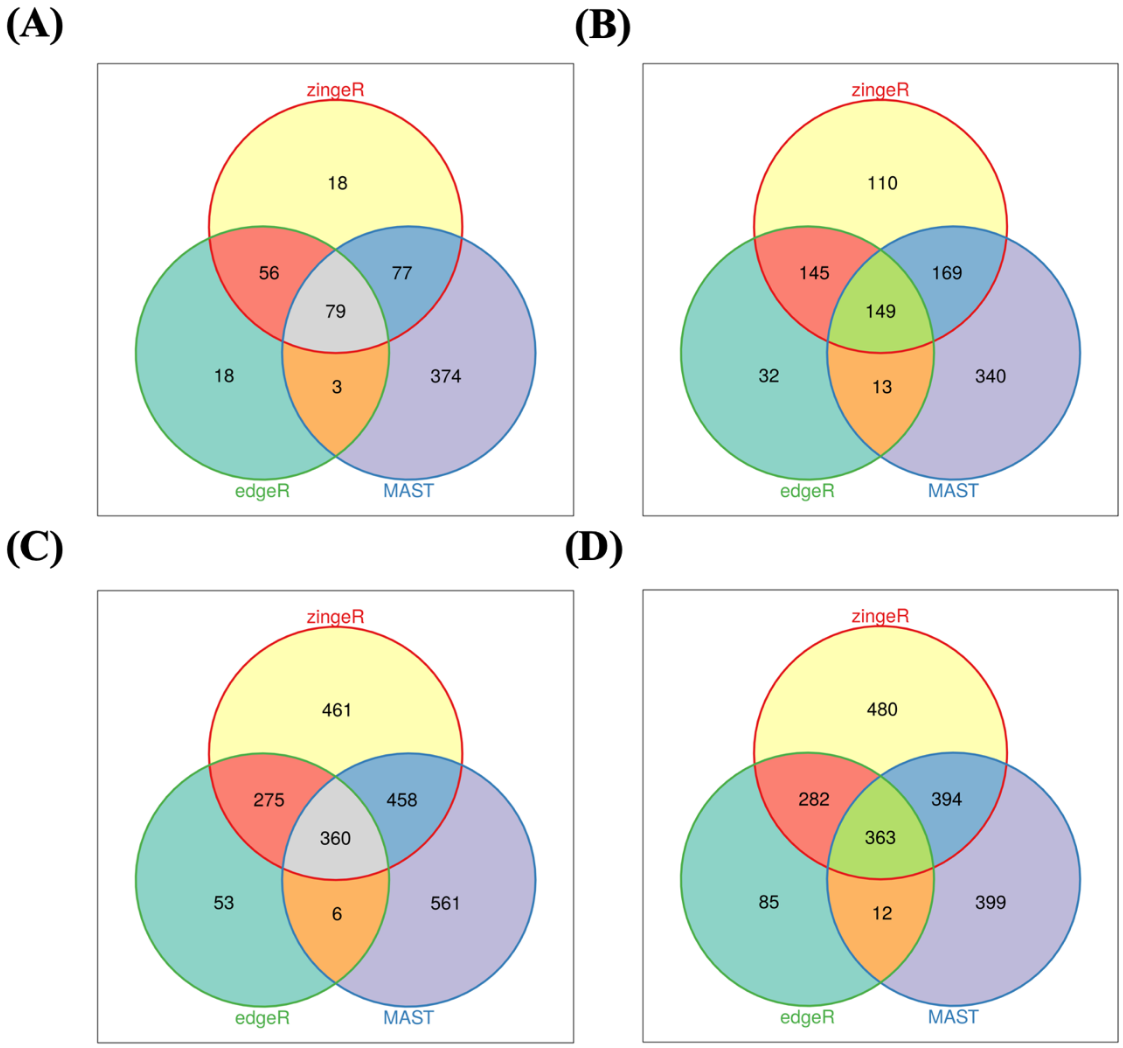

Supplementary Figure 21. iDEA displays high consistency in detecting DE genes in mouse neuronal cell scRNA-seq data. Overlap in top DE genes at an FDR of 0.1% between zingeR, MAST and edgeR (**A**); Overlap in top DE genes at an FDR of 0.1% between iDEA when using summary statistics from zingeR, MAST and edgeR respectively (**B**); Overlap in top DE genes at an FDR of 5% between zingeR, MAST and edgeR (**C**); Overlap in top DE genes at an FDR of 5% between iDEA when using summary statistics from zingeR, MAST and edgeR respectively (**D**);

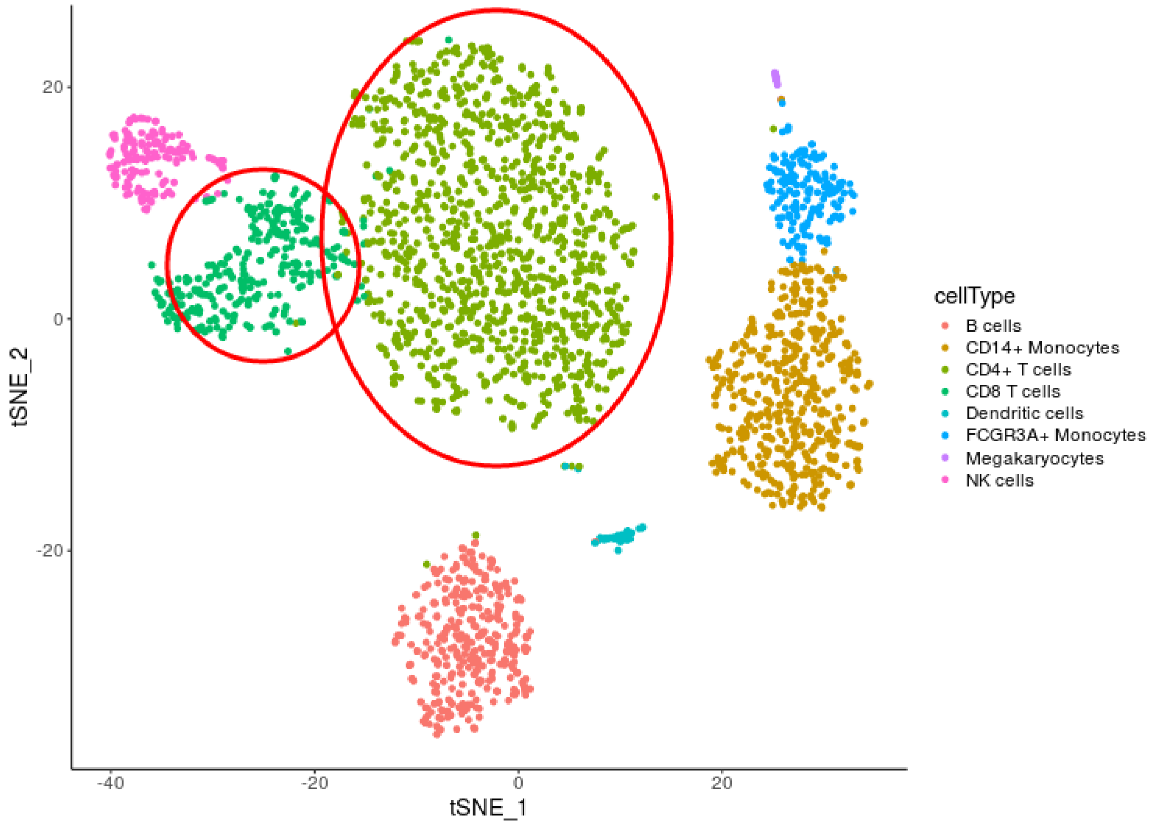

Supplementary Figure 22. The scatterplot of first two t-SNE principal components for 10x Genomics data set. There are total 8 cell types. CD4+ T cell type and CD8+ T cell type are highlighted in red circles. The cells are colored by Seurat clustering method.

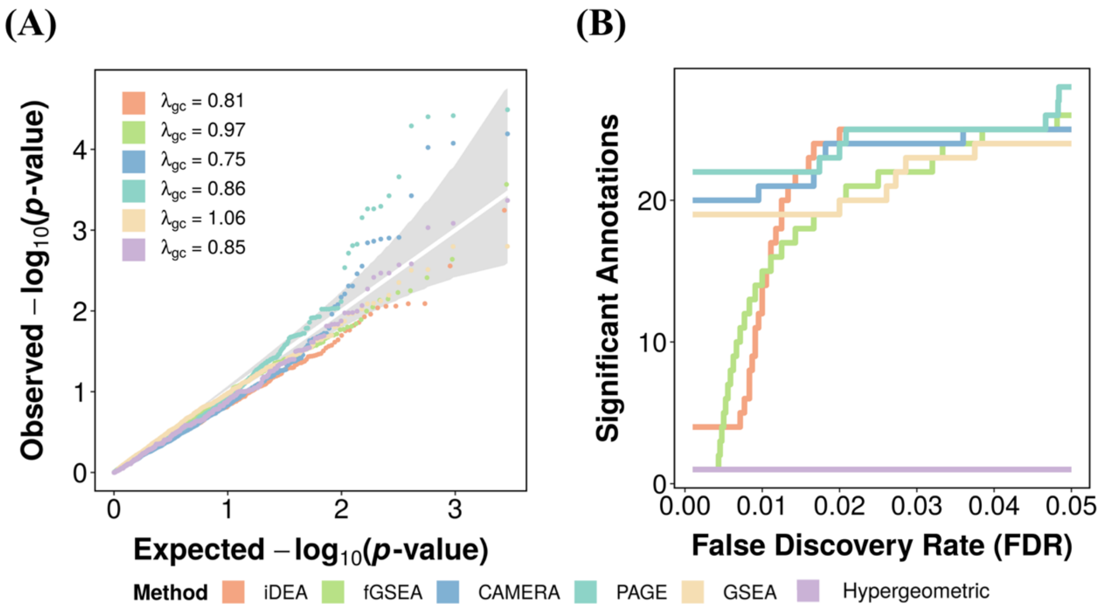

Supplementary Figure 23. GSE Analysis including hypergeometric test results in 10x Genomics data set. Results are shown for comparing CD4+ T cells versus CD8+ T cells. Quantile-quantile plots of -log10(*p*-values) from GSE methods including iDEA (orange), fGSEA (green), CAMERA (navyblue), PAGE (skyblue), GSEA (yellow) and Hypergeometric test (purple) are shown under permuted null (**A**). Number of identified enriched gene sets by iDEA (orange), fGSEA (green), CAMERA (navyblue), PAGE (skyblue) GSEA (yellow) and Hypergeometric test (purple) are plotted against different empirical false discovery rates (FDR) (**B**). Here λ_{gc} is the genomic control factor.

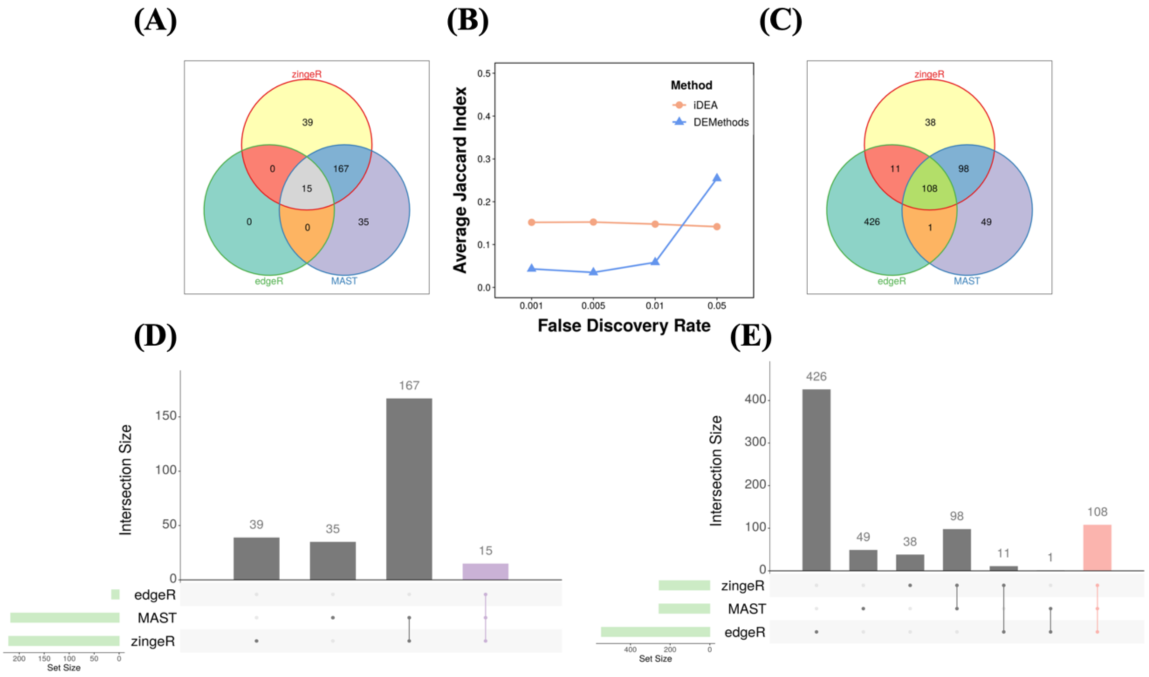

Supplementary Figure 24. iDEA displays high consistency in detecting DE genes in 10x Genomics scRNA-seq data. iDEA displays higher Jaccard index in the common DE genes. Jaccard index for top DE genes at an FDR of 1% between zingeR, MAST and edgeR, Jaccard index for top DE genes at an FDR of 1% between iDEA when using summary statistics from zingeR, MAST and edgeR respectively(red) (**B**); Overlap in top DE genes at an FDR of 1% between zingeR, MAST and edgeR **(A**); Overlap top DE genes at an FDR of 1% between iDEA when using summary statistics from zingeR, MAST and edgeR (**C**); **(D)** and **(E)** are just another visualization of the overlap corresponding to **(A)** and **(C)** by UpSetR[1].

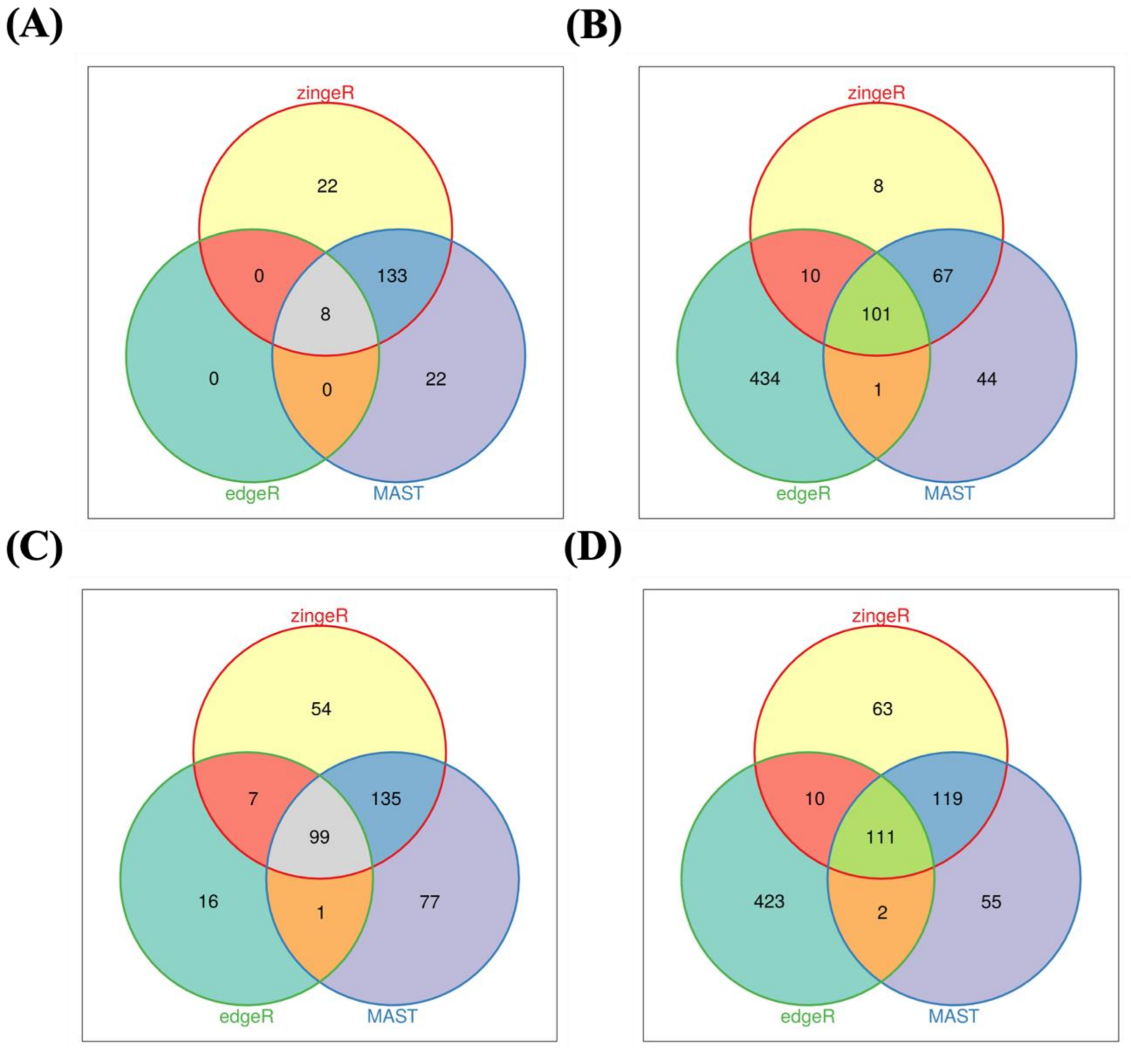

Supplementary Figure 25. iDEA displays high consistency in detecting DE genes in 10x Genomics scRNA-seq data. Overlap in top DE genes at an FDR of 0.1% between zingeR, MAST and edgeR (**A**); Overlap in top DE genes at an FDR of 0.1% between iDEA when using summary statistics from zingeR, MAST and edgeR respectively (**B**); Overlap in top DE genes at an FDR of 5% between zingeR, MAST and edgeR (**C**); Overlap in top DE genes at an FDR of 5% between iDEA when using summary statistics from zingeR, MAST and edgeR respectively (**D**);

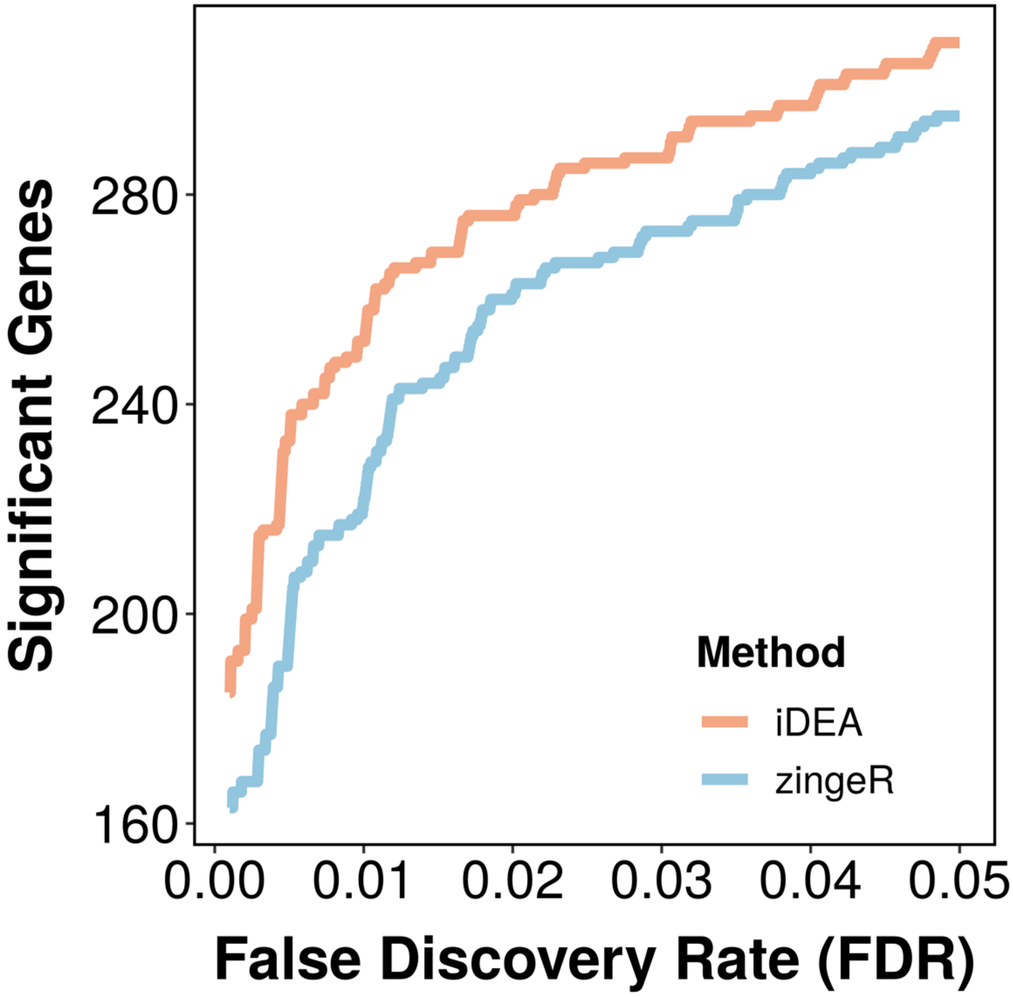

Supplementary Figure 26. DE analysis results in the 10X Genomics scRNA-seq data when no interested gene set information provided. Results are shown for comparing CD4+ T cells versus CD8+ T cells. Number of identified DE genes by iDEA (orange) and zingeR (blue) are plotted against different empirical FDR values. iDEA is more powerful than zingeR for DE analysis when there is no interested gene set information provided.

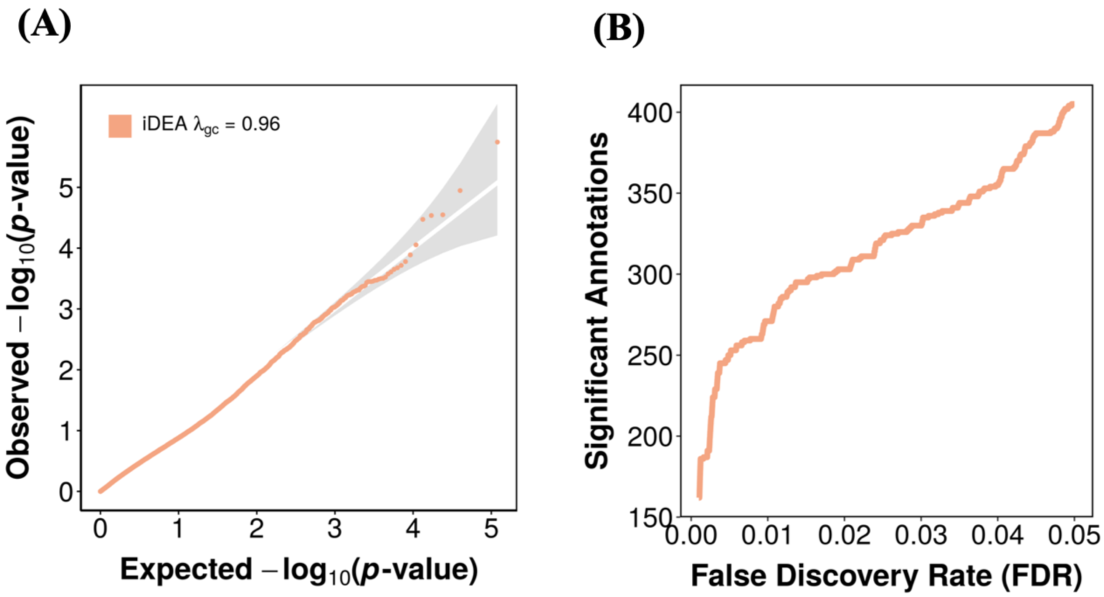

Supplementary Figure 27. iDEA analysis results in the 10X Genomics scRNA-seq data by using larger human gene sets. Results are shown for comparing CD4+ T cells versus CD8+ T cells. Quantile-quantile plots of -log10(*p*-values) from GSE methods including iDEA (orange) (**A**); Number of identified enriched gene sets by iDEA (orange) at a wide range of FDR (**B**); Here $\lambda_{\rm gc}$ is the genomic control factor.

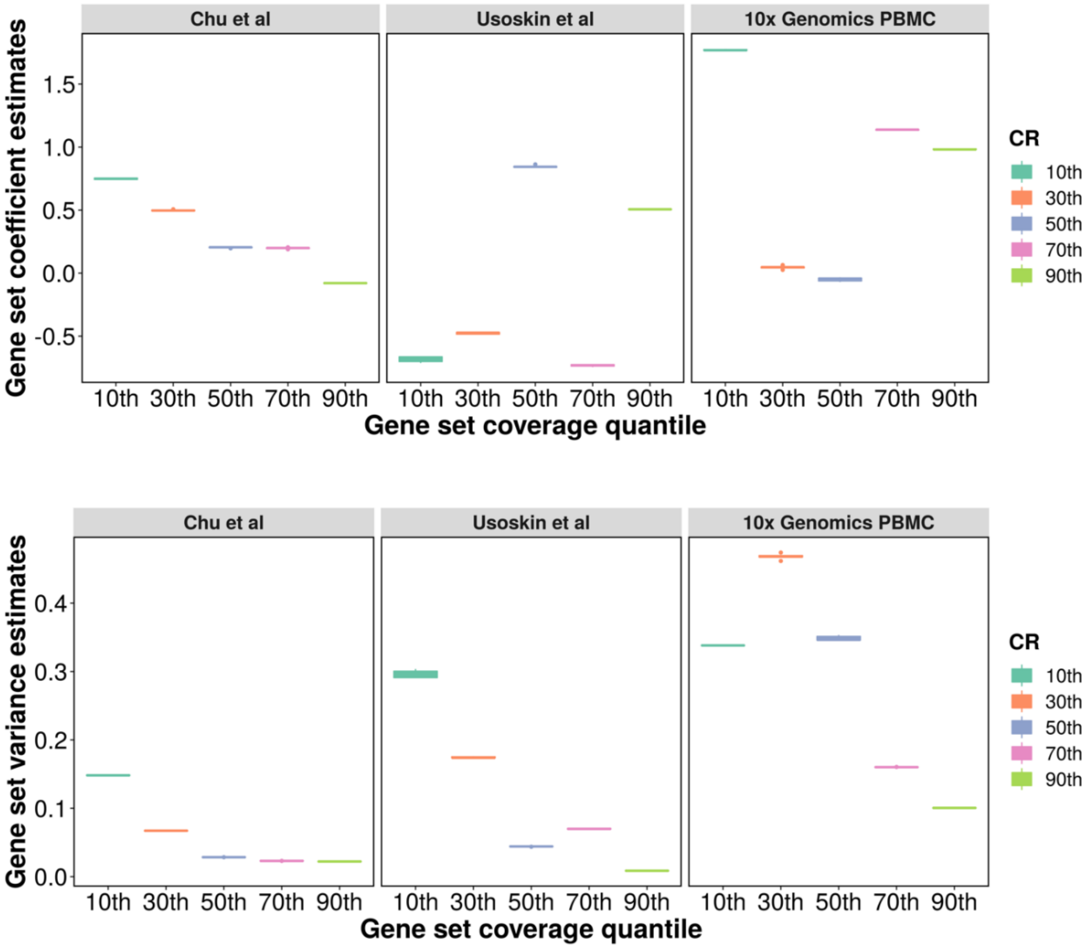

Supplementary Figure 28. Sensitivity analysis of hyperparameters in prior distribution of $\boldsymbol{\sigma_{\beta}^{2}}$. Boxplot of the estimates of gene set coefficient and variance of gene set coefficient are displayed for three scRNA-seq dataset: human embryonic stem cell (Chu et al), mouse neuronal cell (Usoskin et al) and 10x Genomics PBMC scRNA-seq dataset. For each dataset, we tested the parameter estimates on gene sets with different coverage rate percentile among all gene sets we analyzed in that corresponding dataset. For each gene set with different coverage rate, estimates of gene set coefficient and variance were obtained under different prior distribution of σ_{β}^{2}. Parameter estimates are stable for gene sets across a wide range of prior distribution of σ_{β}^{2}. For each box plot, the bottom and the top of the box are the 25th and 75th quantiles, while the whiskers represent 1.5 * interquartile range from the lower and upper bounds of the box.

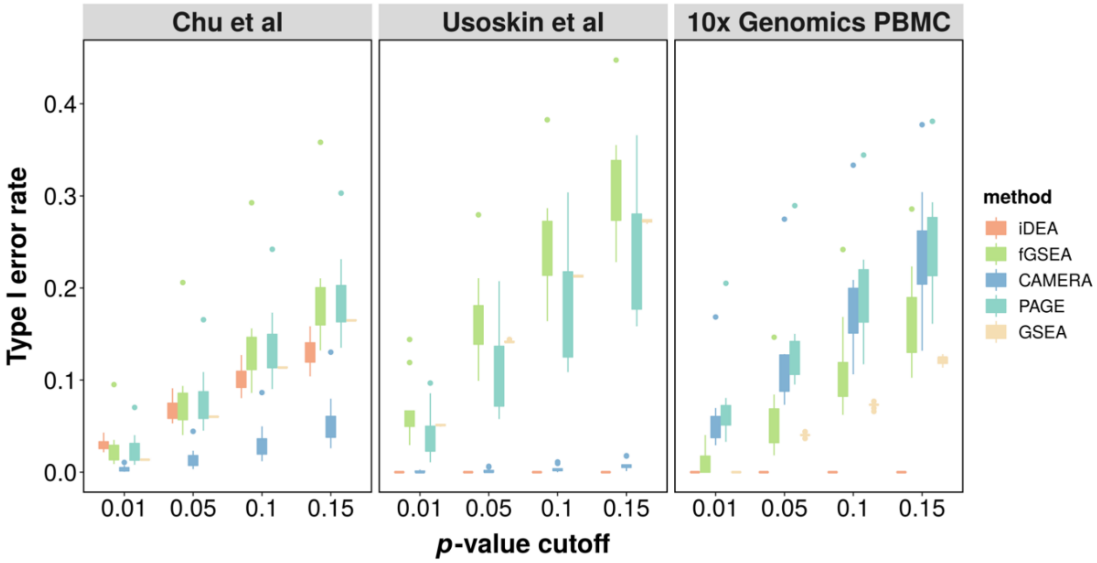

Supplementary Figure 29. Type I error rate in real datasets. We splited the dataset within the same cell type (n = 10 replicates) to construct the true null distribution**.** Box plot of Type I error rate of iDEA (orange), fGSEA (green), CAMERA (navyblue), PAGE (skyblue) and GSEA (yellow) are shown in human embryonic stem cell scRNA-seq dataset (Chu et al), mouse neuronal cell scRNA-seq dataset (Usoskin et al) and 10x Genomics PBMC scRNA-seq dataset. iDEA controlled type I error well in all three data sets. For each box plot, the bottom and the top of the box are the 25th and 75th quantiles, while the whiskers represent 1.5 * interquartile range from the lower and upper bounds of the box.

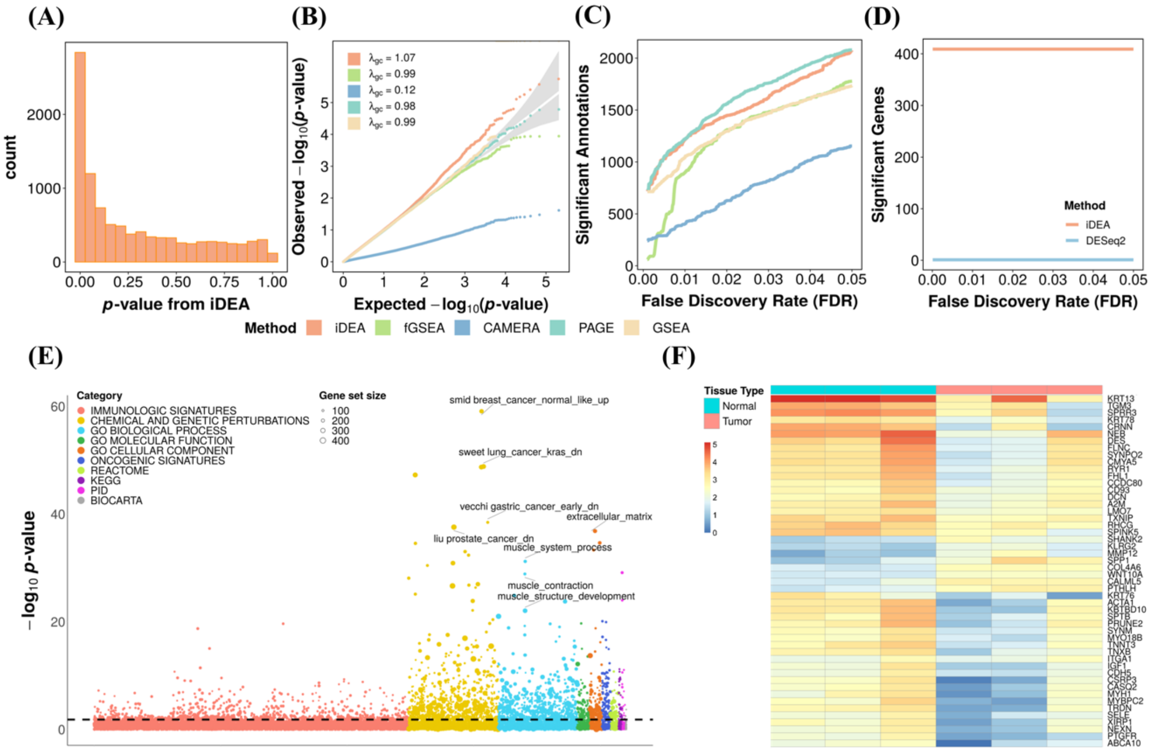

Supplementary Figure 30. Analysis results in the bulk RNAseq data. Results are shown for comparing matched normal oral tissue versus oral squamous cell carcinoma. (**A**) *p*-values from iDEA for GSE analysis display expected enrichment of small *p*-values (for true signals) and a long flat tail towards large *p*-values. (**B**) Quantile-quantile plots of -log10(*p*-values) from GSE methods including iDEA (orange), fGSEA (green), CAMERA (navyblue), PAGE (skyblue) and GSEA (yellow) are shown under permuted null. The *p*-values from iDEA, fGSEA, PAGE and GSEA are reasonably well calibrated. The *p*-values from CAMERA are overly conservative. Here λ_{gc} is the genomic control factor. (**C**) Number of identified enriched gene sets by iDEA (orange), fGSEA (green), CAMERA (navyblue), PAGE (skyblue) and GSEA (yellow) are plotted against different empirical false discovery rates (FDR). iDEA is as the same powerful as PAGE than other methods for GSE analysis. (**D**) Number of identified DE genes by iDEA (orange) and DESeq2 (blue) are plotted against different empirical FDR values. iDEA is more powerful than DESeq for DE analysis. (**E**) Heatmap shows the normalized expression level (log10-transformation with pseudo-count 0.1) for selected 50 DE genes (rows) identified by iDEA for cells in the two tissue types (columns). Genes are sorted by Hierarchical clustering; cells are ordered by tissue types (Normal: blue; Tumor: red). These DE genes clearly distinguish two compared tissues. (**F**) Bubble plot shows –log10 *p*-values for GSE analysis from iDEA (y-axis) for different gene sets. Gene sets are colored by ten categories: immunologic signatures (red), chemical and genetic perturbations (yellow), GO biological process (blue), GO molecular function (green), GO cellular component (orange), oncogenic signatures (deep blue), Reactome (grass-green), KEGG (purple), PID (rose), and Biocarta (grey). The size of the dot represents the number of genes contained in the gene set. Names for ten of the gene sets that are closely related to oral squamous cell carcinoma are highlighted in the panel.

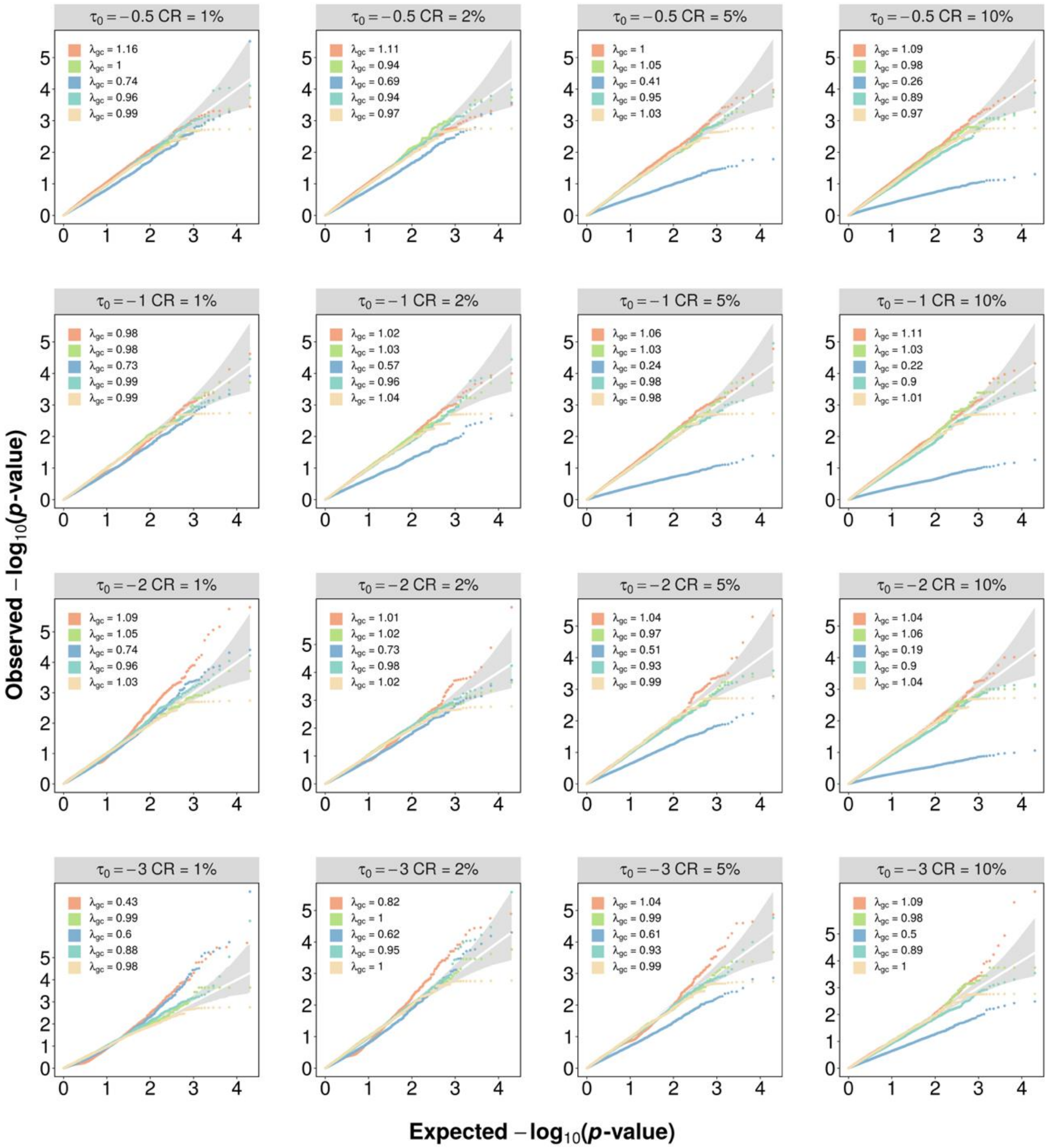

Supplementary Figure 31. iDEA produces well-calibrated *p*-values for gene set enrichment analysis under null simulations in iDEA variant when modeling beta effect size directly. Quantile-quantile plots of -log10(*p*-values) from iDEA (orange), fGSEA (green), CAMERA (navyblue), PAGE (skyblue) and GSEA (yellow) are shown under different null scenarios with varying number of DE genes (denoted by the odd parameter τ_0; -0.5, -1.0, -2.0, or -3.0) and gene set coverage rates (CR; 1%, 2%, 5% or 10%). CR represents the percentage of genes inside the gene set. λ_{gc} is genomic control factor.

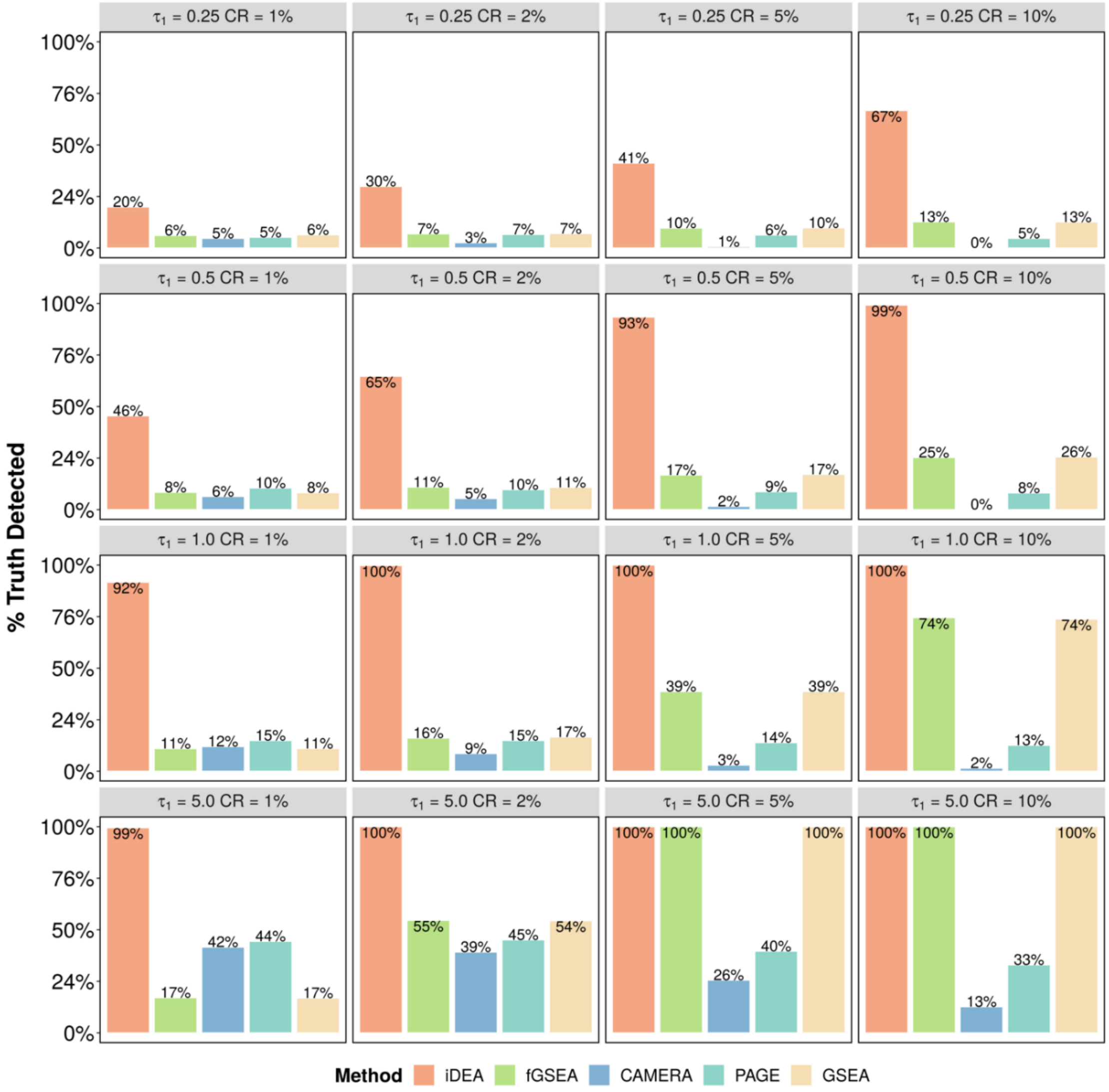

Supplementary Figure 32. iDEA is more powerful than GSE methods for identifying enriched gene sets under alternative simulations in iDEA variant when modeling beta effect size directly. The power plots from iDEA (orange), fGSEA (green), CAMERA (navyblue), PAGE (skyblue) and GSEA (yellow) are shown under different scenarios with varying gene set enrichment coefficient (denoted by the odd parameter τ_1; 0.25, 0.5, 1.0 or 5.0) and gene set coverage rates (CR; 1%, 2%, 5% or 10%). CR represents the percentage of genes inside the gene set. Here, power was calculated based on an FDR of 5%.

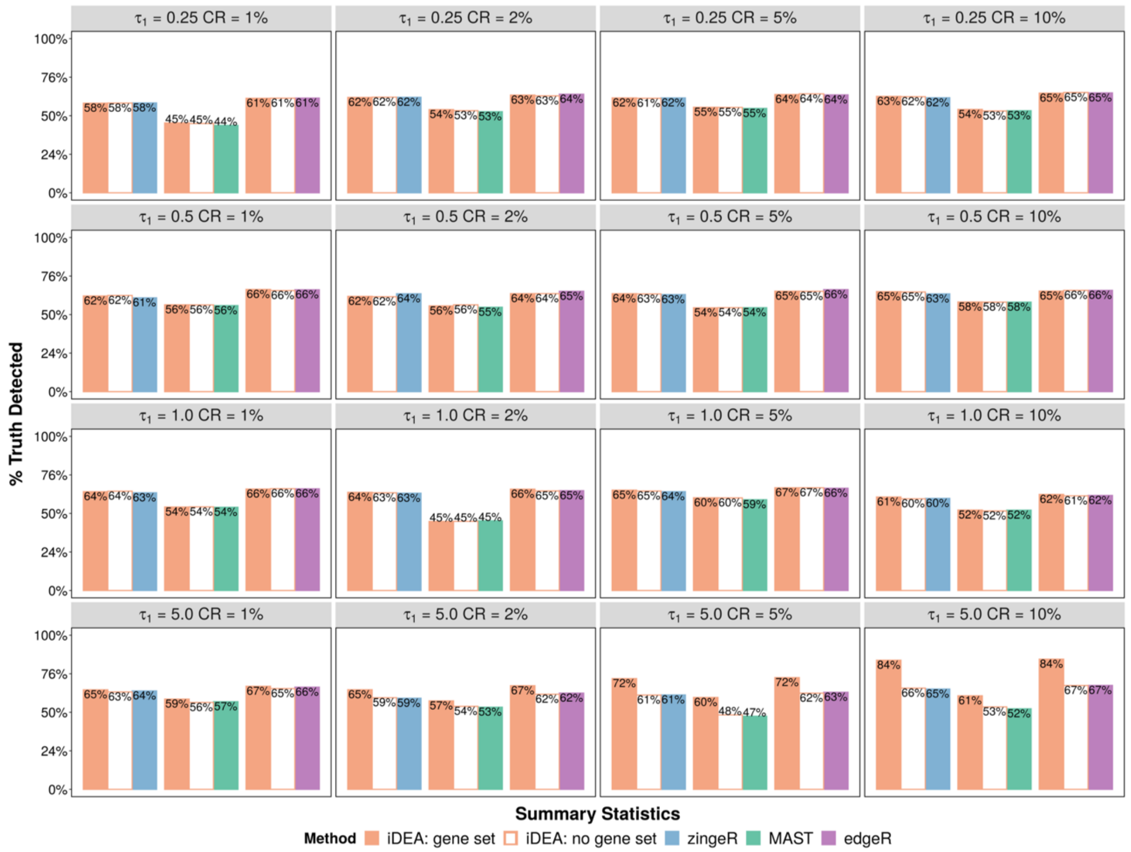

Supplementary Figure 33. iDEA is more powerful than DE methods for identifying DE genes under alternative simulations in iDEA variant when gene set enrichment parameter is larger. Simulations were performed on one fixed scRNA-seq data set with $\tau_0 = -2$, varying τ_1 and CR. τ_1 is set to be 0.25, 0.5,1.0 or 5.0 and CR is set to be 1%, 2%, 5%, 10% respectively. In each simulation setting, power of DE results between common DE method (zingeR (blue), MAST (green), edgeR (purple)) and iDEA (orange) with summary statistics obtained from that corresponding DE method when adding simulated gene set (filling color) or not (not filling color) is plotted. The power was calculated as the percentage of truly DE genes detected in each method. Here, power was calculated based on an FDR of 5%.

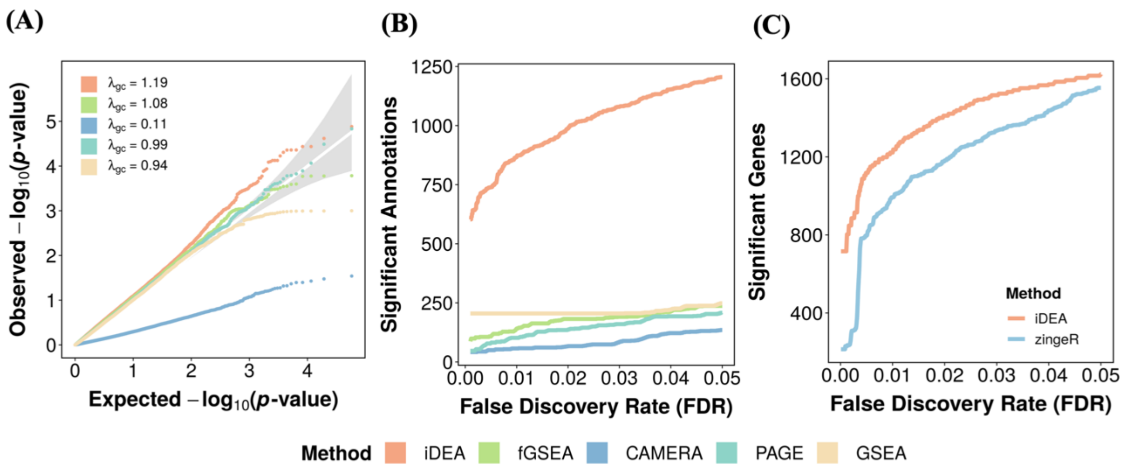

Supplementary Figure 34. Analysis results of iDEA variant when modeling on beta effect size directly in the mouse neuronal cell scRNA-seq data. Results are shown for comparing nonpeptidergic nociceptors 1 (NP1) versus all the other cell types. (**A**) Quantile-quantile plots of -log10(*p*-values) from GSE methods including iDEA (orange), fGSEA (green), CAMERA (navyblue), PAGE (skyblue) and GSEA (yellow) are shown under permuted null. The *p*-values from iDEA and fGSEA are reasonably well calibrated. The *p*-values from CAMERA are overly conservative. Here λ_{gc} is the genomic control factor. (**B**) Number of identified enriched gene sets by iDEA (orange), fGSEA (green), CAMERA (navyblue), PAGE (skyblue) and GSEA (yellow) are plotted against different empirical false discovery rates (FDR). iDEA is more powerful than other methods for GSE analysis. (**C**) Number of identified DE genes by iDEA (orange) and zingeR (blue) are plotted against different empirical FDR values. iDEA is more powerful than zingeR for DE analysis.

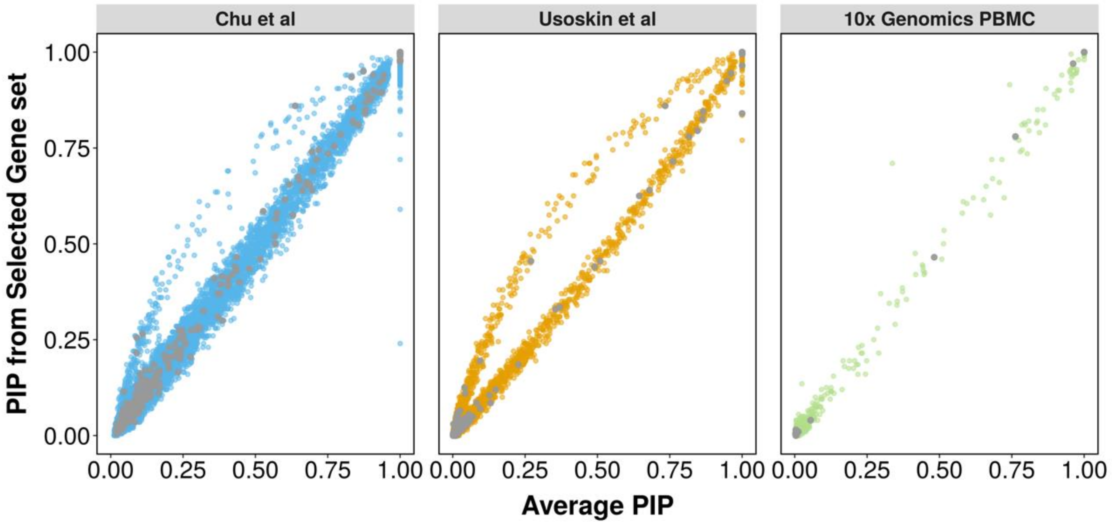

Supplementary Figure 35. Posterior inclusion probabilities (PIPs) calculated by iDEA when adding specific gene set is highly correlated with averaging PIPs across all gene sets in all three scRNA-seq datasets. Here, each dot represents each gene with x-axis represents the averaged pip and y-axis represents gene set specific pip. Genes in that selected gene set are highlighted by grey color. In Human embryonic stem cell scRNA-seq dataset (Chu et al), we added the gene set GO:0001944 (vasculature development). In Mouse Sensory neuron scRNA-seq dataset, we added the gene set GO:0097458 (neuron part). In 10x Genomics PBMC dataset, we added the gene set CD8+ T-effector memory Term.

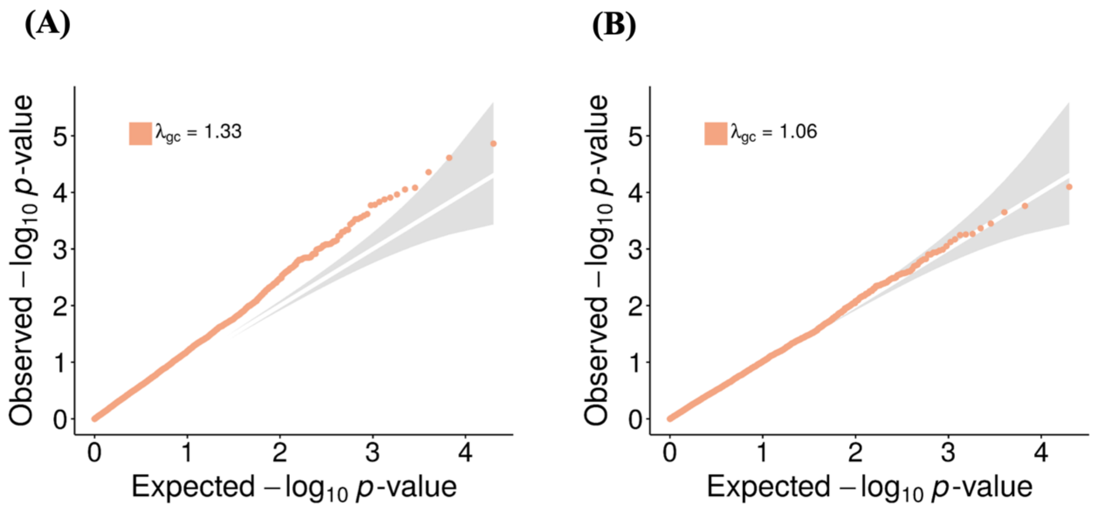

Supplementary Figure 36. iDEA produces calibrated *p*-values in scRNA-seq based null simulations when using Louis Method to correct the observed information matrix. Quantile-quantile plots of -log10(*p*-values) are shown for: iDEA without Louis Method (**A**); iDEA with Louis method **(B);** respectively under the null that simulated one fixed scRNA-seq data set and permute the gene set 10,000 times. Here, the other parameters are set to be $\tau_0 = -2, \tau_1 = 0$ and $\mathrm{CR} = 10\%$. CR represents the percentage of genes inside the gene set. λ_{gc} is genomic control factor.

Supplementary Notes

Supplementary Note 1. EM-MCMC Inference Algorithm

The iDEA model is described in detail in the Methods. Here, we describe the detailed algorithm for inference. As explained in the main text, our goal is to infer the posterior probability of $\gamma_j = 1$ as evidence for *j*-th gene being DE and test the null hypothesis $H_0\colon \tau_1 = 0$ that DE genes are not enriched in the gene set. To achieve both goals, we develop an efficient expectation maximization (EM)-Markov chain Monte Carlo (MCMC) algorithm. To simplify notation**,** we denote $\boldsymbol{\beta}$ as the *p*-vector of the underlying true effect sizes, or $\boldsymbol{\beta} = (\beta_1, \beta_2, \cdots, \beta_p)^T$. We denote $\boldsymbol{\gamma}$ as the *p*-vector of the indicator variables, or $\boldsymbol{\gamma} = (\gamma_1, \gamma_2, \cdots, \gamma_p)^T$. We denote $\sigma_{e_j}^2$ as the variance of the marginal DE effect size estimate for *j*-th gene, or $\sigma_{e_j}^2 = \mathrm{se}^2(\hat{\beta}_j)$. We treat both $\boldsymbol{\beta}$ and $\boldsymbol{\gamma}$ as missing data and write out the complete likelihood as

$$\begin{aligned}
\log\Pr(\widehat{\boldsymbol{\beta}}, \boldsymbol{\beta}, \boldsymbol{\gamma} | \tau_0, \tau_1, \sigma_\beta^2) &= \log\{\Pr(\widehat{\boldsymbol{\beta}} | \boldsymbol{\beta}, \boldsymbol{\gamma}) \Pr(\boldsymbol{\beta} | \boldsymbol{\gamma}, \sigma_\beta^2) \Pr(\boldsymbol{\gamma} | \tau_0, \tau_1) \Pr(\sigma_\beta^2 | a_{\beta,} b_\beta)\} \\
&= -\frac{1}{2}\sum_{j=1}^{p} \gamma_j \left(\log\left(\sigma_{e_j}^2\right) + \frac{(\widehat{\beta}_j - \beta_j)^2}{\sigma_{e_j}^2}\right) \\
&\quad -\frac{1}{2}\sum_{j=1}^{p} \gamma_j \left(\log\left(\sigma_{e_j}^2 \sigma_\beta^2\right) + \frac{\beta_j^2}{\sigma_{e_j}^2 \sigma_\beta^2}\right) \\
&\quad + \sum_{j=1}^{p} \gamma_j \log(\pi_j) + (1 - \gamma_j)\log(1 - \pi_j) \\
&\quad - (a_\beta + 1)\log(\sigma_\beta^2) - b_\beta \sigma_\beta^{-2}, \qquad (1)
\end{aligned}$$

where we have also ignored the constant terms in the above equation and $\pi_j = \frac{\exp(\tau_0 + a_j\tau_1)}{1 + \exp(\tau_0 + a_j\tau_1)}$. With the above complete likelihood, we can derive the expectation step (E-Step) and maximization step (M-Step) as follows.

Expectation Step (E-Step)

In the E-Step, we obtain the expectation of equation (1)

$$Q = E\left[\log\Pr(\widehat{\boldsymbol{\beta}}, \boldsymbol{\beta}, \boldsymbol{\Gamma} | \boldsymbol{\tau}, \sigma_\beta^2)\right], \qquad (2)$$

which involves evaluating the expectations $E(\gamma_j), E(\gamma_j\beta_j)$ and $E(\gamma_j\beta_j^2)$. These expectations are obtained under the conditional distributions $\mathrm{P}(\beta_j, \gamma_j | \widehat{\boldsymbol{\beta}}, \tau_0^{(t)}, \tau_1^{(t)}, (\sigma^{(t)})_\beta^2)$, with $\tau_0^{(t)}, \tau_1^{(t)}$ and $(\sigma^{(t)})_\beta^2$ being the estimates from the

previous iteration *t*. These conditional distributions are unfortunately not available in analytic forms. Therefore, we use Markov Chain Monte Carlo (MCMC) to obtain these expectations. Specifically, we develop a Gibbs sampling to sample the posterior distributions for β_j and γ_j in an alternate fashion. Afterwards, we use these posterior samples to evaluate the above expectations. To do so, we first integrate out β_j from the complete likelihood and obtain the conditional distribution for γ_j as

$$\Pr\left(\gamma_j = 1 \middle| \widehat{\boldsymbol{\beta}}, \tau_0^{(t)}, \tau_1^{(t)}, \left(\sigma^{(t)}\right)_\beta^2\right) \propto \exp\left\{\frac{m_j^2}{2s_j^2} + \log(s_j) - \log\left(\sigma_\beta^{(t)}\right) + \log\left(\pi_j^{(t)}\right)\right\}, \quad (3)$$

$$\Pr\left(\gamma_j = 0 \middle| \widehat{\boldsymbol{\beta}}, \tau_0^{(t)}, \tau_1^{(t)}\right) \propto 1 - \pi_j^{(t)}. \quad (4)$$

Then posterior distribution of γ_j is,

$$\gamma_j \sim \text{Bernoulli}\left(\frac{\Pr\left(\gamma_j = 1 \middle| \widehat{\boldsymbol{\beta}}, \tau_0^{(t)}, \tau_1^{(t)}, \left(\sigma^{(t)}\right)_\beta^2\right)}{\Pr\left(\gamma_j = 1 \middle| \widehat{\boldsymbol{\beta}}, \tau_0^{(t)}, \tau_1^{(t)}, \left(\sigma^{(t)}\right)_\beta^2\right) + \Pr\left(\gamma_j = 0 \middle| \widehat{\boldsymbol{\beta}}, \tau_0^{(t)}, \tau_1^{(t)}, \left(\sigma^{(t)}\right)_\beta^2\right)}\right) \quad (5)$$

where $m_j = \frac{\widehat{\beta}_j}{1+\left(\sigma_\beta^{(t)}\right)^{-2}}$ and $s_j^2 = \frac{\sigma_{e_j}^2}{1+\left(\sigma_\beta^{(t)}\right)^{-2}}$. Next, we recognize from the complete likelihood that the conditional distribution of β_j given $\gamma_j = 1$ is normal:

$$\beta_j | \gamma_j = 1 \sim N\left(m_j, s_j^2\right). \quad (6)$$

Certainly, $\beta_j = 0$ if $\gamma_j = 0$.

Maximization Step (M-Step)

In the M-Step, we obtain the parameter estimates for τ_0, τ_1 and σ_β^2 that maximize the Q function obtained in the E-Step. For τ_0 and τ_1, we obtain the first derivatives of the Q function with respect to each parameter as

$$\frac{\partial Q}{\partial \tau_0} = \sum_{j=1}^{p} \left(E(\gamma_j) - \pi_j\right),$$

$$\frac{\partial Q}{\partial \tau_1} = \sum_{j=1}^{p} a_j\left(E(\gamma_j) - \pi_j\right). \quad (7)$$

We also obtain the second derivatives as

$$\frac{\partial^2 Q}{\partial \tau_0^2} = \sum_{j=1}^{p} \pi_j\left(1 - \pi_j\right),$$

$$\frac{\partial^2 Q}{\partial\tau_0\partial\tau_1} = \sum_{j=1}^{p} a_j\pi_j(1-\pi_j),$$

$$\frac{\partial^2 Q}{\partial\tau_1^2} = \sum_{j=1}^{p} a_j^2\pi_j(1-\pi_j). \quad (8)$$

where π_j is calculated as the expectation of the indicator variable γ_j $\mathrm{E}\left(\gamma_j \middle| \widehat{\boldsymbol{\beta}}, \tau_0^{(t)}, \tau_1^{(t)}, \left(\sigma^{(t)}\right)_\beta^2\right)$. And π_j is used in the following Newton-Raphson algorithm to obtain the parameter estimate of the intercept τ_0 and gene set coefficient τ_1. Afterwards, we use the Newton-Raphson algorithm for optimization and obtain estimates of $\tau_0^{(t+1)}$ and $\tau_1^{(t+1)}$.

For σ_β^2, we obtain the first derivatives of the Q function with respect to σ_β^2 as

$$\frac{\partial Q}{\partial\sigma_\beta^2} = \sigma_\beta^{-4}\left(\sum_{j=1}^{p}\frac{E(\gamma_j\beta_j^2)}{2\sigma_{e_j}^2} + b_\beta\right) - \sigma_\beta^{-2}\left(\frac{\sum_{j=1}^{p} E(\gamma_j)}{2} + a_\beta + 1\right),$$

which leads to an analytical update for σ_β^2 as

$$\left(\sigma_\beta^{(t+1)}\right)^2 = \frac{\sum_{j=1}^{p}\frac{E(\gamma_j\beta_j^2)}{2\sigma_{e_j}^2} + b_\beta}{\frac{\sum_{j=1}^{p} E(\gamma_j)}{2} + a_\beta + 1}. \quad (9)$$

The EM-MCMC algorithm thus iterates between the E-step and the M-step until converge. The EM-MCMC algorithm allows us to directly obtain the parameter estimate $E(\gamma_j)$, which is the posterior probability of *j*-th gene being a DE gene. This posterior probability is also commonly referred to as the posterior inclusion probability (PIP) in other settings. We use these posterior probabilities to serve as DE evidence. In addition, the EM-MCMC algorithm also provides an estimate for τ_1, which, when paired with its standard error computed in the following section, allows us to construct a Wald test to test the null hypothesis of no gene set enrichment $H_0: \tau_1 = 0$.

Supplementary Note 2. Louis Method for *p*-value Computation

Here, we describe the details of the Louis method for computing the standard error of $\hat{\tau}_1$. In the EM-MCMC algorithm described in the previous section, we can obtain the information matrix for (τ_0, τ_1) based on the log complete likelihood $\log\Pr(\widehat{\boldsymbol{\beta}}, \boldsymbol{\beta}, \boldsymbol{\gamma}|\tau_0, \tau_1, \sigma_\beta^2)$ as described in equation (1). For completeness, we re-write the information matrix in the complete likelihood as a 2 by 2 matrix

$$I_c = \sum_{j=1}^{p} \hat{\pi}_j \left(1 - \hat{\pi}_j\right) \begin{pmatrix} 1 & a_j \\ a_j & a_j^2 \end{pmatrix}, \quad (10)$$

where $\hat{\pi}_j = \frac{\exp(A_j \hat{\boldsymbol{\tau}})}{1+\exp(A_j \hat{\boldsymbol{\tau}})}$ is computed based on the $\hat{\boldsymbol{\tau}}$ estimates from the last EM step and a_j is the annotation for j-th gene. Our goal, however, is to obtain the information matrix for (τ_0, τ_1) based on the marginal log likelihood $\log\Pr(\hat{\boldsymbol{\beta}}|\tau_0, \tau_1, \sigma_\beta^2)$, also known as the observed likelihood. Such marginal information matrix can be obtained based on the complete information matrix through an adjustment using the Louis method[2,3]. Specifically, with the posterior inclusion probably PIP_j for *j*-th gene obtained from EM steps, we compute the information matrix in the incomplete likelihood as a 2 by 2 matrix

$$I_{ic} = \sum_{j=1}^{p} \mathrm{PIP}_j \left(1 - \mathrm{PIP}_j\right) \begin{pmatrix} 1 & a_j \\ a_j & a_j^2 \end{pmatrix}. \quad (11)$$

Finally, the observed information matrix I_o is adjusted by

$$I_o = I_c - I_{ic}$$

Once we compute the marginal information matrix, we can obtain the standard error $\mathrm{se}^2(\hat{\tau}_1)$ as the corresponding element in the inverse of the information matrix I_o.

Supplementary Note 3. Application to an oral carcinoma bulk RNAseq dataset

To illustrate the flexibility of the modeling framework in iDEA, we applied iDEA to analyze a publicly available bulk RNASeq dataset from Tuch et al[4]. The bulk RNAseq dataset consists of gene expression measurements for 10,540 genes on tumors and matched normal tissue from three patients with oral squamous cell carcinomas. We carried out both GSE analyses and DE analyses on comparing the matched tumor and normal pairs.

We first applied iDEA and other GSE methods to detect significantly enriched gene sets across our compiled database of 12,033 human gene sets. The *p*-values of the enriched gene sets from iDEA are shown in Supplementary Figure 30A. We also constructed an empirical null *p*-value distribution by permuting the gene labels for each gene set 10 times. Consistent with both simulations and scRNA-seq data applications, we found that the *p*-values in the permuted data from iDEA ($\lambda_{\mathrm{gc}} = 1.07$), fGSEA ($\lambda_{\mathrm{gc}} = 0.99$), PAGE ($\lambda_{\mathrm{gc}} = 0.98$), and GSEA ($\lambda_{\mathrm{gc}} = 0.99$) are well behaved, while that from CAMERA show severe deflation ($\lambda_{\mathrm{gc}} =$

0.12)(Supplementary Figure 30B). For each method, we relied on the empirical null distribution of *p*-values to compute power in detecting enriched gene sets based on a fixed empirical FDR. Consistent with both simulations and scRNA-seq data applications, iDEA displays higher power compared to the other GSE methods (Supplementary Figure 30C). For example, at an empirical FDR of 5%, iDEA identified 2075 significantly enriched gene sets, which is 17%, 80%, 20% higher than fGSEA (1777), CAMERA (1154), and GSEA (1733) respectively. While PAGE (2079) also displays higher power in number of detecting the significant gene sets, the top gene sets identified by iDEA are most closely related to oral squamous cell carcinomas or tumor related pathways. For example, among the top 10 gene sets identified by iDEA, 7 are related to tumor pathways. As a comparison, 5 among the top 10 gene sets identified by PAGE are related to tumor pathways. Specifically, enriched gene sets identified by iDEA include the SMID_BREAST_CANCER_NORMAL_LIKE_UP[5],SWEET_LUNG_CANER_KRAS_DN[6] and relevant GO items such as GO:0031012 (extracellular matrix[7]), GO:0043292 (contractile fiber[8]). In order to quantify the biological significance of gene sets identified by different GSE methods, we quantified the relevance between gene sets and oral squamous cell carcinomas in an unbiased way by searching the related literatures in PubMed (details in Materials and Methods). Indeed, in the top 50 enriched gene sets identified by different methods, iDEA identified more gene sets relevant to oral squamous cell carcinomas (30) than fGSEA (23), CAMERA (30), PAGE (23), and GSEA (25). The higher number of detected enriched gene sets relevant to oral squamous cell carcinomas and cancer growth by iDEA provides convergent support for the higher power of iDEA for GSE analysis.

Next, we applied iDEA for DE analysis to identify DE genes. We obtained the summary statistics from DESeq2. Consistent with both simulations and scRNA-seq data applications, iDEA identified more DE genes than zingeR. For example, at an empirical FDR of 1%, iDEA identified 409 DE genes, while DESeq2 only identified 1 (Supplementary Figure 30D). The 50 selected important DE genes identified by iDEA clearly distinguishes the normal tissue and cancer tissue (Supplementary Figure 30F). Importantly, using the key markers provided by the original study[9], iDEA identified 262 genes directly related to oral squamous cell carcinomas or important genes involved in common tumors; while DESeq2 only identified 1. The higher number of DE genes relevant to oral squamous cell carcinomas or common tumors detected by iDEA provides convergent support for its higher power for DE analysis. Important DE genes involved in Oral squamous

cell carcinoma development that are detected by iDEA but missed by DESeq2 include *CRNN*[10], *WNT10A*[11], *PTHLH*[12], *KRT6*[13], *IGF1*[14], *PTGFR*[15], *TGFBR3*[16]. Among them, *CRNN* has been studied to be the potential prognostic marker of OSCC due to its downregulation in oral squamous cell carcinoma samples, *WNT10A* plays an important role in accelerating of the progression of carcinomas via activating EMTs and local invasiveness[11], *PTHLH* is indispensable for the pathogenesis of oral squamous cell carcinoma by affecting cell proliferation and cell cycle[12]. *TGFBR3* is an important activator of *GDF10*, which is downregulated during oral carcinogenesis and involved in the suppression of cell survival[16].

Further, we also evaluated the GC content and gene length effect. For all the genes in the dataset, we first calculated the GC content and gene length and then we create two gene sets corresponding to the levels of GC content and gene length. Specifically, for the GC content, we use the continuous value of GC content for each gene as the gene set. For the gene length, we created a binary gene set if gene length is higher than the average of the gene length, than this gene is in this gene set and annotated as 1 otherwise 0. Finally, by adding these two gene sets into iDEA, we calculated the *p*-values for these two gene set to represent the significance of GC content effect and gene length effect correspondingly. In the analyses, we did not observe obvious GC content effect (*p*-value = 0.31) and gene length effect (*p*-value = 0.08) in this dataset.

Supplementary Note 4. iDEA variant

Model and statistical inference

As explained in the Discussion, the iDEA model makes an implicit assumption that the prior depends on the sample size. Here, we provide an iDEA variant with alternative modeling assumption that does not have the prior dependence on the sample size. Here, the input summary statistics are again in the form of marginal DE effect size estimate $\hat{\beta}_j$ and its standard error $\mathrm{se}(\hat{\beta}_j),\ j = 1,2,\cdots p$, where p is the number of genes. We assume that the true effect size β_j follows a mixture of two distributions depending on whether j-th gene is a DE gene or not:

$$\hat{\beta}_j = \beta_j + \epsilon_j,\ \epsilon_j \sim N(0, \mathrm{se}(\hat{\beta}_j)^2) \tag{12}$$

$$\beta_j \sim \pi_j\, N(0, \sigma_\beta^2) + (1 - \pi_j)\delta_0 \tag{13}$$

where π_j is the prior probability of being a DE gene; σ_β^2 is a scaling factor that determines the DE effect size strength; and δ_0 is the Dirac function that represents a point mass at zero. Therefore, with proportion π_j, *j*-th gene is a DE gene and its

DE effect size β_j follows a normal distribution with a large variance σ^2_β. With proportion $1-\pi_j$, *j*-th gene is a non-DE gene and its DE effect size is exactly zero

Note that, compared to the original iDEA model, we have removed $se(\hat{\beta}_j)^2$ and only maintain the σ^2_β as the variance in the prior distribution of true effect size. In this way, the effect size β_j is no longer depend on the standard error $se(\hat{\beta}_j)$ and thus the sample size. For the rest of the algorithm and methodology, we just followed the same procedure as the original model. Here, we mainly report the modified parts in the EM algorithm when using this model. The complete likelihood changed to be the following

$$\log\Pr(\widehat{\boldsymbol{\beta}},\boldsymbol{\beta},\boldsymbol{\gamma}|\tau_0,\tau_1,\sigma^2_\beta) = \log\{\Pr(\widehat{\boldsymbol{\beta}}|\boldsymbol{\beta},\boldsymbol{\gamma})\Pr(\boldsymbol{\beta}|\boldsymbol{\gamma},\sigma^2_\beta)\Pr(\boldsymbol{\gamma}|\tau_0,\tau_1)\Pr(\sigma^2_\beta|a_{\beta,}b_\beta)\}$$

$$= -\frac{1}{2}\sum_{j=1}^{p}\gamma_j\left(\log\left(\sigma^2_{e_j}\right)+\frac{(\hat{\beta}_j-\beta_j)^2}{\sigma^2_{e_j}}\right)$$

$$-\frac{1}{2}\sum_{j=1}^{p}\gamma_j\left(\log(\sigma^2_\beta)+\frac{\beta_j^2}{\sigma^2_\beta}\right)$$

$$+\sum_{j=1}^{p}\gamma_j\log(\pi_j)+(1-\gamma_j)\log(1-\pi_j)$$

$$-(a_\beta+1)\log(\sigma^2_\beta)-b_\beta\sigma_\beta^{-2} \quad (14)$$

In the E-step*:*

We obtained the expectation of log likelihood, $Q = E\left[\log\Pr(\widehat{\boldsymbol{\beta}},\boldsymbol{\beta},\boldsymbol{\Gamma}|\boldsymbol{\tau},\sigma^2_\beta)\right]$.

The posterior distribution of **β and γ changes to be:**

$$\Pr\left(\gamma_j=1\middle|\widehat{\boldsymbol{\beta}},\tau_0^{(t)},\tau_1^{(t)},\left(\sigma^{(t)}\right)^2_\beta\right)\propto\exp\left\{\frac{m_j^2}{2s_j^2}+\log(s_j)-\log\left(\sigma_\beta^{(t)}\right)+\log\left(\pi_j^{(t)}\right)\right\}, \quad (15)$$

$$\Pr\left(\gamma_j=0\middle|\widehat{\boldsymbol{\beta}},\tau_0^{(t)},\tau_1^{(t)}\right)\propto 1-\pi_j^{(t)}. \quad (16)$$

Then posterior distribution of γ_j is,

$$\gamma_j \sim \text{Bernoulli}\left(\frac{\Pr\left(\gamma_j=1\middle|\widehat{\boldsymbol{\beta}},\tau_0^{(t)},\tau_1^{(t)},\left(\sigma^{(t)}\right)^2_\beta\right)}{\Pr\left(\gamma_j=1\middle|\widehat{\boldsymbol{\beta}},\tau_0^{(t)},\tau_1^{(t)},\left(\sigma^{(t)}\right)^2_\beta\right)+\Pr\left(\gamma_j=0\middle|\widehat{\boldsymbol{\beta}},\tau_0^{(t)},\tau_1^{(t)},\left(\sigma^{(t)}\right)^2_\beta\right)}\right) \quad (17)$$

where $m_j=\frac{\hat{\beta}_j\left(\sigma^{(t)}\right)^2_\beta}{\sigma^2_{e_j}+\left(\sigma^{(t)}\right)^2_\beta}$ and $s_j^2=\frac{\sigma^2_{e_j}*\left(\sigma^{(t)}\right)^2_\beta}{\sigma^2_{e_j}+\left(\sigma^{(t)}\right)^2_\beta}$. Next, we recognize from the complete likelihood that the conditional distribution of β_j given $\gamma_j=1$ is normal:

$$\beta_j|\gamma_j = 1 \ \sim N\left(m_j, s_j^2\right)$$

Certainly, $\beta_j = 0$ if $\gamma_j = 0$.

In the M-step, estimation of intercept τ_0 and gene set enrichment parameter τ_1 is just following the same Newton-Raphson algorithm. For the estimation of σ_β^2, we obtain the first derivatives of the Q function with respect to σ_β^2 as

$$\frac{\partial Q}{\partial \sigma_\beta^2} = \sigma_\beta^{-4}\left(\sum_{j=1}^{p} \frac{E(\gamma_j\beta_j^2)}{2} + b_\beta\right) - \sigma_\beta^{-2}\left(\frac{\sum_{j=1}^{p} E(\gamma_j)}{2} + a_\beta + 1\right),$$

which leads to an analytical update for σ_β^2 as

$$\left(\sigma_\beta^{(t+1)}\right)^2 = \frac{\sum_{j=1}^{p} \frac{E(\gamma_j\beta_j^2)}{2} + b_\beta}{\frac{\sum_{j=1}^{p} E(\gamma_j)}{2} + a_\beta + 1}. \quad (18)$$

Simulations and real data applications in iDEA variant

Following the same procedures of simulations in manuscript, we compared the performance of iDEA modeling on beta effect size with the commonly used GSE analysis methods fGSEA, CAMERA, PAGE and GSEA. We found that consistent with the results generated from modeling the z score, iDEA produces well-calibrated *p*-values under the null in different simulation scenarios (Supplementary Figure 31) despite slight inflation when the number of DE genes is small. Besides type I error control, we found that iDEA is more powerful than the other GSE methods across a range of alternative scenarios at a fixed false discovery rate (FDR) of 5% (Supplementary Figure 32). For DE analysis, we found that iDEA can improve DE analysis power regardless of whether the summary statistics are from MAST, edgeR or zingeR (Supplementary Figure 33). For example, with $\tau_1 = 5$ and CR = 10%, iDEA achieves a power of 84%, 61% and 84% at a true FDR of 5%, when it uses the input summary statistics obtained from zingeR, MAST and edgeR, respectively. In contrast, the power of these three different DE methods are 66%, 53%, and 67%, respectively (Supplementary Figure 33).

We also compared the performance of iDEA modeling on beta effect size in DE analysis and GSE analysis in the mouse neuronal scRNAseq dataset (Supplementary Figure 34). Consistent with simulations, the GSE *p*-values in the permuted data from iDEA ($\lambda_{\text{gc}} = 1.19$), fGSEA ($\lambda_{\text{gc}} = 1.08$), PAGE ($\lambda_{\text{gc}} = 0.99$), and GSEA ($\lambda_{\text{gc}} = 0.94$) are all well-behaved, while the *p*-values from CAMERA show severe deflation ($\lambda_{\text{gc}} = 011$) (Supplementary Figure 34A). Also consistent with simulations, iDEA identified more significantly enriched gene sets compared

to the other methods (Supplementary Figure 34B). For example, at an FDR of 5%, iDEA identified 1205 enriched gene sets, which is four times higher than the second-best method (GSEA, 246). In contrast, fGSEA, CAMERA and PAGE identified 236, 134, and 205 enriched gene sets, respectively. Many of the top 1% enriched gene sets are associated with neuron structures and functions such as neuron projection (GO:0043005)[17], neuron part (GO:0097458)[18] axon (GO:0030424)[19], synapse (GO:0045202)[20] and ion transport (GO:0006811)[20]. In addition, use of iDEA recovered 98 out of the 237 gene sets known to be involved in inflammatory itch as provided by the original paper[9]. In contrast, fGSEA, CAMERA, PAGE, and fGSEA identified 31, 20, 19, and 29 gene sets among them, respectively. The recovery of more enriched gene sets relevant to inflammation and itch by iDEA compared to the other GSE methods provides convergent support for the greater power of iDEA when modeling on beta effect size directly compared to the other methods for GSE analysis.

We next applied iDEA for DE analysis to identify DE genes with adding the gene set neuron part (GO:0097458). Again, iDEA identified more DE genes than zingeR (Supplementary Figure 34C). At an FDR of 1%, iDEA detected 1,222 DE genes, which is 23.0% higher than zingeR (993). Importantly, consistent with the power gain brought by iDEA, it detected 77 DE genes out of top 100 previously known NP1 DE genes listed in the original study, while zingeR detected 75 DE genes.

Supplementary Note 5. Bayesian model averaging (BMA) approach

Besides performing DE analysis in iDEA in the real data based on a pre-selected gene set, we also developed a new strategy to aggregate DE evidence on a particular gene across all gene sets through Bayesian model averaging (BMA). Specifically, for the given gene, we denote its posterior inclusion probability (PIP) obtained using the gene set *k* as PIP_k. The corresponding Bayes factor quantifying its DE evidence based on the gene set *k* is $\mathrm{BF}_k = \mathrm{PIP}_k/(1-\mathrm{PIP}_k)$. With equal prior weights on different gene sets, the average Bayes factor quantifying its DE evidence based on all *K* gene sets is thus $\mathrm{ABF} = \frac{1}{K}\sum_{k=1}^{K}\mathrm{BF}_k$, which can be converted back to a posterior inclusion probability as $\mathrm{PIP} = \mathrm{ABF}/(1+\mathrm{ABF})$. We found that PIPs computed this way is highly correlated with the PIPs computed based on the pre-selected gene set (Supplementary Figure 35). We now provide both options for computing PIPs for quantifying DE evidence: biologists can choose to use pre-selected gene sets that are known to be relevant to the particular experiments, as is the case for all the real data applications; alternatively,

biologists also have the option of using the Bayesian model averaging when such prior knowledge is not available.

Supplementary Note 6. Gene set overlap

Previously we followed most existing GSE approaches and accounted for test non-independence due to gene set overlap through permutation. In addition, we also performed new analysis to further examine the issue of gene set overlap in the real data applications. We adopted the method proposed by Jiang and Gentleman[21] to examines pairs of gene sets one at a time. For each pair of gene set, Jiang's method divides genes into three categories: one category of genes that are only in the first gene set, one category of genes that are only in the second gene set, and one category of genes that are common in both gene sets. Afterwards, Jiang's method calculates three *p*-values, one for each category of genes. By computing *p*-values in each set, we can explicitly deconvolute the results in the presence of gene set overlap. Here, we mainly applied Jiang's method to analyze the top 50 gene sets identified by iDEA in human embryonic data and mouse neuron cell data in order to further dissect particular set of genes that drive the enrichment signal. (Note that we did not apply to all significant gene sets due to the heavy computational burden of Jiang's method and the gene set overlap is moderate compare to the gene set size). Specifically, there are 1,225 pairwise combinations among top 50 gene sets. For each real data we checked, we first construct the pairwise combinations of gene sets among top 50 significant gene sets identified by iDEA and for each pair, and then we filtered out gene set pairs which has less than 20 genes overlap (due to computational stability). For each pair which has larger than 20 genes in overlap, we calculated above mentioned three categories of *p*-values. Then we checked the *p*-values of the category of genes that are common in both gene sets and the *p*-values of the category of genes that are unique in gene sets respectively. For example, in the human embryonic data, 692 of the 1,225 gene set pairs have higher than 20 genes in overlap. For each of these 692 gene set pairs in turn, we calculated the three *p*-values as mentioned in the previous paragraph. Among the total 2,076 adjusted *p*-values (Bonferroni correction) we calculated, 1,397 of them are less than 0.05. We first look at the intersection part, 35 out of 692 intersection sets have adjusted *p*-value is less than 0.05. For the disjoint parts, 1,362 out of 1,384 are significant. This observation suggests that among the top 50 significant gene sets we identified, gene set specific genes are significantly enriched, suggesting that it is not the overlapped genes that drive the enrichment signal and that gene set overlap does not appear to introduce excessive false signals. We further looked at the combination of the

top first gene set GO:0001944 (vasculature development) (Supplementary Table 10). From the table, we observed that the significance of this gene set is induced by both the overlapping parts and non-overlapping parts. Following the same procedure, we also applied Jiang's method to analyze the top 50 gene sets identified by iDEA in the mouse sensory neuron scRNA-seq data. 1,025 out of 1,225 gene set pairs have higher than 20 genes in overlap. For each of these 1,025 gene set pairs in turn, we calculated the three *p*-values as mentioned in the previous paragraph. Among the total 3,075 adjusted *p*-values (Bonferroni correction) we calculated, 2,603 of them are less than 0.05. We first look at the intersection part, 889 out of 1,025 intersection sets have adjusted *p*-value is less than 0.05. For the disjoint parts, 1,714 out of 2,050 are significant. We further looked at the combination of the top first gene set GO:0044425 (obsolete membrane part) (Supplementary Table 11). From the table, we observed that the significance of this gene set is induced by both the overlapping parts and non-overlapping parts.

Supplementary Note 7. Cell types identification in the three scRNA-seq datasets

For all the real datasets we analyzed, one of our real data contains cell types that are known *a priori* and not inferred from the whole expression matrix, while the other two data contain cell types that are extensively validated through approaches other than inferring based on the whole expression matrix. Specifically, for the human embryonic stem cell scRNAseq dataset, the cell types are obtained from fluorescence-activated cell sorting (FACS) analysis before mixing for scRNA-seq. FACS relies on known cell type markers and represents a somewhat unbiased strategy for cell type clustering[22]. For the mouse neuronal scRNAseq dataset, the cell types are initially inferred through an iterative PCA-based procedure and are further validated by comparing the hierarchical relationship of the neuronal types with the known developmental origin of sensory neuron types, as well as by comparing neurons with distinct and characteristic soma sizes in their identified neuronal class. In addition, the inferred neuronal cell types are further confirmed by double and triple immunohistochemical staining (e.g. NP1 cell type by staining of *PLXNC1*). For the 10x Genomics PBMC scRNASeq dataset, the identity of cell types was inferred by aligning cluster-specific genes to known markers of distinct PBMC populations as well as comparing against the transcriptomes of the purified populations in PBMC subsets. Their approach has been found to be largely consistent with conventional marker-based methods and the major cell types reach to the expected ratios in PBMCs. We have also displayed t-SNE plot in

Supplementary Figure 22, which clearly shows distinct cell clusters. Because the cell types in these data are validated through various approaches, the DE analysis results are less likely influenced by the cell type inference step as compared to other data that are fully relying on the whole gene expression matrix for cell type inference.

Supplementary Tables

Supplementary Table 1. The results from iDEA to detect top 50 enriched gene sets on human embryonic stem cell scRNA-seq data

Rank	Gene Set	Count	Coefficient	Variance	*P*-value
1	GO_VASCULATURE_DEVELOPMENT	429	0.000	0.016	7.340E-18
2	GO_BLOOD_VESSEL_MORPHOGENESIS	332	1.203	0.021	1.070E-16
3	SCHUETZ_BREAST_CANCER_DUCTAL_INVASIVE_UP	310	1.267	0.024	3.650E-16
4	GO_ANGIOGENESIS	268	1.344	0.028	1.310E-15
5	LIU_PROSTATE_CANCER_DN	424	0.992	0.016	1.930E-15
6	SWEET_LUNG_CANCER_KRAS_DN	377	1.048	0.018	4.590E-15
7	ONDER_CDH1_TARGETS_2_DN	384	1.020	0.017	8.180E-15
8	SMID_BREAST_CANCER_NORMAL_LIKE_UP	361	1.110	0.021	9.870E-15
9	GO_SINGLE_ORGANISM_CELL_ADHESION	370	1.024	0.018	3.300E-14
10	GO_ANCHORING_JUNCTION	468	0.848	0.013	5.390E-14
11	LIM_MAMMARY_STEM_CELL_UP	443	0.906	0.015	8.270E-14
12	PASINI_SUZ12_TARGETS_DN	305	1.076	0.021	1.410E-13
13	BOQUEST_STEM_CELL_DN	201	1.543	0.044	2.220E-13
14	CHARAFE_BREAST_CANCER_LUMINAL_VS_MESENCHYMAL_DN	442	0.853	0.014	4.830E-13
15	BENPORATH_ES_1	372	0.902	0.016	4.910E-13
16	GO_REGULATION_OF_SYSTEM_PROCESS	390	0.935	0.017	1.140E-12
17	GO_APICAL_PART_OF_CELL	281	1.108	0.024	1.210E-12
18	GO_EXTRACELLULAR_MATRIX	326	1.011	0.020	1.440E-12
19	GO_CELL_CELL_JUNCTION	335	0.976	0.019	1.610E-12
20	DELYS_THYROID_CANCER_UP	379	0.933	0.017	1.770E-12
21	GO_WOUND_HEALING	393	0.893	0.016	2.510E-12
22	RODWELL_AGING_KIDNEY_UP	418	0.863	0.016	4.190E-12
23	CHARAFE_BREAST_CANCER_LUMINAL_VS_BASAL_DN	402	0.863	0.016	4.500E-12
24	GO_REGULATION_OF_VASCULATURE_DEVELOPMENT	200	1.387	0.040	5.160E-12
25	SMID_BREAST_CANCER_LUMINAL_B_DN	428	0.853	0.015	6.110E-12
26	SABATES_COLORECTAL_ADENOMA_DN	198	1.342	0.039	9.900E-12
27	GO_LEUKOCYTE_MIGRATION	191	1.399	0.043	1.550E-11
28	LINDGREN_BLADDER_CANCER_CLUSTER_2B	353	0.909	0.018	2.180E-11
29	GO_TAXIS	366	0.892	0.018	2.540E-11

30	GO_POSITIVE_REGULATION_OF_MAPK_CASCADE	393	0.845	0.016	2.560E-11
31	CREIGHTON_ENDOCRINE_THERAPY_RESISTANCE_5	431	0.793	0.014	2.880E-11
32	GO_REGULATION_OF_BODY_FLUID_LEVELS	410	0.825	0.016	3.680E-11
33	GO_REGULATION_OF_NEURON_PROJECTION_DEVELOPMENT	364	0.857	0.017	4.570E-11
34	DUTERTRE_ESTRADIOL_RESPONSE_24HR_DN	487	0.732	0.012	5.480E-11
35	GO_MEMBRANE_MICRODOMAIN	259	1.038	0.025	6.770E-11
36	GSE2405_0H_VS_3H_A_PHAGOCYTOPHILUM_STIM_NEUTROPHIL_UP	178	1.335	0.043	1.400E-10
37	WANG_RESPONSE_TO_GSK3_INHIBITOR_SB216763_DN	340	0.831	0.017	1.500E-10
38	GO_CELL_SUBSTRATE_JUNCTION	385	0.791	0.015	1.520E-10
39	GO_CELL_LEADING_EDGE	323	0.879	0.019	1.640E-10
40	REACTOME_HEMOSTASIS	386	0.815	0.016	1.660E-10
41	GO_NEGATIVE_REGULATION_OF_LOCOMOTION	227	1.075	0.028	1.710E-10
42	GO_ACTIN_FILAMENT_BASED_PROCESS	408	0.777	0.015	1.890E-10
43	SCHAEFFER_PROSTATE_DEVELOPMENT_48HR_UP	425	0.757	0.014	2.070E-10
44	GO_POSITIVE_REGULATION_OF_CELL_DEVELOPMENT	407	0.779	0.015	2.120E-10
45	GO_CELL_MORPHOGENESIS_INVOLVED_IN_DIFFERENTIATION	456	0.734	0.013	2.600E-10
46	GO_POSITIVE_REGULATION_OF_NERVOUS_SYSTEM_DEVELOPMENT	374	0.801	0.016	3.540E-10
47	CHYLA_CBFA2T3_TARGETS_UP	324	0.893	0.020	4.210E-10
48	VERHAAK_AML_WITH_NPM1_MUTATED_DN	205	1.165	0.035	4.230E-10
49	GO_REGULATION_OF_CELL_MORPHOGENESIS_INVOLVED_IN_DIFFERENTIATION	304	0.870	0.020	5.390E-10
50	WALLACE_PROSTATE_CANCER_RACE_UP	232	1.038	0.029	8.030E-10

P-values were determined by two-sided Wald test.

Supplementary Table 2. The results from iDEA to detect top 50 enriched gene sets on mouse neuronal cell scRNA-seq data

Rank	Gene Ontology	Gene Ontology Term	Count	Coefficient	Variance	*P*-value
1	GO:0044425	membrane part	3688	1.040	0.004	2.260E-72
2	GO:0043005	neuron projection	1121	1.240	0.007	8.680E-63
3	GO:0071944	cell periphery	2840	0.996	0.005	4.630E-63
4	GO:0016020	membrane	5081	0.995	0.005	3.970E-63
5	GO:0097458	neuron part	1472	1.140	0.006	4.200E-62
6	GO:0005886	plasma membrane	2762	0.994	0.005	2.510E-62
7	GO:0031224	intrinsic component of membrane	2845	0.934	0.005	6.740E-56
8	GO:0044459	plasma membrane part	1498	1.040	0.006	3.880E-51
9	GO:0045202	synapse	1108	1.130	0.007	2.210E-50
10	GO:0006811	ion transport	978	1.180	0.008	3.850E-51
11	GO:0044456	synapse part	867	1.190	0.009	1.420E-47
12	GO:0016021	integral component of membrane	2758	0.877	0.005	2.020E-48
13	GO:0006812	cation transport	699	1.280	0.010	6.760E-48
14	GO:0030424	axon	451	1.460	0.014	1.420E-44
15	GO:0120025	plasma membrane bounded cell projection	1588	0.948	0.006	3.180E-43
16	GO:0036477	somatodendritic compartment	783	1.180	0.010	8.090E-43
17	GO:0015672	monovalent inorganic cation transport	306	1.650	0.019	1.430E-42
18	GO:0098800	inner mitochondrial membrane protein complex	101	2.700	0.054	1.410E-37
19	GO:0042995	cell projection	1726	0.891	0.006	1.080E-39
20	GO:0043269	regulation of ion transport	466	1.360	0.014	3.400E-39
21	GO:0098798	mitochondrial protein complex	126	2.230	0.040	9.350E-35
22	GO:0043209	myelin sheath	179	1.910	0.029	1.270E-35
23	GO:0032879	regulation of localization	1923	0.828	0.006	3.380E-36
24	GO:0051049	regulation of transport	1307	0.920	0.007	1.220E-35
25	GO:0044455	mitochondrial membrane part	181	1.860	0.029	3.370E-34
26	GO:0034220	ion transmembrane transport	560	1.210	0.013	5.690E-35
27	GO:0031226	intrinsic component of plasma membrane	785	1.060	0.010	6.070E-34
28	GO:0030425	dendrite	557	1.180	0.013	9.280E-33
29	GO:0098793	presynapse	444	1.280	0.015	8.470E-33
30	GO:0099536	synaptic signaling	526	1.200	0.013	1.460E-32

31	GO:0098590	plasma membrane region	785	1.040	0.010	1.990E-32
32	GO:0042391	regulation of membrane potential	316	1.440	0.019	7.980E-32
33	GO:0005887	integral component of plasma membrane	742	1.050	0.011	9.240E-32
34	GO:0098660	inorganic ion transmembrane transport	430	1.280	0.016	1.290E-31
35	GO:0099537	trans-synaptic signaling	518	1.190	0.014	2.100E-31
36	GO:0008324	cation transmembrane transporter activity	404	1.310	0.017	5.890E-32
37	GO:0098916	anterograde trans-synaptic signaling	510	1.190	0.014	6.310E-31
38	GO:0007268	chemical synaptic transmission	510	1.190	0.014	6.310E-31
39	GO:0090304	nucleic acid metabolic process	3107	-0.965	0.009	5.220E-31
40	GO:0055085	transmembrane transport	785	1.020	0.010	5.800E-31
41	GO:0098662	inorganic cation transmembrane transport	400	1.300	0.017	8.720E-31
42	GO:0015077	monovalent inorganic cation transmembrane transporter activity	222	1.620	0.026	1.630E-30
43	GO:0098655	cation transmembrane transport	451	1.230	0.015	1.820E-30
44	GO:0070469	respiratory chain	63	3.060	0.095	8.120E-27
45	GO:0097060	synaptic membrane	395	1.280	0.017	1.110E-29
46	GO:0003008	system process	975	0.929	0.009	1.050E-29
47	GO:0023052	signaling	3404	0.662	0.005	2.460E-29
48	GO:0005746	mitochondrial respiratory chain	60	3.030	0.097	1.720E-25
49	GO:0003676	nucleic acid binding	2219	-1.240	0.016	6.300E-30
50	GO:0007186	G-protein coupled receptor signaling pathway	393	1.270	0.017	8.620E-29

P-values were determined by two-sided Wald test.

Supplementary Table 3. The results from iDEA to detect enriched gene signature on 10x Genomics PBMC scRNA-seq data

Rank	Signature	Project	Coefficient	Variance	*P*-value
1	CD8+ Tem	BLUEPRINT	1.780	0.121	2.960E-07
2	CD8+ Tem	NOVERSHTERN	2.240	0.359	1.860E-04
3	CD4+ memory T-cells	FANTOM	0.915	0.062	2.270E-04
4	CD8+ Tem	NOVERSHTERN	2.064	0.325	2.980E-04
5	CD4+ memory T-cells	FANTOM	0.798	0.054	5.770E-04
6	CD8+ Tcm	NOVERSHTERN	2.220	0.432	7.310E-04
7	CD8+ Tem	NOVERSHTERN	1.766	0.280	8.450E-04
8	CD8+ T-cells	HPCA	1.618	0.236	8.710E-04
9	CD8+ Tem	HPCA	1.039	0.107	1.468E-03
10	CD8+ Tem	BLUEPRINT	0.934	0.087	1.574E-03
11	NK cells	HPCA	1.558	0.250	1.827E-03
12	CD4+ naive T-cells	FANTOM	1.322	0.180	1.829E-03
13	CD8+ T-cells	BLUEPRINT	1.962	0.407	2.090E-03
14	CD8+ Tcm	NOVERSHTERN	1.727	0.330	2.637E-03
15	Basophils	NOVERSHTERN	1.765	0.356	3.082E-03
16	CD4+ naive T-cells	FANTOM	1.950	0.438	3.197E-03
17	CD8+ Tcm	HPCA	1.316	0.201	3.381E-03
18	Tgd cells	HPCA	0.983	0.113	3.493E-03
19	CD8+ T-cells	FANTOM	1.805	0.385	3.620E-03
20	CD8+ T-cells	NOVERSHTERN	1.800	0.385	3.734E-03
21	CD4+ naive T-cells	NOVERSHTERN	1.965	0.460	3.771E-03
22	CD8+ Tem	HPCA	0.924	0.118	7.164E-03
23	CD4+ naive T-cells	IRIS	1.141	0.182	7.521E-03
24	CD8+ T-cells	FANTOM	1.483	0.313	7.986E-03
25	CD8+ T-cells	BLUEPRINT	1.470	0.310	8.225E-03
26	GMP	BLUEPRINT	0.994	0.192	2.322E-02
27	CLP	BLUEPRINT	1.191	0.279	2.414E-02
28	NK cells	BLUEPRINT	1.367	0.371	2.487E-02
29	CD4+ Tem	NOVERSHTERN	0.965	0.214	3.713E-02
30	Plasma cells	BLUEPRINT	-0.390	0.036	3.849E-02
31	CD4+ Tem	HPCA	0.962	0.216	3.853E-02
32	CD4+ Tcm	NOVERSHTERN	0.906	0.201	4.318E-02

33	CD4+ Tcm	HPCA	0.788	0.170	5.575E-02
34	MPP	BLUEPRINT	0.744	0.157	6.007E-02
35	Tgd cells	HPCA	0.594	0.106	6.837E-02
36	Tgd cells	HPCA	0.629	0.121	7.110E-02
37	B-cells	NOVERSHTERN	1.051	0.340	7.134E-02
38	NK cells	HPCA	1.042	0.341	7.441E-02
39	CD8+ naive T-cells	HPCA	1.218	0.475	7.723E-02
40	GMP	BLUEPRINT	0.794	0.208	8.173E-02
41	CD8+ naive T-cells	HPCA	1.199	0.480	8.360E-02
42	CD4+ naive T-cells	HPCA	1.047	0.390	9.346E-02
43	Hepatocytes	HPCA	-0.545	0.111	1.014E-01
44	CD8+ naive T-cells	HPCA	0.944	0.344	1.076E-01
45	Hepatocytes	HPCA	-0.679	0.183	1.122E-01
46	GMP	BLUEPRINT	0.645	0.166	1.134E-01
47	Hepatocytes	HPCA	-0.670	0.182	1.164E-01
48	CD4+ Tcm	HPCA	0.785	0.266	1.281E-01
49	CMP	HPCA	0.950	0.395	1.309E-01
50	CD8+ Tcm	BLUEPRINT	0.950	0.415	1.406E-01

P-values were determined by two-sided Wald test.

Supplementary Table 4. **The results from fGSEA to detect enriched gene signature on 10x Genomics PBMC scRNA-seq data**

Rank	Signature	Project	*P*-value	Adjust *p*-value
1	CD4+ memory T-cells	FANTOM	2.490E-04	3.987E-03
2	CD8+ Tem	NOVERSHTERN	2.740E-04	3.987E-03
3	CD8+ Tem	NOVERSHTERN	2.790E-04	3.987E-03
4	NK cells	HPCA	2.840E-04	3.987E-03
5	CD8+ Tem	NOVERSHTERN	2.870E-04	3.987E-03
6	NK cells	HPCA	2.870E-04	3.987E-03
7	NK cells	HPCA	2.970E-04	3.987E-03
8	CD8+ Tem	BLUEPRINT	3.400E-04	3.987E-03
9	CD8+ Tem	HPCA	3.550E-04	3.987E-03
10	Tgd cells	HPCA	3.580E-04	3.987E-03
11	CD8+ Tem	HPCA	3.600E-04	3.987E-03
12	Tgd cells	HPCA	3.650E-04	3.987E-03
13	Tgd cells	HPCA	3.820E-04	3.987E-03
14	CD8+ Tem	BLUEPRINT	3.880E-04	3.987E-03
15	CD4+ memory T-cells	FANTOM	5.130E-04	4.925E-03
16	NK cells	BLUEPRINT	5.470E-04	4.925E-03
17	CD8+ Tcm	NOVERSHTERN	8.210E-04	6.953E-03
18	MPP	BLUEPRINT	1.002E-03	8.016E-03
19	CD8+ Tcm	HPCA	1.225E-03	9.285E-03
20	CD8+ Tcm	NOVERSHTERN	1.420E-03	9.320E-03
21	MPP	FANTOM	1.472E-03	9.320E-03
22	NK cells	BLUEPRINT	1.472E-03	9.320E-03
23	MPP	FANTOM	1.489E-03	9.320E-03
24	CD8+ T-cells	HPCA	6.771E-03	4.062E-02
25	Mesangial cells	FANTOM	9.177E-03	5.286E-02
26	Erythrocytes	NOVERSHTERN	1.333E-02	7.385E-02
27	Basophils	NOVERSHTERN	1.587E-02	8.463E-02
28	MPP	BLUEPRINT	2.187E-02	1.124E-01
29	Endothelial cells	BLUEPRINT	2.400E-02	1.191E-01
30	CD4+ Tcm	HPCA	2.602E-02	1.249E-01
31	mv Endothelial cells	FANTOM	2.773E-02	1.288E-01
32	CD4+ naive T-cells	NOVERSHTERN	2.870E-02	1.292E-01

33	CD4+ T-cells	FANTOM	3.786E-02	1.652E-01
34	CD4+ memory T-cells	IRIS	4.213E-02	1.784E-01
35	CMP	HPCA	5.823E-02	2.396E-01
36	Platelets	HPCA	7.049E-02	2.812E-01
37	CD4+ naive T-cells	IRIS	7.615E-02	2.812E-01
38	CD8+ T-cells	BLUEPRINT	7.718E-02	2.812E-01
39	CD8+ T-cells	FANTOM	7.718E-02	2.812E-01
40	CD4+ Tcm	NOVERSHTERN	8.359E-02	2.812E-01
41	Platelets	HPCA	8.469E-02	2.812E-01
42	Keratinocytes	FANTOM	8.705E-02	2.812E-01
43	Epithelial cells	ENCODE	9.415E-02	2.812E-01
44	CD8+ T-cells	BLUEPRINT	9.493E-02	2.812E-01
45	Epithelial cells	HPCA	9.501E-02	2.812E-01
46	Keratinocytes	ENCODE	9.501E-02	2.812E-01
47	Keratinocytes	HPCA	9.501E-02	2.812E-01
48	Keratinocytes	HPCA	9.501E-02	2.812E-01
49	Epithelial cells	HPCA	9.598E-02	2.812E-01
50	Macrophages	BLUEPRINT	9.935E-02	2.812E-01

P-values were determined by Kolmogorov Smirnov test implemented in fGSEA and adjusted by Benjamin & Hochberg method.

Supplementary Table 5. The results from CAMERA to detect enriched gene signature on 10x Genomics PBMC scRNA-seq data

Rank	Signature	Project	*P*-value	Adjust *p*-value
1	CD8+ Tem	BLUEPRINT	5.650E-22	8.140E-20
2	CD8+ Tem	HPCA	2.270E-13	1.640E-11
3	Tgd cells	HPCA	6.660E-13	3.190E-11
4	CD8+ Tem	HPCA	5.270E-12	1.900E-10
5	NK cells	HPCA	9.870E-12	2.840E-10
6	CD8+ Tem	BLUEPRINT	2.570E-11	6.150E-10
7	NK cells	HPCA	2.990E-11	6.150E-10
8	Tgd cells	HPCA	5.960E-10	1.070E-08
9	CD8+ Tem	NOVERSHTERN	1.610E-07	2.500E-06
10	NK cells	HPCA	1.740E-07	2.500E-06
11	CD8+ Tem	NOVERSHTERN	2.140E-07	2.800E-06
12	Tgd cells	HPCA	2.570E-07	2.960E-06
13	CD8+ Tcm	NOVERSHTERN	2.670E-07	2.960E-06
14	CD8+ Tcm	HPCA	4.330E-07	4.450E-06
15	CD8+ Tem	NOVERSHTERN	9.720E-07	9.330E-06
16	MPP	FANTOM	1.280E-06	1.150E-05
17	Basophils	NOVERSHTERN	1.620E-06	1.370E-05
18	NK cells	BLUEPRINT	1.970E-06	1.580E-05
19	MPP	FANTOM	5.800E-06	4.400E-05
20	CD8+ Tcm	NOVERSHTERN	9.700E-06	6.980E-05
21	CD8+ T-cells	HPCA	8.830E-05	6.050E-04
22	CD4+ memory T-cells	FANTOM	5.620E-04	3.581E-03
23	NK cells	BLUEPRINT	5.780E-04	3.581E-03
24	CD4+ memory T-cells	FANTOM	5.970E-04	3.581E-03
25	Mesangial cells	FANTOM	2.573E-03	1.482E-02
26	CD4+ memory T-cells	IRIS	6.846E-03	3.792E-02
27	Endothelial cells	BLUEPRINT	1.110E-02	5.920E-02
28	CD8+ T-cells	BLUEPRINT	1.226E-02	6.302E-02
29	MPP	BLUEPRINT	1.621E-02	8.013E-02
30	CD4+ Tcm	HPCA	1.669E-02	8.013E-02
31	CD4+ naive T-cells	IRIS	2.081E-02	9.274E-02
32	CD8+ T-cells	BLUEPRINT	2.125E-02	9.274E-02

33	CD8+ T-cells	FANTOM	2.125E-02	9.274E-02
34	Erythrocytes	NOVERSHTERN	2.239E-02	9.481E-02
35	Platelets	HPCA	3.350E-02	1.378E-01
36	CMP	HPCA	3.685E-02	1.474E-01
37	Platelets	HPCA	4.157E-02	1.608E-01
38	CD4+ T-cells	FANTOM	4.245E-02	1.608E-01
39	mv Endothelial cells	FANTOM	4.355E-02	1.608E-01
40	MPP	BLUEPRINT	5.160E-02	1.858E-01
41	Astrocytes	FANTOM	5.475E-02	1.923E-01
42	Macrophages	BLUEPRINT	5.656E-02	1.939E-01
43	CD4+ Tcm	NOVERSHTERN	5.890E-02	1.973E-01
44	Pericytes	ENCODE	7.593E-02	2.485E-01
45	CD8+ Tcm	BLUEPRINT	1.048E-01	3.355E-01
46	CLP	BLUEPRINT	1.229E-01	3.846E-01
47	Epithelial cells	HPCA	5.002E-02	1.596E-01
48	Keratinocytes	ENCODE	5.002E-02	1.563E-01
49	Keratinocytes	HPCA	5.002E-02	1.531E-01
50	Keratinocytes	HPCA	5.002E-02	1.500E-01

P-values were determined by two-sided t-test implemented in CAMERA and adjusted by Benjamin & Hochberg method.

Supplementary Table 6. The results from PAGE to detect enriched gene signature on 10x Genomics PBMC scRNA-seq data

Rank	Signature	Project	Coefficient	*P*-value	FDR
1	CD8+ Tem	BLUEPRINT	13.915	5.140E-44	0.000
2	CD8+ Tem	HPCA	10.681	1.240E-26	0.000
3	Tgd cells	HPCA	10.443	1.580E-25	0.000
4	CD8+ Tem	BLUEPRINT	9.879	5.150E-23	0.000
5	CD8+ Tem	HPCA	9.571	1.060E-21	0.000
6	Tgd cells	HPCA	9.443	3.610E-21	0.000
7	NK cells	HPCA	9.317	1.200E-20	0.000
8	CD8+ Tem	NOVERSHTERN	9.003	2.190E-19	0.000
9	CD8+ Tem	NOVERSHTERN	8.922	4.580E-19	0.000
10	CD8+ Tem	NOVERSHTERN	8.407	4.210E-17	0.000
11	NK cells	HPCA	8.296	1.080E-16	0.000
12	Tgd cells	HPCA	8.241	1.710E-16	0.000
13	CD8+ Tcm	NOVERSHTERN	7.757	8.690E-15	0.000
14	CD8+ Tcm	HPCA	7.508	6.020E-14	0.000
15	NK cells	BLUEPRINT	6.726	1.750E-11	0.000
16	Basophils	NOVERSHTERN	6.698	2.110E-11	0.000
17	CD8+ Tcm	NOVERSHTERN	6.680	2.380E-11	0.000
18	NK cells	HPCA	6.622	3.530E-11	0.000
19	CD8+ T-cells	HPCA	6.385	1.710E-10	0.000
20	NK cells	BLUEPRINT	5.254	1.490E-07	0.000
21	MPP	FANTOM	4.911	9.070E-07	0.000
22	MPP	FANTOM	4.622	3.800E-06	0.000
23	CD4+ memory T-cells	FANTOM	-3.700	2.150E-04	0.017
24	CD4+ memory T-cells	IRIS	3.690	2.240E-04	0.021
25	CD4+ memory T-cells	FANTOM	-3.660	2.520E-04	0.020
26	CD4+ T-cells	FANTOM	-2.990	2.793E-03	0.050
27	CD4+ Tcm	NOVERSHTERN	-2.960	3.077E-03	0.052
28	CD4+ Tcm	HPCA	-2.926	3.433E-03	0.050
29	Erythrocytes	NOVERSHTERN	2.849	4.392E-03	0.048
30	CD8+ Tcm	BLUEPRINT	2.748	5.995E-03	0.047
31	Endothelial cells	BLUEPRINT	2.608	9.098E-03	0.048
32	CD8+ T-cells	BLUEPRINT	2.534	1.129E-02	0.069

33	Platelets	HPCA	2.503	1.233E-02	0.082
34	CD4+ naive T-cells	NOVERSHTERN	-2.442	1.461E-02	0.082
35	MPP	BLUEPRINT	-2.434	1.492E-02	0.080
36	CMP	HPCA	2.325	2.005E-02	0.100
37	Mesangial cells	FANTOM	2.319	2.038E-02	0.100
38	CD8+ T-cells	BLUEPRINT	2.312	2.078E-02	0.103
39	CD8+ T-cells	FANTOM	2.312	2.078E-02	0.100
40	Platelets	HPCA	2.310	2.090E-02	0.098
41	CD4+ naive T-cells	IRIS	-2.242	2.497E-02	0.105
42	CD4+ Tem	HPCA	-2.221	2.636E-02	0.107
43	CD4+ naive T-cells	HPCA	-2.122	3.385E-02	0.116
44	Macrophages	BLUEPRINT	2.043	4.101E-02	0.139
45	Keratinocytes	FANTOM	-1.966	4.935E-02	0.164
46	mv Endothelial cells	FANTOM	1.963	4.966E-02	0.161
47	Epithelial cells	HPCA	-1.960	5.002E-02	0.160
48	Keratinocytes	ENCODE	-1.960	5.002E-02	0.156
49	Keratinocytes	HPCA	-1.960	5.002E-02	0.153
50	Keratinocytes	HPCA	-1.960	5.002E-02	0.150

P-values were determined by two-sided t-test implemented in CAMERA.

Supplementary Table 7. The results from GSEA to detect enriched gene signature on 10x Genomics PBMC scRNA-seq data

Rank	Signature	Project	Coefficient	*P*-value	FDR
1	CD4+ MEMORY T-Cells	FANTOM	-0.614	0.000E+00	0.036
2	CD4+ MEMORY T-Cells	FANTOM	-0.622	0.000E+00	0.026
3	CD8+ T-Cells	HPCA	0.752	0.000E+00	0.023
4	CD8+ TCM	HPCA	0.762	0.000E+00	0.006
5	CD8+ TCM	NOVERSHTERN	0.822	0.000E+00	0.007
6	CD8+ TEM	BLUEPRINT	0.766	0.000E+00	0.000
7	CD8+ TEM	BLUEPRINT	0.894	0.000E+00	0.000
8	CD8+ TEM	HPCA	0.803	0.000E+00	0.000
9	CD8+ TEM	HPCA	0.816	0.000E+00	0.000
10	CD8+ TEM	NOVERSHTERN	0.922	0.000E+00	0.000
11	CD8+ TEM	NOVERSHTERN	0.895	0.000E+00	0.001
12	MPP	FANTOM	0.781	0.000E+00	0.002
13	NK Cells	BLUEPRINT	0.892	0.000E+00	0.001
14	NK Cells	HPCA	0.884	0.000E+00	0.000
15	NK Cells	HPCA	0.920	0.000E+00	0.000
16	NK Cells	HPCA	0.863	0.000E+00	0.001
17	TGD Cells	HPCA	0.816	0.000E+00	0.000
18	TGD Cells	HPCA	0.762	0.000E+00	0.000
19	TGD Cells	HPCA	0.770	0.000E+00	0.000
20	MPP	BLUEPRINT	-0.698	1.422E-03	0.032
21	CD8+ TCM	NOVERSHTERN	0.870	2.513E-03	0.006
22	CD8+ TEM	NOVERSHTERN	0.876	2.740E-03	0.001
23	MPP	FANTOM	0.799	2.890E-03	0.006
24	NK Cells	BLUEPRINT	0.798	5.814E-03	0.004
25	MESANGIAL Cells	FANTOM	0.756	8.671E-03	0.030
26	BASOPHILS	NOVERSHTERN	0.788	1.405E-02	0.045
27	ERYTHROCYTES	NOVERSHTERN	0.808	2.057E-02	0.034
28	CD4+ NAIVE T-Cells	NOVERSHTERN	-0.719	2.473E-02	0.252
29	CD4+ TCM	HPCA	-0.621	2.493E-02	0.377
30	MPP	BLUEPRINT	-0.653	2.546E-02	0.304
31	ENDOTHELIAL Cells	BLUEPRINT	0.644	2.589E-02	0.089
32	MV ENDOTHELIAL Cells	FANTOM	0.633	3.715E-02	0.077

33	CD4+ MEMORY T-Cells	IRIS	0.753	4.040E-02	0.073
34	CMP	HPCA	0.687	5.177E-02	0.109
35	CD4+ T-Cells	FANTOM	-0.645	5.247E-02	0.450
36	CD8+ T-Cells	FANTOM	0.662	6.128E-02	0.134
37	PLATELETS	HPCA	0.487	6.338E-02	0.234
38	PLATELETS	HPCA	0.517	6.507E-02	0.207
39	CD4+ NAIVE T-Cells	IRIS	-0.599	6.947E-02	0.528
40	KERATINOCYTES	FANTOM	-0.583	6.987E-02	0.566
41	CD8+ T-Cells	BLUEPRINT	0.662	7.003E-02	0.143
42	KERATINOCYTES	HPCA	-0.602	7.133E-02	0.582
43	EPITHELIAL Cells	ENCODE	-0.578	8.124E-02	0.422
44	EPITHELIAL Cells	HPCA	-0.583	8.272E-02	0.394
45	EPITHELIAL Cells	HPCA	-0.602	8.396E-02	0.468
46	PERICYTES	ENCODE	0.604	8.447E-02	0.181
47	KERATINOCYTES	ENCODE	-0.602	8.465E-02	0.375
48	MACROPHAGES	BLUEPRINT	0.488	8.834E-02	0.272
49	KERATINOCYTES	HPCA	-0.602	8.951E-02	0.508
50	CD4+ TCM	NOVERSHTERN	-0.594	9.222E-02	0.353

P-values were determined by Kolmogorov Smirnov test implemented in GSEA.

Supplementary Table 8. The results from iDEA to detect top 50 enriched gene sets among human gene sets on 10x Genomics PBMC scRNA-seq data

Rank	Gene Set	Count	Coefficient	Variance	*P*-value
1	REACTOME_METABOLISM_OF_PROTEINS	144	3.018	0.041	5.864E-46
2	GO_AMIDE_BIOSYNTHETIC_PROCESS	158	2.603	0.034	5.939E-41
3	GO_ORGANIC_CYCLIC_COMPOUND_CATABOLIC_PROCESS	128	2.877	0.043	2.905E-39
4	HSIAO_HOUSEKEEPING_GENES	268	2.120	0.023	3.706E-39
5	GO_PROTEIN_TARGETING	140	2.675	0.038	4.529E-39
6	GO_VIRAL_LIFE_CYCLE	115	3.192	0.053	9.420E-39
7	GO_ESTABLISHMENT_OF_PROTEIN_LOCALIZATION_TO_ORGANELLE	130	2.821	0.042	1.196E-38
8	GO_RIBOSOME	116	3.045	0.050	1.742E-38
9	REACTOME_TRANSLATION	112	3.777	0.073	2.300E-38
10	REACTOME_METABOLISM_OF_MRNA	125	2.845	0.044	2.377E-38
11	REACTOME_METABOLISM_OF_RNA	132	2.708	0.040	7.892E-38
12	GO_RNA_CATABOLIC_PROCESS	109	3.343	0.060	9.977E-38
13	GO_PROTEIN_LOCALIZATION_TO_MEMBRANE	130	2.751	0.041	1.551E-37
14	GO_RIBONUCLEOPROTEIN_COMPLEX_BIOGENESIS	147	2.512	0.035	2.951E-37
15	GO_ESTABLISHMENT_OF_PROTEIN_LOCALIZATION_TO_MEMBRANE	115	3.070	0.051	3.167E-37
16	GO_RIBOSOME_BIOGENESIS	111	3.069	0.053	4.482E-37
17	GO_STRUCTURAL_CONSTITUENT_OF_RIBOSOME	107	3.225	0.058	9.835E-37
18	GO_NCRNA_PROCESSING	114	2.924	0.049	1.975E-36
19	GSE2405_0H_VS_24H_A_PHAGOCYTOPHILUM_STIM_NEUTROPHIL_UP	142	2.587	0.037	2.119E-36
20	GO_RIBOSOMAL_SUBUNIT	103	3.479	0.068	5.253E-36
21	GO_RRNA_METABOLIC_PROCESS	103	3.342	0.064	1.752E-35
22	GO_NCRNA_METABOLIC_PROCESS	129	2.569	0.040	3.883E-35
23	GO_CYTOSOLIC_PART	101	3.590	0.074	5.188E-35
24	GO_TRANSLATIONAL_INITIATION	104	3.933	0.087	9.055E-35
25	GSE2405_0H_VS_9H_A_PHAGOCYTOPHILUM_STIM_NEUTROPHIL_DN	142	2.450	0.036	4.128E-34
26	GO_PROTEIN_TARGETING_TO_MEMBRANE	97	3.780	0.086	8.355E-33
27	GO_PROTEIN_LOCALIZATION_TO_ENDOPLASMIC_RETICULUM	97	3.855	0.090	4.666E-32
28	GSE41978_ID2_KO_VS_ID2_KO_AND_BIM_KO_KLRG1_LOW_EFFECTOR_CD8_TCELL_DN	109	2.706	0.048	3.589E-30
29	REACTOME_SRP_DEPENDENT_COTRANSLATIONAL_PROTEIN_TARGETING_TO_MEMBRANE	93	4.104	0.111	1.267E-29
30	GSE42088_UNINF_VS_LEISHMANIA_INF_DC_2H_DN	106	2.613	0.048	5.764E-29

31	GSE42088_UNINF_VS_LEISHMANIA_INF_DC_4H_DN	104	2.481	0.048	3.176E-27
32	GSE41978_KLRG1_HIGH_VS_LOW_EFFECTOR_CD8_TCELL_DN	86	2.750	0.061	1.203E-25
33	GSE22886_NAIVE_BCELL_VS_NEUTROPHIL_UP	85	2.619	0.059	6.206E-25
34	GO_CELL_SUBSTRATE_JUNCTION	114	2.229	0.042	6.560E-24
35	GSE22886_NAIVE_TCELL_VS_DC_UP	103	2.279	0.046	9.367E-24
36	GO_ANCHORING_JUNCTION	119	2.153	0.040	1.613E-23
37	PECE_MAMMARY_STEM_CELL_UP	74	2.696	0.069	4.049E-22
38	GO_ESTABLISHMENT_OF_PROTEIN_LOCALIZATION_TO_ENDOPLASMIC_RETICULUM	88	4.633	0.177	5.995E-22
39	OSMAN_BLADDER_CANCER_DN	125	1.925	0.038	3.596E-21
40	GO_LARGE_RIBOSOMAL_SUBUNIT	61	3.320	0.111	7.696E-21
41	JISON_SICKLE_CELL_DISEASE_DN	80	2.351	0.059	3.695E-20
42	GSE34205_HEALTHY_VS_FLU_INF_INFANT_PBMC_UP	70	2.405	0.067	6.569E-19
43	TIEN_INTESTINE_PROBIOTICS_24HR_DN	76	2.252	0.061	1.092E-17
44	GSE3720_UNSTIM_VS_PMA_STIM_VD2_GAMMADELTA_TCELL_UP	73	2.226	0.062	1.620E-17
45	GSE26156_DOUBLE_POSITIVE_VS_CD4_SINGLE_POSITIVE_THYMOCYTE_UP	79	2.099	0.057	7.262E-17
46	GSE14000_TRANSLATED_RNA_VS_MRNA_DC_DN	80	2.044	0.056	1.214E-16
47	GSE21927_SPLEEN_C57BL6_VS_4T1_TUMOR_BALBC_MONOCYTES_UP	77	2.149	0.059	1.407E-16
48	GSE3720_UNSTIM_VS_LPS_STIM_VD2_GAMMADELTA_TCELL_UP	62	2.320	0.074	4.046E-16
49	REACTOME_3_UTR_MEDIATED_TRANSLATIONAL_REGULATION	91	5.580	0.381	1.294E-15
50	GO_NUCLEAR_TRANSCRIBED_MRNA_CATABOLIC_PROCESS_NONSENSE_MEDIATED_DECAY	86	5.305	0.319	1.338E-14

P-values were determined by two-sided Wald test.

Supplementary Table 9. Computation time (minutes) for each GSE method on three real scRNA-seq datasets

Method	Computation Time		
	Human (n=15280,m=12033)	Mouse (n=13598, m =2851)	10x Genomics (n=13713, m =144)
iDEA	21057	3991	24
fGSEA	0.38	0.07	0.01
CAMERA	0.004	0.001	0.0002
PAGE	1.1	0.25	0.13
GSEA	47.4	67.1	0.5

Computation was performed on a single core of an Intel Xeon L5420 2.50 GHz processor. n is number of genes analyzed in the dataset; m is number of gene sets.

Supplementary Table 10. Results for the top first gene set GO:0001944 (vasculature development) with the combinations of the top 50 gene sets in human embryonic stem cell scRNA-seq dataset

	P-value	Adjusted *p*-value	Set	Count	Set2
**1**	5.456E-17	1.970E-13	intersection	332	GO_BLOOD_VESSEL_MORPHOGENESIS
**2**	1.132E-02	1.000E+00	set1	97	GO_BLOOD_VESSEL_MORPHOGENESIS
**3**	NA	NA	set2	0	GO_BLOOD_VESSEL_MORPHOGENESIS
**4**	3.397E-04	1.000E+00	intersection	49	SCHUETZ_BREAST_CANCER_DUCTAL_INVASIVE_UP
**5**	2.937E-15	1.060E-11	set1	380	SCHUETZ_BREAST_CANCER_DUCTAL_INVASIVE_UP
**6**	8.564E-20	3.092E-16	set2	261	SCHUETZ_BREAST_CANCER_DUCTAL_INVASIVE_UP
**7**	1.327E-15	4.793E-12	intersection	268	GO_ANGIOGENESIS
**8**	1.031E-03	1.000E+00	set1	161	GO_ANGIOGENESIS
**9**	NA	NA	set2	0	GO_ANGIOGENESIS
**10**	2.819E-04	1.000E+00	intersection	41	LIU_PROSTATE_CANCER_DN
**11**	1.816E-14	6.557E-11	set1	388	LIU_PROSTATE_CANCER_DN
**12**	9.917E-17	3.581E-13	set2	383	LIU_PROSTATE_CANCER_DN
**13**	4.875E-06	1.760E-02	intersection	57	SWEET_LUNG_CANCER_KRAS_DN
**14**	1.097E-12	3.960E-09	set1	372	SWEET_LUNG_CANCER_KRAS_DN
**15**	4.693E-14	1.695E-10	set2	320	SWEET_LUNG_CANCER_KRAS_DN
**16**	8.046E-03	1.000E+00	intersection	26	ONDER_CDH1_TARGETS_2_DN
**17**	1.916E-16	6.920E-13	set1	403	ONDER_CDH1_TARGETS_2_DN
**18**	5.558E-19	2.007E-15	set2	358	ONDER_CDH1_TARGETS_2_DN
**19**	8.242E-03	1.000E+00	intersection	23	SMID_BREAST_CANCER_NORMAL_LIKE_UP
**20**	3.566E-16	1.288E-12	set1	406	SMID_BREAST_CANCER_NORMAL_LIKE_UP
**21**	5.281E-19	1.907E-15	set2	338	SMID_BREAST_CANCER_NORMAL_LIKE_UP
**22**	1.746E-05	6.305E-02	intersection	64	GO_SINGLE_ORGANISM_CELL_ADHESION
**23**	1.134E-13	4.096E-10	set1	365	GO_SINGLE_ORGANISM_CELL_ADHESION
**24**	5.507E-14	1.989E-10	set2	306	GO_SINGLE_ORGANISM_CELL_ADHESION
**25**	1.982E-04	7.158E-01	intersection	49	GO_ANCHORING_JUNCTION
**26**	2.914E-14	1.052E-10	set1	380	GO_ANCHORING_JUNCTION
**27**	1.095E-13	3.953E-10	set2	419	GO_ANCHORING_JUNCTION
**28**	2.173E-05	7.847E-02	intersection	63	LIM_MAMMARY_STEM_CELL_UP
**29**	1.648E-13	5.950E-10	set1	366	LIM_MAMMARY_STEM_CELL_UP
**30**	2.416E-13	8.723E-10	set2	380	LIM_MAMMARY_STEM_CELL_UP
**31**	7.076E-04	1.000E+00	intersection	38	PASINI_SUZ12_TARGETS_DN
**32**	3.246E-15	1.172E-11	set1	391	PASINI_SUZ12_TARGETS_DN
**33**	2.121E-14	7.658E-11	set2	267	PASINI_SUZ12_TARGETS_DN
**34**	7.560E-02	1.000E+00	intersection	28	BOQUEST_STEM_CELL_DN
**35**	4.837E-14	1.747E-10	set1	401	BOQUEST_STEM_CELL_DN
**36**	2.507E-14	9.053E-11	set2	173	BOQUEST_STEM_CELL_DN
**37**	3.372E-04	1.000E+00	intersection	39	CHARAFE_BREAST_CANCER_LUMINAL_VS_MESENCHYMAL_DN

**38**	2.993E-15	1.081E-11	set1	390	CHARAFE_BREAST_CANCER_LUMINAL_VS_MESENCHYMAL_DN
**39**	2.370E-14	8.558E-11	set2	403	CHARAFE_BREAST_CANCER_LUMINAL_VS_MESENCHYMAL_DN
**40**	9.378E-02	1.000E+00	intersection	15	BENPORATH_ES_1
**41**	2.749E-16	9.927E-13	set1	414	BENPORATH_ES_1
**42**	1.405E-14	5.074E-11	set2	357	BENPORATH_ES_1
**43**	7.779E-05	2.809E-01	intersection	45	GO_REGULATION_OF_SYSTEM_PROCESS
**44**	1.130E-13	4.081E-10	set1	384	GO_REGULATION_OF_SYSTEM_PROCESS
**45**	4.550E-12	1.643E-08	set2	345	GO_REGULATION_OF_SYSTEM_PROCESS
**46**	3.187E-03	1.000E+00	intersection	29	GO_APICAL_PART_OF_CELL
**47**	9.338E-16	3.372E-12	set1	400	GO_APICAL_PART_OF_CELL
**48**	9.476E-15	3.422E-11	set2	252	GO_APICAL_PART_OF_CELL
**49**	1.751E-03	1.000E+00	intersection	52	GO_EXTRACELLULAR_MATRIX
**50**	9.809E-16	3.542E-12	set1	377	GO_EXTRACELLULAR_MATRIX
**51**	6.689E-15	2.415E-11	set2	274	GO_EXTRACELLULAR_MATRIX
**52**	4.220E-03	1.000E+00	intersection	32	GO_CELL_CELL_JUNCTION
**53**	1.702E-14	6.147E-11	set1	397	GO_CELL_CELL_JUNCTION
**54**	3.221E-12	1.163E-08	set2	303	GO_CELL_CELL_JUNCTION
**55**	1.898E-03	1.000E+00	intersection	39	DELYS_THYROID_CANCER_UP
**56**	6.755E-16	2.439E-12	set1	390	DELYS_THYROID_CANCER_UP
**57**	3.937E-15	1.422E-11	set2	340	DELYS_THYROID_CANCER_UP
**58**	2.988E-06	1.079E-02	intersection	68	GO_WOUND_HEALING
**59**	9.462E-13	3.417E-09	set1	361	GO_WOUND_HEALING
**60**	7.381E-10	2.665E-06	set2	325	GO_WOUND_HEALING
**61**	3.173E-02	1.000E+00	intersection	28	RODWELL_AGING_KIDNEY_UP
**62**	3.612E-17	1.304E-13	set1	401	RODWELL_AGING_KIDNEY_UP
**63**	1.729E-15	6.245E-12	set2	390	RODWELL_AGING_KIDNEY_UP
**64**	7.056E-03	1.000E+00	intersection	36	CHARAFE_BREAST_CANCER_LUMINAL_VS_BASAL_DN
**65**	2.316E-16	8.364E-13	set1	393	CHARAFE_BREAST_CANCER_LUMINAL_VS_BASAL_DN
**66**	1.085E-14	3.919E-11	set2	366	CHARAFE_BREAST_CANCER_LUMINAL_VS_BASAL_DN
**67**	6.431E-09	2.322E-05	intersection	103	GO_REGULATION_OF_VASCULATURE_DEVELOPMENT
**68**	1.376E-10	4.971E-07	set1	326	GO_REGULATION_OF_VASCULATURE_DEVELOPMENT
**69**	1.051E-05	3.795E-02	set2	97	GO_REGULATION_OF_VASCULATURE_DEVELOPMENT
**70**	2.385E-04	8.613E-01	intersection	39	SMID_BREAST_CANCER_LUMINAL_B_DN
**71**	1.214E-14	4.385E-11	set1	390	SMID_BREAST_CANCER_LUMINAL_B_DN
**72**	4.096E-12	1.479E-08	set2	389	SMID_BREAST_CANCER_LUMINAL_B_DN
**73**	1.321E-01	1.000E+00	intersection	8	SABATES_COLORECTAL_ADENOMA_DN
**74**	2.425E-17	8.757E-14	set1	421	SABATES_COLORECTAL_ADENOMA_DN
**75**	9.821E-16	3.546E-12	set2	190	SABATES_COLORECTAL_ADENOMA_DN
**76**	6.503E-05	2.348E-01	intersection	40	GO_LEUKOCYTE_MIGRATION
**77**	9.305E-14	3.360E-10	set1	389	GO_LEUKOCYTE_MIGRATION
**78**	8.733E-11	3.153E-07	set2	151	GO_LEUKOCYTE_MIGRATION
**79**	7.538E-04	1.000E+00	intersection	36	LINDGREN_BLADDER_CANCER_CLUSTER_2B

**80**	3.720E-15	1.343E-11	set1	393	LINDGREN_BLADDER_CANCER_CLUSTER_2B
**81**	1.359E-11	4.907E-08	set2	317	LINDGREN_BLADDER_CANCER_CLUSTER_2B
**82**	2.772E-05	1.001E-01	intersection	68	GO_TAXIS
**83**	7.298E-14	2.635E-10	set1	361	GO_TAXIS
**84**	3.585E-10	1.294E-06	set2	298	GO_TAXIS
**85**	7.764E-07	2.804E-03	intersection	73	GO_POSITIVE_REGULATION_OF_MAPK_CASCADE
**86**	3.479E-12	1.256E-08	set1	356	GO_POSITIVE_REGULATION_OF_MAPK_CASCADE
**87**	3.825E-08	1.381E-04	set2	320	GO_POSITIVE_REGULATION_OF_MAPK_CASCADE
**88**	1.614E-02	1.000E+00	intersection	18	CREIGHTON_ENDOCRINE_THERAPY_RESISTANCE_5
**89**	1.110E-15	4.007E-12	set1	411	CREIGHTON_ENDOCRINE_THERAPY_RESISTANCE_5
**90**	2.149E-12	7.758E-09	set2	413	CREIGHTON_ENDOCRINE_THERAPY_RESISTANCE_5
**91**	1.277E-04	4.611E-01	intersection	51	GO_REGULATION_OF_BODY_FLUID_LEVELS
**92**	1.104E-12	3.986E-09	set1	378	GO_REGULATION_OF_BODY_FLUID_LEVELS
**93**	7.002E-09	2.528E-05	set2	359	GO_REGULATION_OF_BODY_FLUID_LEVELS
**94**	1.332E-03	1.000E+00	intersection	49	GO_REGULATION_OF_NEURON_PROJECTION_DEVELOPMENT
**95**	1.639E-15	5.917E-12	set1	380	GO_REGULATION_OF_NEURON_PROJECTION_DEVELOPMENT
**96**	1.633E-11	5.896E-08	set2	315	GO_REGULATION_OF_NEURON_PROJECTION_DEVELOPMENT
**97**	3.274E-03	1.000E+00	intersection	37	DUTERTRE_ESTRADIOL_RESPONSE_24HR_DN
**98**	7.166E-16	2.588E-12	set1	392	DUTERTRE_ESTRADIOL_RESPONSE_24HR_DN
**99**	1.579E-12	5.703E-09	set2	450	DUTERTRE_ESTRADIOL_RESPONSE_24HR_DN
**100**	1.335E-03	1.000E+00	intersection	30	GO_MEMBRANE_MICRODOMAIN
**101**	1.734E-14	6.261E-11	set1	399	GO_MEMBRANE_MICRODOMAIN
**102**	5.558E-10	2.007E-06	set2	229	GO_MEMBRANE_MICRODOMAIN
**103**	6.314E-03	1.000E+00	intersection	17	GSE2405_0H_VS_3H_A_PHAGOCYTOPHILUM_STIM_NEUTROPHIL_UP
**104**	2.400E-16	8.665E-13	set1	412	GSE2405_0H_VS_3H_A_PHAGOCYTOPHILUM_STIM_NEUTROPHIL_UP
**105**	3.248E-13	1.173E-09	set2	161	GSE2405_0H_VS_3H_A_PHAGOCYTOPHILUM_STIM_NEUTROPHIL_UP
**106**	6.100E-02	1.000E+00	intersection	11	WANG_RESPONSE_TO_GSK3_INHIBITOR_SB216763_DN
**107**	3.474E-17	1.255E-13	set1	418	WANG_RESPONSE_TO_GSK3_INHIBITOR_SB216763_DN
**108**	3.274E-12	1.182E-08	set2	329	WANG_RESPONSE_TO_GSK3_INHIBITOR_SB216763_DN
**109**	2.077E-03	1.000E+00	intersection	37	GO_CELL_SUBSTRATE_JUNCTION
**110**	8.636E-16	3.118E-12	set1	392	GO_CELL_SUBSTRATE_JUNCTION
**111**	4.238E-11	1.530E-07	set2	348	GO_CELL_SUBSTRATE_JUNCTION
**112**	6.744E-02	1.000E+00	intersection	30	GO_CELL_LEADING_EDGE
**113**	2.993E-17	1.081E-13	set1	399	GO_CELL_LEADING_EDGE
**114**	5.629E-13	2.033E-09	set2	293	GO_CELL_LEADING_EDGE
**115**	1.849E-04	6.677E-01	intersection	45	REACTOME_HEMOSTASIS
**116**	3.583E-13	1.294E-09	set1	384	REACTOME_HEMOSTASIS
**117**	3.356E-08	1.212E-04	set2	341	REACTOME_HEMOSTASIS
**118**	9.548E-05	3.448E-01	intersection	48	GO_NEGATIVE_REGULATION_OF_LOCOMOTION
**119**	6.438E-14	2.325E-10	set1	381	GO_NEGATIVE_REGULATION_OF_LOCOMOTION

**120**	1.304E-07	4.708E-04	set2	179	GO_NEGATIVE_REGULATION_OF_LOCOMOTION
**121**	9.589E-03	1.000E+00	intersection	28	GO_ACTIN_FILAMENT_BASED_PROCESS
**122**	1.619E-16	5.845E-13	set1	401	GO_ACTIN_FILAMENT_BASED_PROCESS
**123**	3.258E-12	1.177E-08	set2	380	GO_ACTIN_FILAMENT_BASED_PROCESS
**124**	1.755E-03	1.000E+00	intersection	29	SCHAEFFER_PROSTATE_DEVELOPMENT_48HR_UP
**125**	8.673E-16	3.132E-12	set1	400	SCHAEFFER_PROSTATE_DEVELOPMENT_48HR_UP
**126**	2.443E-11	8.820E-08	set2	396	SCHAEFFER_PROSTATE_DEVELOPMENT_48HR_UP
**127**	1.955E-04	7.059E-01	intersection	57	GO_POSITIVE_REGULATION_OF_CELL_DEVELOPMENT
**128**	8.814E-15	3.183E-11	set1	372	GO_POSITIVE_REGULATION_OF_CELL_DEVELOPMENT
**129**	8.851E-10	3.196E-06	set2	350	GO_POSITIVE_REGULATION_OF_CELL_DEVELOPMENT
**130**	2.947E-07	1.064E-03	intersection	74	GO_CELL_MORPHOGENESIS_INVOLVED_IN_DIFFERENTIATION
**131**	5.398E-12	1.949E-08	set1	355	GO_CELL_MORPHOGENESIS_INVOLVED_IN_DIFFERENTIATION
**132**	5.068E-07	1.830E-03	set2	382	GO_CELL_MORPHOGENESIS_INVOLVED_IN_DIFFERENTIATION
**133**	7.329E-03	1.000E+00	intersection	49	GO_POSITIVE_REGULATION_OF_NERVOUS_SYSTEM_DEVELOP MENT
**134**	2.761E-16	9.968E-13	set1	380	GO_POSITIVE_REGULATION_OF_NERVOUS_SYSTEM_DEVELOP MENT
**135**	3.507E-11	1.266E-07	set2	325	GO_POSITIVE_REGULATION_OF_NERVOUS_SYSTEM_DEVELOP MENT
**136**	4.344E-02	1.000E+00	intersection	13	CHYLA_CBFA2T3_TARGETS_UP
**137**	4.305E-17	1.555E-13	set1	416	CHYLA_CBFA2T3_TARGETS_UP
**138**	4.758E-13	1.718E-09	set2	311	CHYLA_CBFA2T3_TARGETS_UP
**139**	1.896E-02	1.000E+00	intersection	19	VERHAAK_AML_WITH_NPM1_MUTATED_DN
**140**	2.985E-16	1.078E-12	set1	410	VERHAAK_AML_WITH_NPM1_MUTATED_DN
**141**	3.932E-12	1.420E-08	set2	186	VERHAAK_AML_WITH_NPM1_MUTATED_DN
**142**	7.166E-05	2.588E-01	intersection	52	GO_REGULATION_OF_CELL_MORPHOGENESIS_INVOLVED_IN_ DIFFERENTIATION
**143**	1.009E-14	3.645E-11	set1	377	GO_REGULATION_OF_CELL_MORPHOGENESIS_INVOLVED_IN_ DIFFERENTIATION
**144**	8.985E-09	3.244E-05	set2	252	GO_REGULATION_OF_CELL_MORPHOGENESIS_INVOLVED_IN_ DIFFERENTIATION
**145**	5.122E-02	1.000E+00	intersection	12	WALLACE_PROSTATE_CANCER_RACE_UP
**146**	3.360E-17	1.213E-13	set1	417	WALLACE_PROSTATE_CANCER_RACE_UP
**147**	1.301E-12	4.698E-09	set2	220	WALLACE_PROSTATE_CANCER_RACE_UP

From the second gene set to the 50th gene set, we calculate the adjusted *p*-values for their intersection with the top first gene set as well as the disjoint parts. *P*-values were determined by two-sided Wald test and adjusted by Bonferroni correction.

Supplementary Table 11. Results for the top first gene set GO:0044425 (obsolete membrane part) with the combinations of the top 50 gene sets in mouse neuron cell scRNA-seq dataset

	P-value	Adjusted *p*-value	Set	Count	Set2
1	5.530E-40	1.865E-36	intersection	674	GO:0043005
2	4.480E-22	1.511E-18	dis1	3014	GO:0043005
3	4.015E-10	1.354E-06	dis2	447	GO:0043005
4	1.497E-50	5.047E-47	intersection	2021	GO:0071944
5	1.346E-06	4.538E-03	dis1	1667	GO:0071944
6	2.991E-02	1.000E+00	dis2	819	GO:0071944
7	2.696E-55	9.092E-52	intersection	3688	GO:0016020
8	NA	NA	dis1	0	GO:0016020
9	9.927E-01	1.000E+00	dis2	1393	GO:0016020
10	1.179E-40	3.975E-37	intersection	878	GO:0097458
11	3.187E-19	1.075E-15	dis1	2810	GO:0097458
12	2.933E-09	9.890E-06	dis2	594	GO:0097458
13	1.920E-51	6.474E-48	intersection	2011	GO:0005886
14	2.378E-06	8.018E-03	dis1	1677	GO:0005886
15	6.089E-02	1.000E+00	dis2	751	GO:0005886
16	3.846E-42	1.297E-38	intersection	2845	GO:0031224
17	2.248E-09	7.580E-06	dis1	843	GO:0031224
18	NA	NA	dis2	0	GO:0031224
19	5.979E-39	2.016E-35	intersection	1498	GO:0044459
20	1.320E-14	4.452E-11	dis1	2190	GO:0044459
21	NA	NA	dis2	0	GO:0044459
22	1.269E-36	4.278E-33	intersection	731	GO:0045202
23	3.134E-23	1.057E-19	dis1	2957	GO:0045202
24	1.383E-05	4.665E-02	dis2	377	GO:0045202
25	8.956E-33	3.020E-29	intersection	801	GO:0006811
26	1.326E-23	4.470E-20	dis1	2887	GO:0006811
27	6.015E-07	2.028E-03	dis2	177	GO:0006811
28	1.192E-33	4.018E-30	intersection	630	GO:0044456
29	7.006E-26	2.363E-22	dis1	3058	GO:0044456
30	1.153E-05	3.888E-02	dis2	237	GO:0044456
31	3.137E-37	1.058E-33	intersection	2758	GO:0016021
32	1.562E-13	5.266E-10	dis1	930	GO:0016021
33	NA	NA	dis2	0	GO:0016021
34	4.903E-30	1.653E-26	intersection	578	GO:0006812

**35**	9.523E-28	3.211E-24	dis1	3110	GO:0006812
**36**	5.887E-07	1.985E-03	dis2	121	GO:0006812
**37**	9.344E-32	3.151E-28	intersection	293	GO:0030424
**38**	3.914E-33	1.320E-29	dis1	3395	GO:0030424
**39**	5.490E-06	1.851E-02	dis2	158	GO:0030424
**40**	4.050E-38	1.366E-34	intersection	862	GO:0120025
**41**	1.999E-20	6.740E-17	dis1	2826	GO:0120025
**42**	1.721E-03	1.000E+00	dis2	726	GO:0120025
**43**	2.452E-28	8.269E-25	intersection	475	GO:0036477
**44**	2.004E-30	6.757E-27	dis1	3213	GO:0036477
**45**	4.887E-07	1.648E-03	dis2	308	GO:0036477
**46**	2.091E-30	7.052E-27	intersection	256	GO:0015672
**47**	4.665E-34	1.573E-30	dis1	3432	GO:0015672
**48**	1.107E-03	1.000E+00	dis2	50	GO:0015672
**49**	5.395E-31	1.819E-27	intersection	101	GO:0098800
**50**	4.154E-39	1.401E-35	dis1	3587	GO:0098800
**51**	NA	NA	dis2	0	GO:0098800
**52**	3.955E-37	1.334E-33	intersection	905	GO:0042995
**53**	1.176E-20	3.964E-17	dis1	2783	GO:0042995
**54**	7.495E-03	1.000E+00	dis2	821	GO:0042995
**55**	3.923E-29	1.323E-25	intersection	360	GO:0043269
**56**	2.379E-32	8.022E-29	dis1	3328	GO:0043269
**57**	1.795E-03	1.000E+00	dis2	106	GO:0043269
**58**	3.216E-29	1.085E-25	intersection	117	GO:0098798
**59**	6.701E-40	2.259E-36	dis1	3571	GO:0098798
**60**	9.999E-01	1.000E+00	dis2	9	GO:0098798
**61**	2.585E-17	8.716E-14	intersection	113	GO:0043209
**62**	9.816E-44	3.310E-40	dis1	3575	GO:0043209
**63**	2.775E-13	9.358E-10	dis2	66	GO:0043209
**64**	4.657E-31	1.570E-27	intersection	1012	GO:0032879
**65**	1.029E-22	3.471E-19	dis1	2676	GO:0032879
**66**	4.442E-03	1.000E+00	dis2	911	GO:0032879
**67**	4.250E-29	1.433E-25	intersection	780	GO:0051049
**68**	3.641E-26	1.228E-22	dis1	2908	GO:0051049
**69**	1.794E-03	1.000E+00	dis2	527	GO:0051049
**70**	3.048E-27	1.028E-23	intersection	181	GO:0044455
**71**	1.043E-38	3.516E-35	dis1	3507	GO:0044455
**72**	NA	NA	dis2	0	GO:0044455

**73**	1.869E-24	6.304E-21	intersection	480	GO:0034220
**74**	1.894E-32	6.386E-29	dis1	3208	GO:0034220
**75**	1.537E-03	1.000E+00	dis2	80	GO:0034220
**76**	3.455E-26	1.165E-22	intersection	785	GO:0031226
**77**	7.877E-28	2.656E-24	dis1	2903	GO:0031226
**78**	NA	NA	dis2	0	GO:0031226
**79**	5.315E-23	1.792E-19	intersection	360	GO:0030425
**80**	1.155E-35	3.896E-32	dis1	3328	GO:0030425
**81**	5.021E-05	1.693E-01	dis2	197	GO:0030425
**82**	1.638E-25	5.525E-22	intersection	351	GO:0098793
**83**	4.132E-34	1.393E-30	dis1	3337	GO:0098793
**84**	2.928E-02	1.000E+00	dis2	93	GO:0098793
**85**	1.611E-22	5.432E-19	intersection	373	GO:0099536
**86**	1.186E-35	4.001E-32	dis1	3315	GO:0099536
**87**	1.159E-04	3.910E-01	dis2	153	GO:0099536
**88**	5.901E-25	1.990E-21	intersection	785	GO:0098590
**89**	3.558E-28	1.200E-24	dis1	2903	GO:0098590
**90**	NA	NA	dis2	0	GO:0098590
**91**	1.415E-20	4.771E-17	intersection	241	GO:0042391
**92**	4.186E-39	1.411E-35	dis1	3447	GO:0042391
**93**	2.728E-05	9.198E-02	dis2	75	GO:0042391
**94**	5.804E-25	1.957E-21	intersection	742	GO:0005887
**95**	3.588E-29	1.210E-25	dis1	2946	GO:0005887
**96**	NA	NA	dis2	0	GO:0005887
**97**	5.966E-23	2.012E-19	intersection	371	GO:0098660
**98**	6.335E-35	2.136E-31	dis1	3317	GO:0098660
**99**	8.762E-03	1.000E+00	dis2	59	GO:0098660
**100**	1.034E-21	3.488E-18	intersection	367	GO:0099537
**101**	6.286E-36	2.120E-32	dis1	3321	GO:0099537
**102**	1.136E-04	3.831E-01	dis2	151	GO:0099537
**103**	6.685E-23	2.254E-19	intersection	393	GO:0008324
**104**	8.238E-35	2.778E-31	dis1	3295	GO:0008324
**105**	1.176E-02	1.000E+00	dis2	11	GO:0008324
**106**	3.790E-21	1.278E-17	intersection	359	GO:0098916
**107**	1.765E-36	5.952E-33	dis1	3329	GO:0098916
**108**	1.189E-04	4.008E-01	dis2	151	GO:0098916
**109**	7.078E-21	2.387E-17	intersection	359	GO:0007268
**110**	2.232E-36	7.526E-33	dis1	3329	GO:0007268

111	9.247E-05	3.118E-01	dis2	151	GO:0007268
112	2.722E-02	1.000E+00	intersection	330	GO:0090304
113	3.493E-53	1.178E-49	dis1	3358	GO:0090304
114	1.120E-27	3.776E-24	dis2	2777	GO:0090304
115	7.064E-22	2.382E-18	intersection	683	GO:0055085
116	8.823E-32	2.975E-28	dis1	3005	GO:0055085
117	1.605E-02	1.000E+00	dis2	102	GO:0055085
118	4.873E-22	1.643E-18	intersection	342	GO:0098662
119	4.432E-36	1.494E-32	dis1	3346	GO:0098662
120	6.972E-03	1.000E+00	dis2	58	GO:0098662
121	1.646E-23	5.549E-20	intersection	218	GO:0015077
122	8.758E-38	2.953E-34	dis1	3470	GO:0015077
123	8.318E-01	1.000E+00	dis2	4	GO:0015077
124	6.827E-21	2.302E-17	intersection	383	GO:0098655
125	2.886E-36	9.730E-33	dis1	3305	GO:0098655
126	1.314E-03	1.000E+00	dis2	68	GO:0098655
127	1.083E-23	3.652E-20	intersection	63	GO:0070469
128	4.485E-44	1.512E-40	dis1	3625	GO:0070469
129	NA	NA	dis2	0	GO:0070469
130	4.109E-23	1.386E-19	intersection	395	GO:0097060
131	3.080E-35	1.039E-31	dis1	3293	GO:0097060
132	NA	NA	dis2	0	GO:0097060
133	3.118E-21	1.051E-17	intersection	533	GO:0003008
134	1.141E-33	3.846E-30	dis1	3155	GO:0003008
135	1.467E-04	4.948E-01	dis2	442	GO:0003008
136	4.334E-37	1.462E-33	intersection	1475	GO:0023052
137	8.699E-16	2.933E-12	dis1	2213	GO:0023052
138	6.475E-01	1.000E+00	dis2	1929	GO:0023052
139	1.886E-22	6.361E-19	intersection	60	GO:0005746
140	1.995E-44	6.726E-41	dis1	3628	GO:0005746
141	NA	NA	dis2	0	GO:0005746
142	9.501E-01	1.000E+00	intersection	97	GO:0003676
143	1.564E-56	5.274E-53	dis1	3591	GO:0003676
144	1.962E-22	6.615E-19	dis2	2122	GO:0003676
145	2.839E-17	9.573E-14	intersection	303	GO:0007186
146	1.206E-39	4.065E-36	dis1	3385	GO:0007186
147	6.985E-06	2.355E-02	dis2	90	GO:0007186

From the second gene set to the 50th gene set, we calculate the adjusted *p*-values for their intersection with the top first gene set as well as the disjoint parts. *P*-values were determined by two-sided Wald test and adjusted by Bonferroni correction.

Supplementary References

1 Conway, J. R., Lex, A. & Gehlenborg, N. UpSetR: an R package for the visualization of intersecting sets and their properties. *Bioinformatics* **33**, 2938-2940, doi:10.1093/bioinformatics/btx364 (2017).

2 Louis, T. A. Finding the Observed Information Matrix When Using the Em Algorithm. *J Roy Stat Soc B Met* **44**, 226-233 (1982).

3 Oakes, D. Direct calculation of the information matrix via the EM algorithm. *J Roy Stat Soc B* **61**, 479-482, doi:Doi 10.1111/1467-9868.00188 (1999).

4 Tuch, B. B. *et al.* Tumor transcriptome sequencing reveals allelic expression imbalances associated with copy number alterations. *PLoS One* **5**, e9317, doi:10.1371/journal.pone.0009317 (2010).

5 Shah, M. Y. & Mehta, A. R. Metastasis from breast cancer presenting as an epulis in the upper gingiva. *J Oral Maxillofac Pathol* **13**, 38-40, doi:10.4103/0973-029X.48756 (2009).

6 Daly, M. E. *et al.* Intensity-modulated radiotherapy for locally advanced cancers of the larynx and hypopharynx. *Head Neck* **33**, 103-111, doi:10.1002/hed.21406 (2011).

7 Pickup, M. W., Mouw, J. K. & Weaver, V. M. The extracellular matrix modulates the hallmarks of cancer. *EMBO Rep* **15**, 1243-1253, doi:10.15252/embr.201439246 (2014).

8 Mazzoccoli, G. *et al.* A primary tumor gene expression signature identifies a crucial role played by tumor stroma myofibroblasts in lymph node involvement in oral squamous cell carcinoma. *Oncotarget* **8**, 104913-104927, doi:10.18632/oncotarget.20645 (2017).

9 Usoskin, D. *et al.* Unbiased classification of sensory neuron types by large-scale single-cell RNA sequencing. *Nat Neurosci* **18**, 145-153, doi:10.1038/nn.3881 (2015).

10 Salahshourifar, I. *et al.* Downregulation of CRNN gene and genomic instability at 1q21.3 in oral squamous cell carcinoma. *Clin Oral Investig* **19**, 2273-2283, doi:10.1007/s00784-015-1467-7 (2015).

11 Uraguchi, M., Morikawa, M., Shirakawa, M., Sanada, K. & Imai, K. Activation of WNT family expression and signaling in squamous cell carcinomas of the oral cavity. *J Dent Res* **83**, 327-332, doi:10.1177/154405910408300411 (2004).

12 Lv, Z. *et al.* Parathyroid hormone-related protein serves as a prognostic indicator in oral squamous cell carcinoma. *J Exp Clin Cancer Res* **33**, 100, doi:10.1186/s13046-014-0100-y (2014).

13 Harris, T. M. *et al.* Proteomic analysis of oral cavity squamous cell carcinoma specimens identifies patient outcome-associated proteins. *Arch Pathol Lab Med* **139**, 494-507, doi:10.5858/arpa.2014-0131-OA (2015).

14 Zhi, X. *et al.* Expression levels of insulin-like growth factors 1 and 2 in head and neck squamous cell carcinoma. *Growth Horm IGF Res* **24**, 137-141, doi:10.1016/j.ghir.2014.04.003 (2014).

15 Akiyama, K. *et al.* The F-prostaglandin receptor is a novel marker for tumor endothelial cells in renal cell carcinoma. *Pathol Int* **63**, 37-44, doi:10.1111/pin.12031 (2013).
16 Cheng, C. W. *et al.* Loss of GDF10/BMP3b as a prognostic marker collaborates with TGFBR3 to enhance chemotherapy resistance and epithelial-mesenchymal transition in oral squamous cell carcinoma. *Mol Carcinog* **55**, 499-513, doi:10.1002/mc.22297 (2016).
17 Guo, J. B. *et al.* Network and pathway-based analysis of microRNA role in neuropathic pain in rat models. *J Cell Mol Med* **23**, 4534-4544, doi:10.1111/jcmm.14357 (2019).
18 Minis, A. *et al.* Subcellular transcriptomics-dissection of the mRNA composition in the axonal compartment of sensory neurons. *Dev Neurobiol* **74**, 365-381, doi:10.1002/dneu.22140 (2014).
19 Gilman, S. R. *et al.* Rare de novo variants associated with autism implicate a large functional network of genes involved in formation and function of synapses. *Neuron* **70**, 898-907, doi:10.1016/j.neuron.2011.05.021 (2011).
20 Hubel, K. A. Intestinal nerves and ion transport: stimuli, reflexes, and responses. *Am J Physiol* **248**, G261-271, doi:10.1152/ajpgi.1985.248.3.G261 (1985).
21 Jiang, Z. & Gentleman, R. Extensions to gene set enrichment. *Bioinformatics* **23**, 306-313, doi:10.1093/bioinformatics/btl599 (2007).
22 Baron, C. S. *et al.* Cell Type Purification by Single-Cell Transcriptome-Trained Sorting. *Cell* **179**, 527-542 e519, doi:10.1016/j.cell.2019.08.006 (2019).
