# Supplementary material for: Integrative differential expression and gene set enrichment analysis using summary statistics for scRNA-seq studies
Source: Nat Commun. 2020 Mar 27;11:1585. doi: 10.1038/s41467-020-15298-6 (PMC7101316; doi:10.1038/s41467-020-15298-6)
Supplement: Supplementary file 3 — Reporting Summary [file 41467_2020_15298_MOESM3_ESM.pdf]

natureresearch

Corresponding author(s): Xiang Zhou

Last updated by author(s): Jan 26, 2020

Reporting Summary

Nature Research wishes to improve the reproducibility of the work that we publish. This form provides structure for consistency and transparency in reporting. For further information on Nature Research policies, see Authors & Referees and the Editorial Policy Checklist .

Statistics

For all statistical analyses, confirm that the following items are present in the figure legend, table legend, main text, or Methods section.

n/a	Confirmed	
☐	☒	The exact sample size (*n*) for each experimental group/condition, given as a discrete number and unit of measurement
☐	☒	A statement on whether measurements were taken from distinct samples or whether the same sample was measured repeatedly
☐	☒	The statistical test(s) used AND whether they are one- or two-sided<br>*Only common tests should be described solely by name; describe more complex techniques in the Methods section.*
☐	☒	A description of all covariates tested
☐	☒	A description of any assumptions or corrections, such as tests of normality and adjustment for multiple comparisons
☐	☒	A full description of the statistical parameters including central tendency (e.g. means) or other basic estimates (e.g. regression coefficient) AND variation (e.g. standard deviation) or associated estimates of uncertainty (e.g. confidence intervals)
☐	☒	For null hypothesis testing, the test statistic (e.g. *F*, *t*, *r*) with confidence intervals, effect sizes, degrees of freedom and *P* value noted<br>*Give P values as exact values whenever suitable.*
☐	☒	For Bayesian analysis, information on the choice of priors and Markov chain Monte Carlo settings
☐	☒	For hierarchical and complex designs, identification of the appropriate level for tests and full reporting of outcomes
☐	☒	Estimates of effect sizes (e.g. Cohen's *d*, Pearson's *r*), indicating how they were calculated

Our web collection on statistics for biologists contains articles on many of the points above.

Software and code

Policy information about availability of computer code

Data collection	No software is used in data collecting
Data analysis	We used the newly developed R package iDEA for data analysis. iDEA is described in the Methods section and deposited at github [https://github.com/xzhoulab/iDEA]. In addition, we also used the following software for comparative analysis.<br>MAST[https://github.com/RGLab/MAST](R version 1.8.1): a statistical method for assessing transcriptional changes and characterizing heterogeneity in single-cell RNA sequencing data<br>zingeR[https://github.com/statOmics/zingeR](R version 1.0): A zero-inflated negative binomial model based method for scRNAseq differential expression analysis.<br>edgeR[https://bioconductor.org/packages/release/bioc/html/edgeR.html](R version 3.8): a Bioconductor package for differential expression analysis in gene expression data.<br>DESeq2[https://bioconductor.org/packages/release/bioc/html/DESeq2.html](R version 1.18.1): a statistical method for differential expression analysis based on negative binomial model.<br>fGSEA[https://bioconductor.org/packages/release/bioc/html/fgsea.html](R version 1.8.0): A fast method performing gene set enrichment analysis.<br>limma[https://bioconductor.org/packages/release/bioc/html/limma.html](R version 3.8.3): we used the function camera inside the limma package to do the competitive gene set testing accounting for inter-gene correlation.<br>PGSEA[https://bioconductor.org/packages/release/bioc/html/PGSEA.html](R version 1.56.0): A Bioconductor package for parametric gene set enrichment analysis.<br>GSEA[https://www.gsea-msigdb.org/gsea/downloads.jsp](Java version 2.2.4): A knowledge-based approach for interpreting the gene expression data through gene set enrichment analysis.

For manuscripts utilizing custom algorithms or software that are central to the research but not yet described in published literature, software must be made available to editors/reviewers. We strongly encourage code deposition in a community repository (e.g. GitHub). See the Nature Research guidelines for submitting code & software for further information.

Data

Policy information about availability of data

All manuscripts must include a data availability statement. This statement should provide the following information, where applicable:

- Accession codes, unique identifiers, or web links for publicly available datasets
- A list of figures that have associated raw data
- A description of any restrictions on data availability

This study was a reanalysis of existing data, which is openly available at locations provided on the Methods section and Data availability section. The detailed data downloading sites are:
Human embryonic stem cell scRNAseq dataset: [https://www.ncbi.nlm.nih.gov/geo/query/acc.cgi?acc=GSE75748]
Mouse Sensory neuron scRNAseq dataset: [http://linnarssonlab.org/drg/]
10x Genomics PBMC scRNA-seq dataset: [https://support.10xgenomics.com/single-cell-gene-expression/datasets/1.1.0/pbmc3k]
Human gene sets: [http://software.broadinstitute.org/gsea/downloads.jsp]
Mouse Gene Ontology Terms: [http://www.informatics.jax.org/downloads/reports/index.html#go]

Field-specific reporting

Please select the one below that is the best fit for your research. If you are not sure, read the appropriate sections before making your selection.

- [x] Life sciences
- [ ] Behavioural & social sciences
- [ ] Ecological, evolutionary & environmental sciences

For a reference copy of the document with all sections, see nature.com/documents/nr-reporting-summary-flat.pdf

Life sciences study design

All studies must disclose on these points even when the disclosure is negative.

Sample size	No data collection was involved in the present study. All sample size determination information, if available, were described in the original paper for the used data sets.
Data exclusions	For the analysis in Human embryonic dataset, we filtered out lowly expressed genes that have more than 5 counts in at most two cells to obtain summary statistics.<br>For the analysis in Mouse Neuronal dataset, we followed the zingeR pipeline at [https://github.com/statOmics/zinbwaveZinger/blob/master/realdata/usoskin/deAnalysis.Rmd] and filtered out lowly expressed genes that have more than 5 counts in at most two cells to obtain the summary statistics.<br>For the analysis in 10x PBMC scRNAseq dataset, we used Seurat package following the tutorial (https://satijalab.org/seurat/pbmc3k_tutorial.html).<br>For human gene sets we analyzed, we merged the gene sets with summary statistics we obtained and then filtered out the gene sets that contain less than 20 genes. For mouse GO terms, we merged the gene sets with summary statistics we obtained and then filtered out the gene sets that contain less than 50 genes.
Replication	We followed standard practice to compared the type I error control by splitting the dataset within the same cell type and replicated this ten times. We did this to check the variability as well as stability of type I error rate control of each methods we compared.
Randomization	No data collection was involved in the present study. All sample size determination information, if available, were described in the original paper for the used data sets.
Blinding	No data collection was involved in the present study. All sample size determination information, if available, were described in the original paper for the used data sets.

Reporting for specific materials, systems and methods

We require information from authors about some types of materials, experimental systems and methods used in many studies. Here, indicate whether each material, system or method listed is relevant to your study. If you are not sure if a list item applies to your research, read the appropriate section before selecting a response.

Materials & experimental systems

n/a	Involved in the study
☒	☐ Antibodies
☒	☐ Eukaryotic cell lines
☒	☐ Palaeontology
☒	☐ Animals and other organisms
☒	☐ Human research participants
☒	☐ Clinical data

Methods

n/a	Involved in the study
☒	☐ ChIP-seq
☒	☐ Flow cytometry
☒	☐ MRI-based neuroimaging
